# 45S rDNA Diversity *In Natura* as One Step towards Ribosomal Heterogeneity in *Arabidopsis thaliana*

**DOI:** 10.3390/plants12142722

**Published:** 2023-07-21

**Authors:** Valérie Delorme-Hinoux, Assane Mbodj, Sophie Brando, Anne De Bures, Christel Llauro, Fabrice Covato, Joseph Garrigue, Claude Guisset, Jacques Borrut, Marie Mirouze, Jean-Philippe Reichheld, Julio Sáez-Vásquez

**Affiliations:** 1 Université de Perpignan Via Domitia (UPVD), Laboratoire Génome et Développement des Plantes (LGDP), UMR 5096, 66860 Perpignan, France; 2 Centre National de la Recherche Scientifique, Laboratoire Génome et Développement des Plantes (LGDP), UMR 5096, 66860 Perpignan, France; 3 EMR LGDP/MANGO, Mechanisms of Adaptation and Genomics, IRD-CNRS-UPVD, 66860 Perpignan, France; 4 Association Charles Flahault, 66350 Toulouges, France; 5 Institut de Recherche pour le Développement (IRD), ECOBIO, 34000 Montpellier, France; 6 FRNC, Fédération des Réserves Naturelles Catalanes, 66500 Prades, France

**Keywords:** rDNA, *Arabidopsis thaliana*, brassicaceae, ribosomal heterogeneity, natural variation

## Abstract

The keystone of ribosome biogenesis is the transcription of 45S rDNA. The *Arabidopsis thaliana* genome contains hundreds of 45S rDNA units; however, they are not all transcribed. Notably, 45S rDNA units contain insertions/deletions revealing the existence of heterogeneous rRNA genes and, likely, heterogeneous ribosomes for rRNAs. In order to obtain an overall picture of 45S rDNA diversity sustaining the synthesis of rRNAs and, subsequently, of ribosomes *in natura*, we took advantage of 320 new occurrences of *Arabidopsis thaliana* as a metapopulation named At66, sampled from 0 to 1900 m of altitude in the eastern Pyrenees in France. We found that the 45S rDNA copy number is very dynamic *in natura* and identified new genotypes for both 5′ and 3′ External Transcribed Spacers (ETS). Interestingly, the highest 5′ETS genotype diversity is found in altitude while the highest 3′ETS genotype diversity is found at sea level. Structural analysis of 45S rDNA also shows conservation *in natura* of specific 5′ETS and 3′ETS sequences/features required to control rDNA expression and the processing of rRNAs. In conclusion, At66 is a worthwhile natural laboratory, and unraveled 45S rDNA diversity represents an interesting starting material to select subsets for rDNA transcription and alter the rRNA composition of ribosomes both intra- and inter-site.

## 1. Introduction

**Figure 1:**
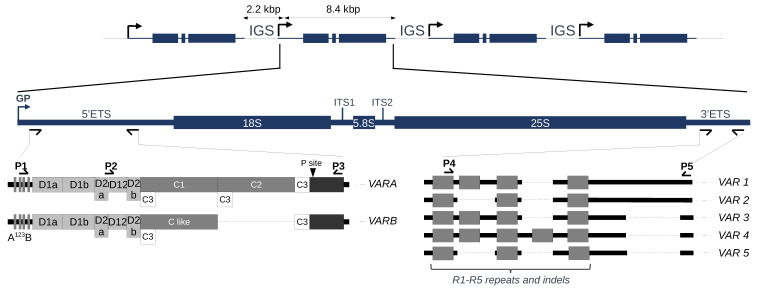
**45S rDNA organization in *the Arabidopsis thaliana* Col-0 accession.** The figure illustrates schematic representations of rDNA units separated by IGSs, 45S rDNA precursor transcript starting at the gene promoter (GP) and 5′ETS (*VARA-B*) and 3′ETS (*VAR1-5*) variants. IGS, intergenic spacers; ETS, External Transcribed Spacers; ITS, Internal Transcribed Spacers. The conserved U3 snoRNP binding site A^123^B and the primary processing site P are indicated. Deletions are represented as dotted lines. Primers used for PCR genotyping or variant sequencing are indicated by arrowheads, P1–P3 for 5′ETS and P4–P5 for 3′ETS. The P2 primer was only used for 5′ETS variant sequencing when appropriate.

Ribosomes are responsible for translating the genetic information residing in mRNAs into proteins and are built from four types of ribosomal RNAs (18S, 5.8S, 25S and 5S rRNAs) and approximately 80 ribosomal proteins [1]. Increasing evidence across various model organisms has demonstrated the heterogeneous nature of ribosomes, and *Arabidopsis thaliana* is a highly suitable model to study this phenomenon [2,3]. In *A. thaliana* Col-0 (Figure 1), the 45S rDNA precursor transcript of 8,4 kbp contains structural rRNAs 18S, 5.8S and 25S flanked by External Transcribed Spacers (5′ETS and 3′ETS) separated by Internal Transcribed Spacers (ITS1 and ITS2) [4,5,6,7]. In all kingdoms, coding sequences for these three rRNAs are highly conserved, but the sequences that are removed during processing (including the ETS and ITS) show a great extent of variation both in length and organization [4,8,9]. Variation along the 45S rDNA precursor transcript using the available 1135 *A. thaliana* genomes also identified polymorphisms within the highly conserved rRNA genes [10,11,12], suggesting that ribosome heterogeneity could involve the sequence variation of rRNAs.

The *A. thaliana* Col-0 accession counts ~800 copies of 45S rDNA units per haploid genome [12,13,14]. These copies are localized in tandem arrays in a head-to-tail manner at chromosomal loci known as the Nucleolar Organizer Regions (NOR2 and 4) at the top of chromosomes 2 and 4 [8,12,13,14]. They are the so-called NORs because transcription and pre-RNA processing allow the nucleolus to form during the interphase of the cell cycle [15,16]. Both NORs have a similar number of rDNA copies in Col-0 and Ler accessions [14,17] but can also vary in size [12,18], and copy number variation was reported in the range of 500–2500 in Swedish accessions [18,19]. Although rRNA transcripts represent the majority of all transcribed RNAs in a cell, not all 45S rDNA copies are transcribed, and extra copies are silenced by repressive chromatin modifications [10,17,20].

The current Col-0 reference genome assembly based on short reads only contains single rDNA units at the top of chromosomes 2 and 4 [21]. None of the recent assembly approaches performed either with Oxford Nanopore Technologies or PacBio provided contigs with multiple 45S rDNA units in tandem [22,23]. Recently, a combination of long- and short-read sequencing using a BAC-based approach built contigs with seven rDNA units, on average, and the first sequence draft of NOR2 [12]. This study confirmed that NOR2 is organized in distinct rDNA unit clusters, as suggested [14], and the fine-tune transcriptional regulation of rDNA units in a tissue-specific manner. These findings support the concept of specialized ribosome subpopulations that differ in their rRNA composition in order to shape the proteome either in a tissue-specific manner or as a response to environmental stresses.

To discriminate rDNA units and guide the assembly of NOR2, barcode systems included (i) the number of core and spacer promoters, (ii) the length and number of SalI boxes, (iii) the presence of an AvaI restriction site in the ITS1 and equal distribution between NORs, (iv) the presence of a specific trinucleotide (CAT) in the ITS2 that strongly enriched on NOR2 and (iv) the length of the 3′ETS [12]. Both 5′ and 3′ETS can also guide the classification of rDNA units within one individual (Figure 1).

Comparison of 5′ETS variants between some Brassicaceae species (*Raphanus sativus*, *Brassica oleracea*, *B. rapa*, *B. juncea* and *Arabidopsis thaliana*) revealed the presence of a 1,1 kbp insertion in *Arabidopsis thaliana* [24,25]. This insertion, made of five D and two C repeats, splits away the conserved U3 snoRNP binding site A^123^B and the primary processing site P [24,25]. Variation at the 5′ETS in the *A. thaliana* Col-0 accession (Figure 1) distinguishes two variants, *VARA* and *VARB*, the first being the major variant [4,9]. Variation at the 3′ETS already distinguishes five variants (*VAR1-5*) based on R1-R5 repeats and indels (Figure 1). Three of these rRNA variants are abundant, *VAR1*, *2* and *3*, accounting for ~50%, ~30% and ~20%, respectively. Meanwhile, *VAR4* is relatively rare (1%), and *VAR5* is undetectable by PCR [9,20,26]. Col-0 is one of the most complex out of the 23 ecotypes studied among the 1135 worldwide accessions, which display only one or two variants [17,26]. Accessions bearing only VAR1 copies, such as Bur-0, are rare worldwide [17,26]. Altogether, these results and observations prompted us to further investigate 45S rDNA organization and, more precisely, variation at the 5′ and 3′ETS in *Arabidopsis thaliana* individuals harvested in the eastern Pyrenees in the Occitanie region of France as elevation ranges from sea level up to almost 3000 m of altitude over distances of less than 100 km, displaying a succession of diverse Mediterranean and mountainous habitats. We found major 45S rDNA variations that can be correlated with geographical distribution.

## 2. Results

### 2.1. The At66 Metapopulation

**Figure 2:**
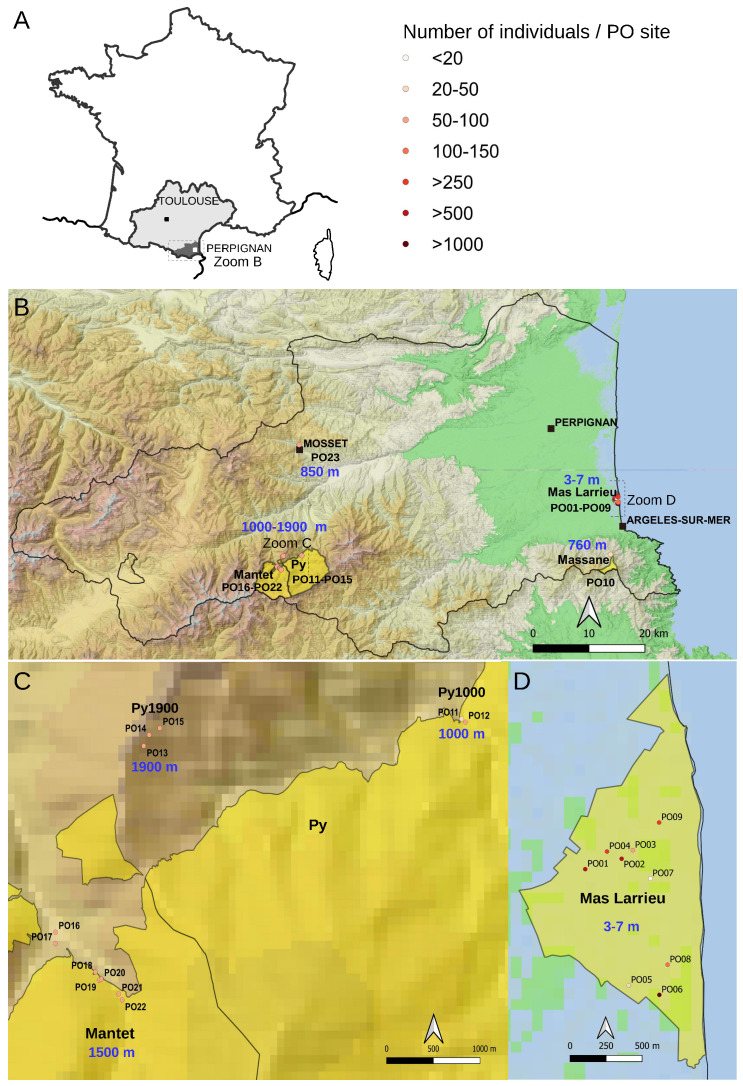
**Map of the At66 metapopulation.** (**A**) Localization of the Pyrénées-Orientales department (PO and 66) in the Occitanie region of France where the 23 sites, namely PO01-23, were sampled. The Perpignan prefecture is located 200 km from Toulouse. The dotted rectangle indicates the area of magnification shown in (**B**). (**B**) Magnification of the department showing the localization of National Nature Reserve (NNR) in yellow and the focus on two localities, PO10 (Massane) and PO23 (Mosset). The border of the Pyrénées-Orientales department (PO and 66) is drawn with a black line. The altitude of each site is indicated in blue. The dotted rectangles indicate the areas of magnification in the mountains (**C**) and at sea level (**D**). (**C**) Magnification of the National Nature Reserve of Py and Mantet in the Pyrenees from 1000 to 1900 m of altitude, focusing on three localities: Py1000 (PO11-PO12), Py1900 (PO13-PO15) and Mantet (PO16-PO22). (**D**) Magnification of the National Nature Reserve of Mas Larrieu at sea level (PO01-PO09). The sites were georeferenced using QGIS software 3.10 (https://www.qgis.org/, accessed on 12 July 2023), including the base map SRTM, © Jet Propulsion Laboratory 2010. The circle symbol, used to georeference the sites, has a color category as a range indicating the number of individuals per PO site. The scale and north orientation of all maps are specified.

The department of Pyrénées-Orientales in the Occitanie region of France has a very interesting topography from the Mediterranean Sea to the Pyrenees, and no natural population of *A. thaliana* was previously sampled there. In this study, we report natural occurrences as a metapopulation named At66 (for *Arabidopsis thaliana* 66, this number being the reference for the Pyrénées-Orientales department (PO) in the French administrative system). At66 ranges from 0 to 1900 m of altitude at a micro-geographic scale as the sites are only distant by several hundreds of meters to less than one hundred kilometers. At66 combines a diversity of habitats and environmental conditions and completes the existing natural populations of the Pyrenees in France [27,28,29] and across the border between France and Spain [30,31,32]. At66 consists of a total of 320 individuals harvested from six localities (Mas Larrieu, Massane, Mosset, Py1000, Mantet and Py1900) subdivided into 23 sampling sites (named PO01-23) (Figure 2). The number of sites differs between localities as there is either only one site (Massane and Mosset) or several sites (nine in Mas Larrieu, two at Py1000, seven in Mantet, three at Py1900) per locality. This study took advantage of this new resource *in natura* to genotype copy number variation and variation at the 5′ and 3′ETS of 45S rDNA units.

### 2.2. Copy Number Variation in At66

**Figure 3:**
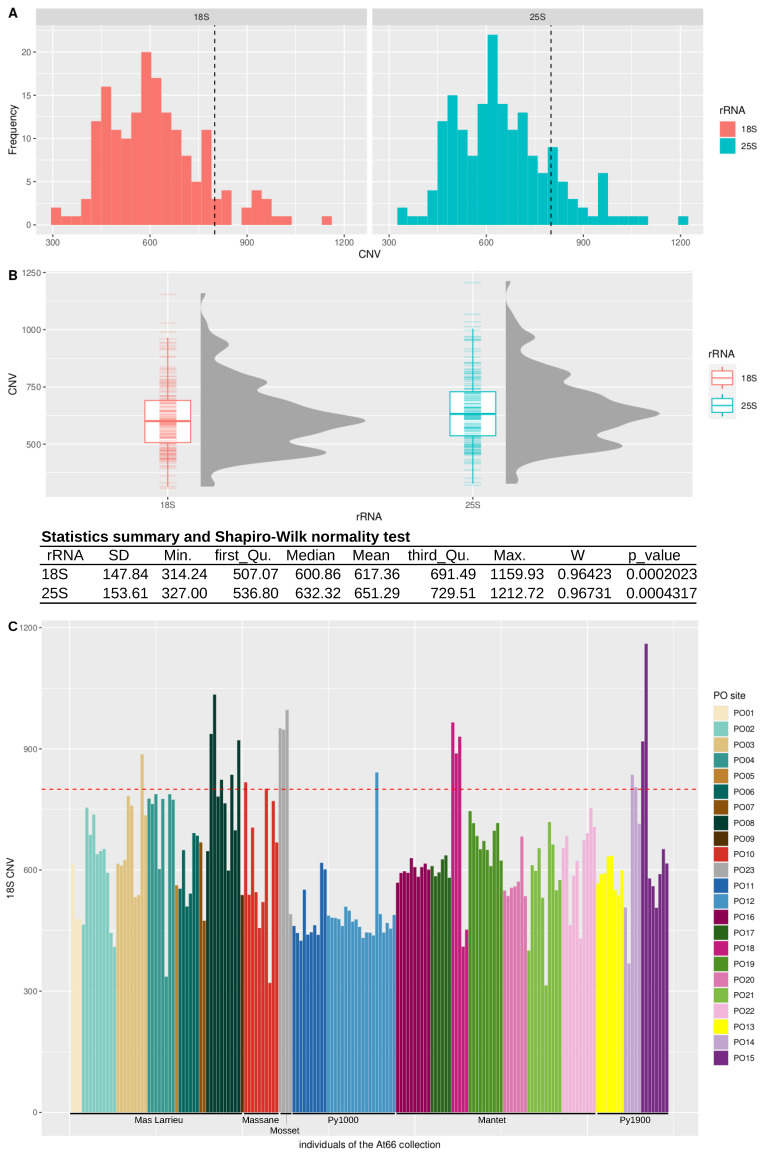
**Copy number variation in the At66 metapopulation.** (**A**) Frequency histogram for both 18S and 25S CNV estimations. The vertical dashed line indicates the number of copies already known for Col-0 (~800). (**B**) Box and violin plots for both 18S and 25S CNV estimations. Both statistic summaries of the data distributions and results of the Shapiro-Wilk normality test are indicated in the table. The *p*-value is lower than 5%, so we can reject the null hypothesis of the normality of the dataset. (**C**) 18S copy number variation in At66. Each bar of the bar plot represents one individual of At66, and the color code indicates the PO site of origin. Individuals are ordered along the altitudinal gradient from sea level to altitude, and the six localities are labeled below the x axis. The horizontal red dashed line indicates the number of copies already known for Col-0 (~800).

The Col-0 reference genome of *Arabidopsis thaliana* counts ~800 copies of 45S rDNA loci per haploid genome [12,13,14]. The genome size of worldwide accessions varies by well over 10% [19,33], and copy number variation in 45S rRNA genes has been identified as one of the contributors to this genome size variation [18,19,34,35]. Copy number variation *in natura* was only reported in Sweden, with northern accessions having a particularly large number of copies (~2500 copies for TRÄ-01) compared to southern accessions (~500 for Ale-Stenar-64-24(1002) [18,19]. We calculated rDNA copy number variation (CNV) in At66, estimating the mean read depth of both 18S and 25S rRNA genes divided by the estimate of the mean genome-wide read-depth, selecting the first 10 Mb of chromosome 3 as it displayed a uniform read-depth [11]. Histograms illustrate the frequency distribution of both 18S and 25S CNVs (Figure 3A), whereas box and violin plots depict distributions associated with a statistics summary (Figure 3B and Table S1). A Wilcox Test confirmed a true shift between the two distributions (W = 12,876, *p*-value = 0.0248), and we think that this bias is probably related to the coverage method of the estimation of CNVs knowing that the 25S rRNA gene is longer than the 18S rRNA gene. We found an average of ~600 copies and registered only a few extreme individuals having either a small number of copies (~300) or doubling the average number of copies (~1200). Interestingly, we registered intra-site and inter-site copy number variation but no significant variation along the altitudinal gradient (Figure 3C and Figure S1). This result suggests that individuals can deal with different numbers of rDNA units to face contrasting environmental conditions and that copy number variation can also distinguish individuals living in the same environmental conditions.

### 2.3. Variation at the 3′ETS in At66

**Figure 4:**
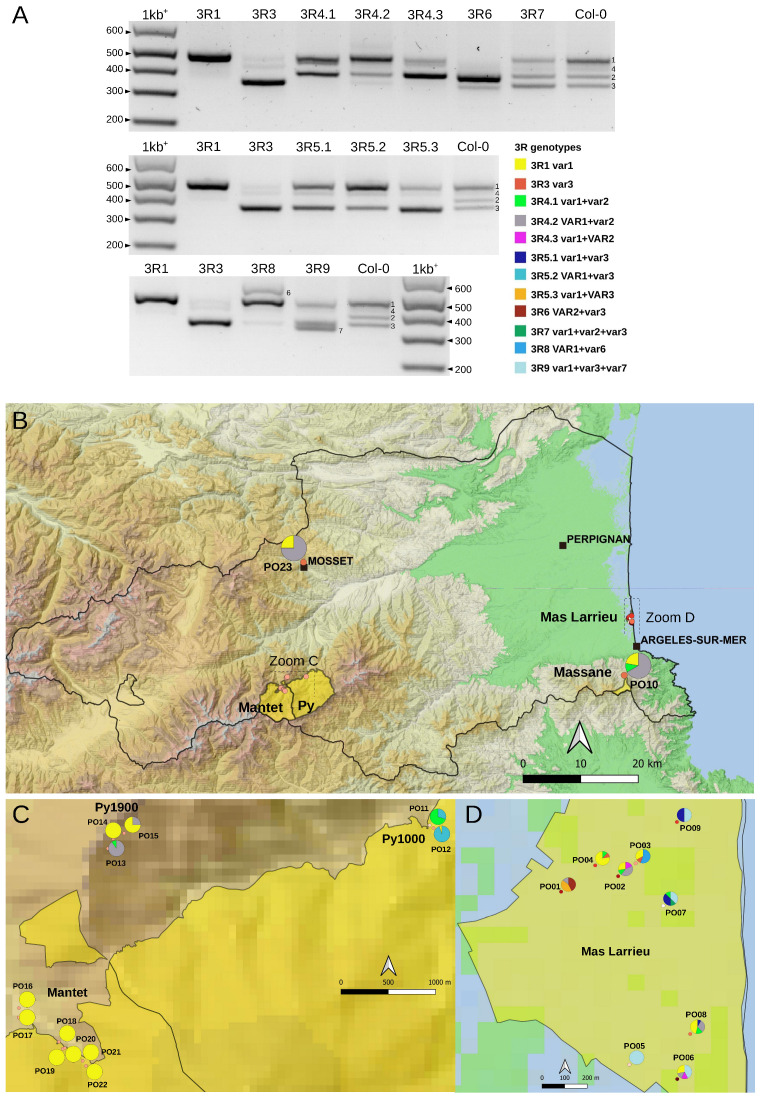
**3′ETS variation in the At66 metapopulation.** (**A**) PCR genotyping illustrating the twelve 3R genotypes identified among individuals of At66. There are three panels (high, medium and low), and each panel includes single-variant genotypes (3R1 and 3R3 in the first two lanes) and Col-0 (last lane) as controls to ensure the accuracy of variant assignment. “3R” is a shortcut for “3′ETS 45S rDNA”. The capital VAR letters versus the lowercase var letters in the 3R genotypes indicate which variant is the most abundant. When both variants have lowercase letters, this means that they are equally abundant. Lowercase var letters are also used when there is only one variant present. Sizes in base pairs are labeled alongside the lane of the 1kb^+^ DNA ladder. (**B**–**D**) Map of 3′ETS variation in At66 metapopulation and pie charts recording the proportion of each 3R genotype in each PO site. (**B**) The magnification of the Pyrénées-Orientales department shows the results of PO10 (National Nature Reserve of Massane) and PO23 (Mosset). The dotted rectangles indicate the areas of magnification in the mountains (**C**) and at sea level (**D**). (**C**) Magnification of the National Nature Reserve of Py and Mantet in the Pyrenees from 1000 to 1900 m of altitude (PO11-22). (**D**) Magnification of the National Nature Reserve of Mas Larrieu at sea level (PO01-PO09). The sites were georeferenced using QGIS software 3.28 (https://www.qgis.org/, accessed on 21 October 2022) and including the base map SRTM, © Jet Propulsion Laboratory 2010. The color categories for each 3R genotype are indicated. The scale and north orientation of all maps are specified.

Small insertions or deletions in the 3′ETS distinguish up to five 45S rDNA variants (*VAR1-5)* in the *A. thaliana* Col-0 accession [9,20,26]. Four of them (*VAR1-4*) are detectable by PCR (Figure 1 and Figure 4A, see Col-0 control lane), and variation at the 3′ETS was previously used to show that nine natural Pyrenean populations were homogeneous [28]. Here, we studied natural variation at the 3′ETS in At66 and identified 12 different 45S rDNA genotypes at the 3′ETS (3R) among 320 individuals (Figure 4A). *VAR4* was not taken into account to define 3R genotypes due to its variability and low frequency. We subdivided 3R4 and 3R5 into three distinct 3R genotypes, 3R4.1-3 and 3R5.1-3, considering the relative abundance of *VAR1-3* copies. For example, we distinguished 3R4.1, 3R4.2 and 3R4.3 to indicate that the abundance of *VAR1* and *VAR2* were equivalent in 3R4.1 and that *VAR1* or *VAR2* were the most abundant in 3R4.2 or 3R4.3, respectively. We identified individuals bearing either only *VAR1* or *VAR3* copies but not only *VAR2* copies (no potential 3R2 genotype). Nearly half of the individuals of At66 (48.1%) have the 3R1 genotype with only *VAR1* copies. The other 3R genotypes, being in the majority, are 3R5.2 (17.2%) and 3R4.2 (15.3%). The minor represented 3R genotypes are 3R4.1 (5.9%), 3R9 (4.7%), 3R4.3 (2.5%), 3R5.1 (1.9%) and 3R8 (1.6%). The 3R3, 3R5.3, 3R6 and 3R7 genotypes are less represented and considered rare (<1%). Pie charts illustrate the proportion of these 3R genotypes for each site georeferenced on the map of the department of Pyrénées-Orientales (Figure 4B–D and Table S2). There is nearly three times more 3R genotypes diversity at sea level (eleven in Mas Larrieu, and only R5.2 is missing out of twelve genotypes) than at altitude (only four in Massane, Mosset, Py and Mantet). The 3R1 genotype, with *VAR1* copies only and present in 154 among 320 individuals, was found all along the altitudinal gradient (0–1900 m). Interestingly, we identified two new 3′ETS rDNA variants in the 3R8 (*VAR6*) and 3R9 (*VAR7*) genotypes (Figure 4A).

**Figure 5:**
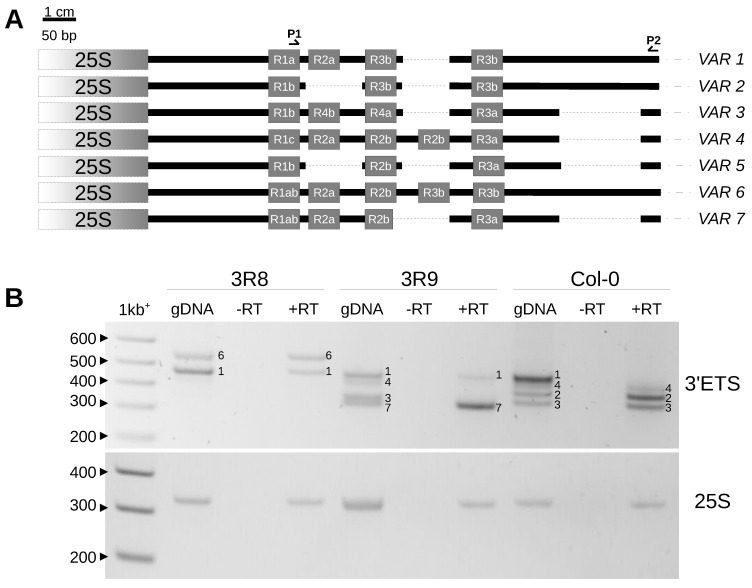
**New 3′ETS variants identified in the At66 metapopulation.** (**A**) Structure of new *VAR6* and *VAR7* variants compared to known *VAR1-5* variants described in Col-0. (**B**) RT-PCR analysis carried out for 3R8 and 3R9 genotypes harboring new *VAR6* and *VAR7* variants compared to *VAR1-4* variants known in Col-0. gDNA corresponds to the 3′ETS genotyping, -RT is the control proving that there are no genomic DNA contaminants left in the total RNA sample before reverse transcription, and +RT is the cDNA sample. RT-PCR amplification was performed for 3′ETS and 25S rRNA genes as an internal control of equal cDNA loading. Sizes in base pairs are labeled alongside the lane of the 1kb^+^ DNA ladder.

Compared to already known *VAR1-5*, *VAR6* is the longest variant, with five R repeats like *VAR4* but not the deletion shared by *VAR3-5* and *VAR7*. Meanwhile, *VAR7* is similar to *VAR3* with an additional deletion right after the R2b repeat, making it slightly smaller than *VAR3* (schematic representation of 3′ETS variants in Figure 5A, FASTA sequences and multiple alignments in Figure S2). Since only *VAR2-4* copies are expressed in adult *A. thaliana* Col-0 plants, whereas *VAR1* is silenced [20,36], we investigated whether the newly identified *VAR6-7* variants could contribute to the functional subset of 45S rDNA copies (Figure 5B). Interestingly, in the 3R8 genotype (Figure 4A), *VAR6* is much less abundant compared to *VAR1*; however, a similar accumulation of *VAR1* and *VAR6* transcripts is detected (Figure 5B). In contrast, in the 3R9 genotype with a similar abundance of rDNA *VAR1, 3* and *7* (Figure 4A), mainly transcripts corresponding to *VAR7* are detected (Figure 5B).

Altogether, this analysis reports the geographical distribution of 12 different 3′ETS 45S rDNA genotypes containing previously reported *VAR1-4* and/or novel *VAR6-7* variants that are transcriptionally active. The diversity of genotypes in At66 is three times higher at sea level compared to altitude (760–1900 m). Individuals bearing only *VAR1* copies are more frequent than expected from worldwide studies, as we reported a 50/50 chance of receiving this genotype in At66 all along the altitudinal gradient. This result suggests that individuals can cope with little rDNA diversity at the 3′ETS to face contrasting environmental conditions.

### 2.4. Variation at the 5′ETS in At66

**Figure 6:**
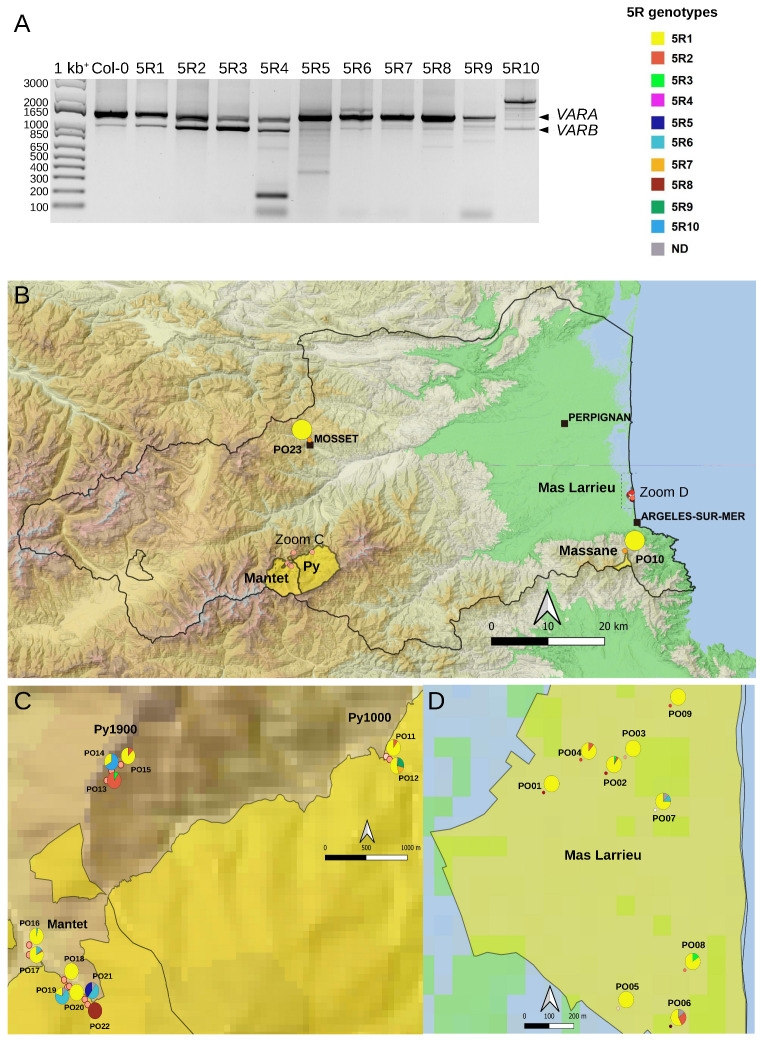
**5′ETS variation in the At66 metapopulation.** (**A**) PCR genotyping illustrating the ten 5R genotypes identified among individuals of At66. Only one 5′ETS genotype was reported for Col-0 with two variants *VARA* and *VARB*, *VARA* being the major one (lane 1) and renamed here 5R1 (lane 2). We identified nine additional 5R genotypes named 5R2-R10 (lanes 3–11), “5R” being a shortcut for “5′ETS 45S rDNA”. Sizes in base pairs are labeled alongside the lane of the 1kb^+^ DNA ladder. (**B**–**D**) Map of 5′ETS variation in the At66 metapopulation and pie charts recording the proportion of each 5R genotype in each PO site. (**B**) The magnification of the Pyrénées-Orientales department shows the results of PO10 (National Nature Reserve of Massane) and PO23 (Mosset). The dotted rectangles indicate the areas of magnification in the mountains (**C**), with the National Nature Reserve of Py and Mantet in the Pyrenees from 1000 to 1900 m of altitude (PO11-22) and at sea level (**D**) with the National Nature Reserve of Mas Larrieu (PO01-PO09). The sites were georeferenced using QGIS software 3.10 (https://www.qgis.org/, accessed on 12 July 2023) and including the base map SRTM, © Jet Propulsion Laboratory 2010. The color categories for each 5R genotype are indicated. The scale and north orientation of all maps are specified.

Processing of the 45S pre-rRNA is a keystone of ribosome biogenesis, and its initial endonucleolytic cleavage in *A. thaliana* is located at the P site in the 5′ETS (Figure 1) [25]. Since the primary cleavage at the P site is impaired upon heat stress, highlighting a functional role of the 5′ETS [37], we decided to study natural variation at the 5′ETS in At66 (Figure 6A–D). Only one 5′ETS genotype was reported to date in *A. thaliana* with two variants, *VARA* and *VARB*, *VARA* being the major variant (Figure 1) [4,9]. The majority of the individuals (71.6%) have only this genotype recorded so far in Col-0 and renamed here 5R1 (Figure 6A and Table S2). However, one-third of the individuals of At66 have a distinct 5′ETS, and we identified nine additional genotypes at the 5′ETS (5R2-10) (Figure 6A and Table S2), eight of which are minorly represented (1.3–5.6%) and one is considered rare (<1%). *VARA* is often the major variant but can also be as equally present as *VARB* (5R2, 5R4) or rare (5R10). *VARB* can be the major variant (5R3). Noteworthily, we identified only one individual among 320 having a 200 bp variant equally present as both *VARA* and *VARB* (5R4). We also identified intermediate or longer variants (5R4–5R9), suggesting that length variation of the 5′ETS could be affected between individuals and within rDNA copies of one individual. Pie charts illustrate the proportion of these 5R genotypes for each site georeferenced on the map of the department of the Pyrénées-Orientales (Figure 6B–D and Table S2). There is 2.5 times more 5R genotypes diversity at altitude (ten in Massane, Mosset, Py and Mantet) than at sea level (four in Mas Larrieu). The 5R1 genotype bearing *VARA* and *VARB*, *VARA* being the major variant and present in 229 among 320 individuals, was found all along the altitudinal gradient (0–1900 m).

Altogether, this analysis reports the geographical distribution of 10 different 5′ETS 45S rDNA genotypes, confirming that the only previously reported 5R1 genotype is present in the majority, as we reported a 70/30 chance of receiving this genotype in At66 all along the altitudinal gradient; however, it still identifies nine additional genotypes sustaining ~30% of 5′ETS variation that is unknown so far. This result suggests that individuals can cope with little rDNA diversity at the 5′ETS to face contrasting environmental conditions.

### 2.5. 5′ETS Length Variation in Brassicaceae Species

**Figure 7:**
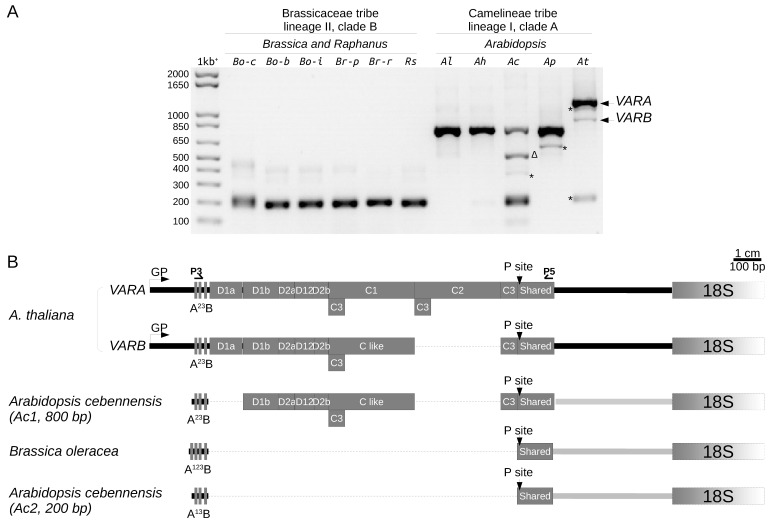
**5′ETS variation in Brassicaceae species.** (**A**) 5′ETS genotyping in the genus *Arabidopsis* (Camelineae tribe, lineage I, clade A) compared to *Brassica* and *Raphanus* (Brassicaceae tribe, lineage II, clade B). Bo-c, *Brassica oleracea* var *capitata*; Bo-b, *Brassica oleracea* var *botrytis*; Bo-i, *Brassica oleracea* var *italica*; Br-p, *Brassica* rapa ssp *pekinensis*; Br-r, *Brassica rapa ssp rapa*; Rs, *Raphanus sativus*; Al, *Arabidopsis lyrata*; Ah, *Arabidopsis halleri*; Ac, *Arabidopsis cebennensis*; Ap, *Arabidopsis pedemontana*; At, *Arabidopsis thaliana*. * Uncloned, ∆, aspecific amplified PCR products. Sizes in base pairs are labeled alongside the lane of the 1kb^+^ DNA ladder. (**B**) Schematic representation of *A. thaliana* Col-0 5′ETS variants (*VARA* and *VARB*) compared to the 200 bp variant of *Brassica oleracea* without the 1.1 kbp insertion made of five D and three C repeats; splitting away the conserved U3 snoRNP binding site A^123^B from the primary processing site *P. Arabidopsis cebennensis* has both a ~800 bp variant similar to the *A. thaliana* VARB without the D1a repeat and the 200 bp insertion-free variant like *Brassica* species. Dotted lines indicate deletions.

Since we identified length variation of the 5′ETS between At66 individuals and within rDNA copies of a single individual in At66, we further investigated 5′ETS structural variation in Brassicaceae species. The 5′ETS of the *Arabidopsis thaliana* Col-0 accession is longer compared to Brassicaceae species (*Raphanus sativus*, *Brassica oleracea*, *B. rapa* and *B. juncea*) because a 1.1 kbp insertion made of five D and two C repeats splits away the conserved U3 snoRNP binding site A^123^B and primary processing site P [24,25]. We wondered whether this 1.1 kbp insertion was conserved in the *Arabidopsis* genus, and to answer this question, we combined both 5′ETS genotyping (Figure 7A) and the sequencing of 1–3 independent clones per major variant detected (schematic representation of 5′ETS variants in Figure 7B and FASTA sequences and multiple alignments in Figure S3).

5′ETS PCR genotyping of some species belonging to the Brassicaceae tribe, lineage II, clade B (*Brassica oleracea* var *capitata*, *botrytis* and *italica*, *Brassica rapa* ssp *pekinensis* and *rapa* and *Raphanus sativus*) identified a 200 bp variant (Figure 7A), confirming the absence of the 1.1 kbp insertion [24,25]. In contrast, the *Arabidopsis thaliana* Col-0 has two longer variants (Figure 7A,B), *VARA* and *VARB* of 1260 and 950 kbp, respectively, *VARA* being the major variant [4,9]. *VARA* and *VARB* share the same five D repeats (D1a, D1b, D2a, D12 and D2b) but differ from each other by the deletion of one C repeat of 310 bp long [4,9]. The C repeat left in VARB was designed C-like as it likely results from a combination of VARA C1 and C2 repeats rather than the selective deletion of either C1 or C2 [9]. All C-repeats (C1, C2 and C-like) begin by a 59 bp C3 repeat [4]. There is an additional third occurrence of the C3 repeat following the C2 or C-like repeat [4]. An additional minor variant slightly smaller than *VARA* was frequently detected while genotyping *A. thaliana* (Figure 7A) but we could not obtain any clone for identification.

5′ETS PCR genotyping in the *Arabidopsis* genus (Camelinae tribe, lineage I, clade A) identified one variant of ~800 bp present in all species (*A. lyrata*, *halleri*, *cebennensis* and *pedemontana*) and a 200 bp variant only identified in *A. cebennensis* and *A. thaliana* (Figure 7A). We were unsuccessful in cloning minor intermediate variants visible in *A. cebennensis* and *A. pedemontana* but found that the major intermediate variant of 550 bp in *A. cebennensis* resulted from an aspecific amplification (Figure 7A). Interestingly, genotyping *A. cebennensis* revealed that a ~800 bp variant similar to the *A. thaliana VARB* without the D1a repeat and a 200 bp variant without an insertion like in *Brassica* species can co-exist within a unique individual (Figure 7B). The minor 200 bp variant found in the *A. thaliana* Col-0 accession was only seen once, and we were not successful in amplifying it again while genotyping hundreds of other Col-0 plants. This result suggests that rDNA units without the 1.1 kbp insertion might also exist in *A. thaliana* but they are rare and frequently below the threshold of detection by genotyping. Noteworthily, only one individual among 320 (5R4 genotype) in the At66 metapopulation has such a 200 bp insertion-free variant equally present as both *VARA* and *VARB* (Figure 6A).

### 2.6. 45S rRNA Gene Polymorphisms in At66

**Figure 8:**
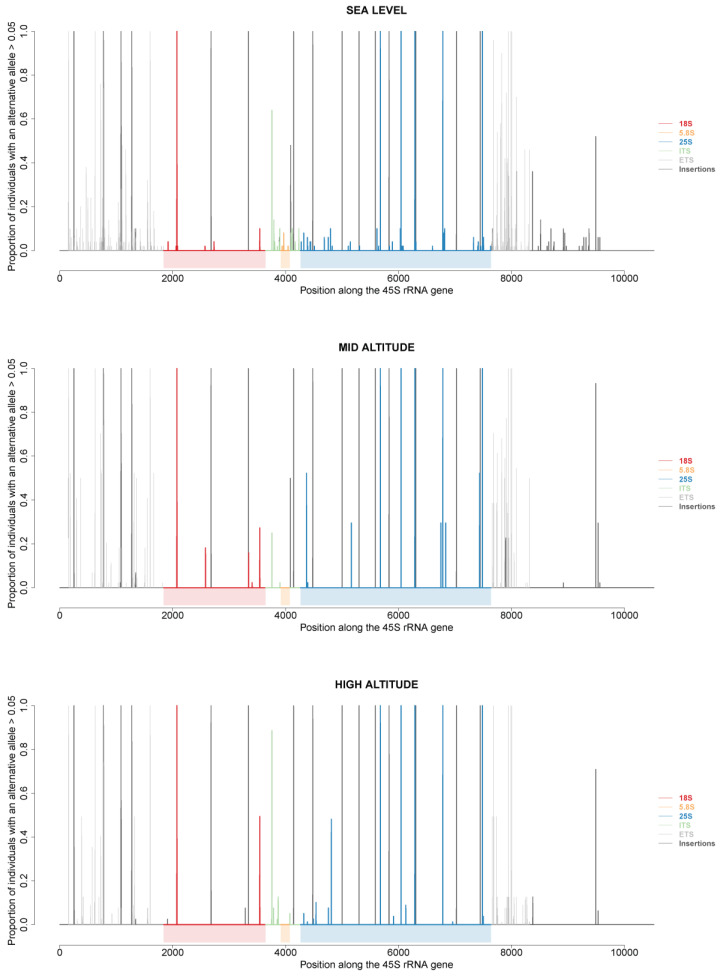
**45S rRNA gene polymorphisms at sea level and mid and high altitude.** Proportion of individuals of At66 with an alternative allele (defined as >5% within an individual) along the 45S rRNA gene. Vertical lines represent single nucleotide polymorphisms or deletions in the ETS (gray), 18S (red), ITS (green), 5.8S (orange) and 25S (blue) regions along the 45S rRNA gene. Black vertical lines represent insertions.

Variations at the 5′ and 3′ETS are not directly involved in ribosome heterogeneity since these sequences are removed during the processing of 45S pre-rRNA into mature 18S, 5.8S and 25S rRNAs. However, they clearly helped distinguish different rDNA units within and between individuals of At66, and we wondered whether these variations might be associated with polymorphisms present in specific 18S, 5.8S and 25S rRNA sequences. Using recently developed bioinformatic methods [11], we retrieved the sum of frequencies of all alternative alleles for each position along a ~10.5 kb 45S rDNA unit for each of 173 among 320 individuals of At66. Then, we plotted the proportion of individuals of At66 with an alternative allele for each position along this rDNA unit by studying three cases: firstly, comparing sea level versus mid and high altitude (Figure 8); secondly, by selecting either the 115 individuals out of 173 sharing the same 5R1 genotype or the 92 individuals out of 173 sharing the same 3R1 genotype (Figure S4A); and thirdly, comparing the six localities (Figure S4B). In the first case studied, true polymorphisms present in 18S, 5.8S and 25S rRNA sequences clearly vary along the altitudinal gradient, and polymorphisms are detected only at sea level for the 5.8S rRNA sequence (Figure 8).

In the second case studied, the proportion of individuals sharing alternate alleles vary when fixing either the 5′ETS (same 5R1 genotype and 3′ETS variation) or the 3′ETS (5′ETS variation but same 3R1 genotype). There are additional alternative alleles when fixing the 5′ETS, minorly in 18S and 5.8S rRNA sequences and mostly in the 25S rRNA sequence (Figure S4A). In the third case studied, there is clearly a variation of true polymorphisms within coding (18S, 5.8S and 25S rRNAs) and noncoding ITS sequences between localities. Polymorphisms within the 5.8S rRNA sequence are only detected at Mas Larrieu (sea level). Polymorphisms detected within 18S and 25S rRNA sequences are either specific to one locality (e.g., Massane and 25S) or the proportion of individuals sharing an alternate allele vary between localities (Figure S4B). The most polymorphisms within ITS1 and 2 are detected at Mas Larrieu (sea level) and Mantet (1500 m), whereas there are none at Massane or only a few ITS1 polymorphisms at Mosset, Py1000 and Py1900. Altogether, true SNP polymorphisms detected within coding rRNA sequences in individuals of the At66 metapopulation vary along the altitudinal gradient, between localities and probably depending on variants present at the 5′ and 3′ETS.

## 3. Discussion

*In natura*, the number of 45S rDNA loci was previously reported in the range of 500–2500 in Swedish accessions [18,19], and we reported here a 300–1200 copy number variation in At66. We registered both intra-site and inter-site copy number variation along a sharp altitudinal gradient (0–1900 m) and detected 2–2.5-fold changes between individuals within the same site. Interestingly, ten individuals harvested in the PO10 site (Massane locality), namely PO10A–J, are in the range of 320–820 copies, with three individuals having either the upper (PO10H) or the lower number of copies (PO10A and G) and seven individuals in the range of an intermediate number of copies. The estimated kinship coefficient between each pair of these individuals is 0.4, suggesting that this copy number variation is observed among offspring. Moreover, among ten individuals harvested in the PO12 site (Py1000 locality), only one individual has 840 copies (PO12O and the least related one with a negative estimated kinship coefficient in pair comparisons), while the remaining nineteen individuals have 430–510 copies. These two concrete examples from this study suggest that rDNA copy number variation is very dynamic *in natura* and apparently independent of environmental conditions. The first attempt to map copy number variation as a phenotype by means of genome-wide association studies (GWAS) using Swedish accessions did not identify either of the two NORs in a cis-acting locus at chromosome 1 but did in a trans-acting one [19]. Our attempt to map the variation in At66 by means of GWAS conducted by GAPIT version 3 [38] using BLINK [39] or FarmCPU [40] multi-locus models only identified a few SNP outliers shared by both models and both 18S and 25S CNVs, but still apparently not relevant either in cis or trans (Figure S5). Thus, it would confirm the previous statement that the trait would behave like a genotype rather than a phenotype. Indeed, mapping 45S rDNA copy number variation using F2 crosses, recombinant inbred lines, the multiparent advanced generation inter-cross population and mutation accumulation lines showed that both NORs vary in size and that rDNA copy number variation can either behave like a genetic trait heritable in pedigrees or like an unstably inherited trait as it starts to diverge over a timescale of tens of generations [18]. There are two potential mechanisms to promote the dynamics of rDNA loci, recombination and transposition [41]. However, intragenomic fluctuation in rDNA copy number would avoid homologous recombination between NORs on distinct chromosomes, favoring the repair of DNA Strand Breaks by non-homologous end joining (NHEJ), maintaining rDNA integrity during meiosis [42,43]. Therefore, as NORs and neighboring regions are frequent targets for transposable element insertions, transposition would be a good driver to sustain any variation [41,44]. A reduction of the number of 45S rDNA units either in loss-of-function mutants [45,46] or CRISPR-Cas9-induced Double Strand Breaks (DSB) at rDNA loci [43,47] demonstrated that rDNA stability is vital for genome stability. Indeed, affecting the integrity of rDNA loci in loss-of-function mutants [46] or CRISPR-Cas9-induced Double Strand Breaks at rDNA loci [47] give rise to genome rearrangements such as tandem duplications in direct orientation [46] or chromosome-segment duplication [47]. CRISPR-Cas9-inducing DSB at rDNA loci can trigger seedling lethality or organ/tissue ablation [43] and compromise both ovule fertilization and embryo development [47,48]. Whether impacts on plant development would be direct consequences of modifying rDNA loci number or indirect consequences modifying the integrity of the genome remains to be unraveled. We reported the lowest number of rDNA copies *in natura* so far (300 copies) but the genome seems well-buffered against an artificial loss of rDNA copies occurring randomly at both NORs. Indeed, as little as 10% of the wild-type 800 copies per haploid genome are sufficient for viability in the *A. thaliana* Col-0 accession [47].

Although rRNA transcripts represent the majority of all transcribed RNAs in a eukaryotic cell, only a fraction of available 45S rDNA loci are transcribed [10,20]. Both NORs are transcribed at the onset of meiosis [42], during embryogenesis and the first few days after germination [20,36,49]. In adult somatic tissues, both NORs can remain active in some accessions whereas either of the two NORs can be silenced in other accessions [10,17]. Therefore, if the quantity/quality of available rDNA loci and contribution of NORs vary between individuals *in natura*, this might sustain the variation of expressed subsets of 45S rDNA units as one potential source of ribosomal heterogeneity [2,3,12].

Variations at the 5′ and 3′ETS are likely not involved in ribosome heterogeneity since these sequences are removed during the processing of 45S pre-rRNA into mature 18S, 5.8S and 25S rRNAs. However, we demonstrated that studying their variation *in natura* contributes to distinguishing rDNA units both within and between individuals. These variations are also associated with polymorphisms present in specific 18S, 5.8S and 25S rRNA sequences and are potentially different depending on altitude or 5′ and 3′ETS variants. Specific nucleotide and/or insertion/deletion polymorphisms in the ETS, ITS and mature rRNA sequences were linked to rDNA variants from active NORs and rRNA assembled into ribosomes [12]. Thus, in a similar way, associating structural variations of pre-rRNA with different 5′ETS and 3′ETS R genotypes should provide information about the impact of these structural variations in controlling the synthesis of rRNAs. Undoubtedly, 5′ETS length might affect the earliest pre-rRNA processing step at the P site and, subsequently, the production of specific rRNAs, while 3′ETS structural variations might affect RNA pol I transcription termination [3].

Our study of the variation at the 3′ETS in 320 individuals of At66 identified 12 different 45S rDNA (R) genotypes and two new expressed variants, *VAR6* and *VAR7*, in addition to already known *VAR1-5* [9,20,26]. Still, the question remains open to know whether the newly expressed *VAR6* and *VAR7* copies are located on the same or different NORs or both NORs. Nearly half of the individuals of At66 have only *VAR1* copies and are present from sea level up to 1900 m of altitude. This raises the question of whether these individuals express one or another subset of their *VAR1* copies depending on their NOR localization.

This study also investigated variation at the 5′ETS *in natura* and demonstrated that the R1 genotype (*VARA* much higher than *VARB*) identified in Col-0 is present in most of the individuals tested in At66 (71.6%), regardless of the 3′ETS. However, this study also identified nine additional R2–10 genotypes, each represented in the order of a small percentage but altogether present in 28.4% of the individuals tested. We do not know if the 45S rDNA genes containing 5′ETS R2–10 sequences are expressed and/or processed into mature rRNAs. This also raises the question of whether rDNA units bearing these variants are expressed or not depending on their localization between or within NORs.

Our investigation of the variation at the 5′ETS in Brassicaceae confirmed a previous result [24,25] showing that the 1.1 kbp insertion made of five D and two C repeats and splitting away the conserved U3 snoRNP binding site A^123^B from the primary processing site P in *A. thaliana* is absent in species belonging to the Brassicaceae tribe, lineage II, clade B. Additionally, 5′ETS genotyping in the Camelinae tribe, lineage I, clade A (*Arabidopsis lyrata, halleri, cebennensis* and *pedemontana*) demonstrated that the length of the 5′ETS rDNA sequence in the *Arabidopsis* genus depends on the number and integrity of D and C repeats. The presence of insertion is not only a feature of the *Arabidopsis* genus but was also identified in other tribes of lineage I, clade A (Figure S6). Interestingly, in these additional studied species (*Boechera stricta, Chrysochamela velutina, Lepidum campestre, Cardamine parviflora, Descurainia millefolia*), the insertion either partially retains some homologies with some D and C repeats and/or interrupts them by insertions or shares no homology with any of them. Altogether, 5′ETS genotyping in Brassicaceae species highlighted that variants with and without an insertion can co-exist within a single individual (*Arabidopsis cebennensis* and *thaliana*, *Boechera stricta, Chrysochamela velutina* and *Lepidum campestre*).

Interestingly in At66, we found the lowest and highest 5′ETS genotype diversity for individuals living, respectively, at sea level and altitude (760–1900 m). Surprisingly, the exact opposite is true for 3′ETS genotype diversity (i.e., highest at sea level and lowest in altitude). In this context, we wondered whether altitude or bioclimatic variables could explain part of the 45S rDNA copy number variation in At66. We standardized the variance for a selection of least correlated variables (altitude and WorldClim bio3, bio4, bio8, bio9, bio13 and bio15) and used a model-averaging approach to answer this question. The best model provided with an R2 = 0.22 for 18S CNV did not support any effect as the confidence interval was overlapping zero with *p*-value > 0.05 for all variables tested (Figure S7) [50,51,52,53,54,55,56]. Although there is apparently no correlation between global environmental conditions and copy number variation along a sharp altitudinal gradient, qualitative variation registered for rDNA copies at sea level versus altitude could promote a better response of individuals to local environmental stresses.

At66 is a worthwhile natural laboratory, and the unraveled 45S rDNA diversity represents an interesting starting material to select subsets for rDNA transcription and alter the rRNA composition of ribosomes both intra- and inter-site. It would be interesting to assign rDNA copies and their variants to their NORs of origin as these variants are also reporters of rDNA-cluster-specific expression, but this would only be feasible in recombinant inbred or F2 lines [10,11]. As rDNA stability is vital for genome integrity, it would also be interesting to investigate whether the gain or loss of rDNA copies is associated with polymorphisms in TE insertions or other genome structural variants. Ribosome heterogeneity not only includes the sequence variation of rRNA but also the variation of ribosomal proteins (RP) from the canonical structure, exchange of RP paralogs and post-transcriptional and post-translational modifications of rRNAs or RPs [2,57]. Therefore, plant ribosomes are heterogeneous at multiple levels, and there is still much that remains to be understood regarding the functional consequences of ribosome heterogeneity.

## 4. Materials and Methods

### 4.1. Sampling Individuals of At66 and Growth Conditions

The sampling campaign of 320 individuals of At66 was performed during two consecutive years, 2015–2016, from March to the end of June, depending on the altitude. Sampling was performed in priority within or near National Nature Reserves in collaboration with FRNC (Fédération des Réserves Naturelles Catalanes, Prades, France) to ensure that access to the material would be reliable over time. For all sampling performed *in natural* reserves, special authorizations were granted by the ”Direction Départementale des Territoires et de la Mer 66” (DDTM 66). Each individual was collected as an adult plant with matured siliques, haphazardly chosen between multiple patches with a minimal distance of approximately 1 m between individuals to fulfill seed dispersal requirements [58]. Sampling efforts (4–40 individuals) depended on the number of individuals per site and number of patches in order to include the maximum diversity present at a site. Within the same locality, we considered that we had a distinct site any time the habitat changed or when we did not find any individual within a few hundred meters. Field-collected seeds for each individual were reproduced one time in a growth chamber to obtain a batch of seeds with reduced maternal effects on trait expression. Lineages were established by selfing to recover single-seed descendants sharing the same controlled maternal environment. Individuals were named using the two letters PO (for Pyrénées-Orientales) followed by the number of the site (01–23) and one letter (A–Z) for their identification. The sites were georeferenced using QGIS software 3.28 (https://www.qgis.org/, accessed on 21 October 2022), including the base map SRTM, © Jet Propulsion Laboratory 2010, was used to georeference the 23 sites of At66.

All field-coming seeds were surface-sterilized by constant agitation with 70% (*v*/*v*) ethanol for 10 min, then washed with 95% (*v*/*v*) ethanol and dried. Seeds were placed on plates containing 50 mL of Murashige and Skoog medium with 0.5 g L^−1^ MES and 0.8% (*w*/*v*) plant agar (M0231; Duchefa) with 1% (*w*/*v*) sucrose. All plates were first stratified in darkness at 4 °C for 6 days and then incubated for 2 weeks at 20 °C with 160 µE m^−2^ s^−1^ light intensity and a 16 h light/8 h dark regimen. Seedings were transplanted in a mixture of soil and vermiculite (3:1, *v*/*v*), irrigated with water and grown at 22 °C with 70% hygrometry under a 16 h light (200 μE)/8 h dark regimen. Depending on the altitudinal origin of individuals, rosette leaves required vernalization for 4 weeks at 4 °C to induce flowering.

### 4.2. Additionnal Plant Material

Seeds from other species of the *Arabidopsis* genus (*lyrata, halleri, cebennensis* and *pedemontana*) were gently supplied either by Craig Pikaard (Department of Biology, Department of Molecular and Cellular Biochemistry, and Howard Hughes Medical Institute, Indiana University, Bloomington, Indiana 47405) or Julie Jacquemin (Institute of Plant Breeding, Seed Science and Population Genetics, University of Hohenheim, Stuttgart, Germany) and grown as described above for *Arabidopsis thaliana*.

### 4.3. Estimating rDNA Copy Number Variation and Detection of rDNA Variants through NGS

A subset of 173 individuals of At66 was selected to perform a whole-genome shotgun sequencing (WGS) subcontracted to Novogene Europe, Cambridge, UK. We used an adapted CTAB method using Carlson buffer [59] to extract genomic DNA from pooled leaf tissue harvested from about 25–50 two-week-old sister plants for each individual single-seed descent lineage. The whole genomic library was prepared and sequenced in paired-end mode (2 × 150 bp, insert size: 350 bp) by Novogene Europe, Cambridge, UK using 1 μg of genomic DNA as input material for library preparation. Sequencing libraries were generated using a NEBNext^®^ DNA Library Prep Kit following the manufacturer’s recommendation, and indices were added to each sample. Genomic DNA was randomly fragmented to a size of 350 bp by shearing. DNA fragments were end-polished, A-tailed, ligated with NEBNext adapter for Illumina sequencing and further PCR-enriched by P5 and indexed P7 oligos. The PCR products were purified with AMPure XP system and the resulting libraries were analyzed for size distribution by an Agilent 2100 Bioanalyzer and quantified using real-time PCR. Qualified libraries were fed into unspecified Illumina sequencers. The total output of raw data received on 29 September 2019 for 173 individuals of At66 was 585.0 GB (99.65% clean reads), with an average error rate of 0.03%, a QPHRED20 of 97% and 37% GC content. Raw reads were submitted to the European Nucleotide Archive (ENA) with the BioProject accession number PRJEB48282 and the SRA study accession number ERP132629.

FASTAQ raw reads were trimmed to remove adapters and poor-quality sequences using Trimmomatic V0.36 paired-end mode and the following trimming steps: ILLUMINACLIP, LEADING, TRAILING AND MINLEN [60]. Duplicated reads were removed using a homemade script (available upon request) in order to improve the yield of recovered sequences compared to SAMtools V0.1.9 RMDUP. Cleaned reads were aligned both to the TAIR-10 Col-0 reference genome and a 45S rDNA reference using Bowtie2 V0.2.3.4 [61]. SAMtools V0.1.9 SORT was used to map the reads by position on the chromosomes [62,63]. The 45S rDNA reference includes the most abundant 5′ and 3′ETS variants in Col-0 (*VARA* and *VAR1*), and the annotation of 18S and 25S rRNA genes coordinates, respectively, are 1841-3644 and 4264-7639 (Figure S8). We retrieved per-base read depth for each individual using BEDtools coverage [64] for both 18S and 25S rRNA genes and for the first 10 MB of chromosome 3 that was free of 45S rDNA copies. Copy number variation was estimated by dividing the average coverage along both 18S and 25S rRNA genes by the average coverage along the first 10Mb of chromosome 3 [11]. Using recently developed bioinformatic scripts [11], we retrieved the sum of frequencies of all alternative alleles for each position along our ~10.5 kb 45S rDNA reference for each of 173 among 320 individuals of At66. Then, we plotted the proportion of individuals of At66 with an alternative allele for each position along this rDNA. The alternative variant must be present in at least 5% of the individual’s total 45S rRNA genes (>0.05). All graphs and statistical analyses were performed using the R4.2.2 software [53] in the Rstudio environment [54] and the R package tidyverse [65].

### 4.4. 5′ and 3′ETS Genotyping

5′ and 3′ETS rDNA genotyping was performed on rosette leaves while amplifying each individual for At66 or other species of the *Arabidopsis* genus. Brassicaceae leaves were either bought fresh or supplied gently frozen by Martin Lysak (CEITEC, Masaryk University, Brno, Czechia). Genomic DNA was extracted from three-week-old rosette leaves following the method described by Edwards et al. [66]. PCR amplification of the 5′ETS was performed using the forward primer P1 and the reverse primer P3 using GoTaq^®^ G2 DNA Polymerase (Promega) and the following PCR program design: 40 cycles of 30 s at 94 °C, 45 s at 55 °C and 2 min at 72 °C. PCR products were diluted 1:5 and resolved by electrophoresis on 1% agarose gels in 0.5× TBE buffer. PCR amplification of the 3′ETS was performed using the forward primer P4 and the reverse primer P5 using GoTaq^®^ G2 DNA Polymerase (Promega) and the following PCR program design: 24 cycles of 30 s at 94 °C, 45 s at 55 °C and 45 s at 72 °C. PCR products were diluted 1:5 and resolved by electrophoresis on 1.8% agarose gels in 0.5× TBE buffer running the gel at a maximum of 70 V. A PCR mix control without any DNA template was always included in each PCR amplification to validate the genotypes. When required, PCR amplifications were repeated using the proof-reading DNA polymerase Phusion™ High-Fidelity (FISHER SCIENTIFIC S.A.S., Illkirch Graffenstaden, France), and the PCR products were A-tailed using GoTaq^®^ G2 DNA Polymerase (PROMEGA, Charbonnières-les-bains, France), purified using GeneClean^®^ Turbo for PCR Kit (MP BIOMEDICALS, Illkirch, France) and cloned in the pGEM^®^-T Easy Vector Systems (Promega). Plasmid DNA of 1–3 independent clones per identified band was extracted using Wizard^®^ Plus SV Minipreps DNA Purification Systems (Promega) and sequenced using the Lightrun service of Eurofins GATC BIOTECH (Eurofins GATC BIOTECH—GMBH, Konstanz, Germany). The additional forward primer P2 was used when appropriate for sequencing the longest 5′ETS variants. Sequence data analysis was conducted using Geneious R11 software (https://www.geneious.com, accessed on 12 July 2023), and Multiple Sequence Comparison by Log- Expectation (MUSCLE) multiple alignments were performed using either Geneious or Seaview5 [67]. The QGIS software 3.10 (https://www.qgis.org/, accessed on 12 July 2023), including the base map SRTM, © Jet Propulsion Laboratory 2010, was used to georeference the At66 populations and to display associated pie charts illustrating the proportion of each 3′ETS or 5′ETS genotypes.

### 4.5. Expression of New VAR6 and VAR7 3′ETS Variants

Total RNA was extracted from three-week-old rosette leaves using the Monarch^®^ Total RNA Miniprep Kit according to the manufacturer’s protocol (NEW ENGLAND Biolabs^®^ Inc., Evry, France). DNase treatment was already included in this kit and no additional treatment was required as PCR amplification confirmed that there were no genomic DNA contaminants left in the total RNA. Reverse transcription was carried out using the Invitrogen™ SuperScript™ IV Reverse Transcriptase (FISHER SCIENTIFIC S.A.S., Illkirch Graffenstaden, France) according to the manufacturer’s protocol using the reverse primer P6 located at the end of the 3′ETS of 45S rRNA. RT-PCR amplification of the 3′ETS was performed using the forward primer P4 and the reverse primer P5 using GoTaq^®^ G2 DNA Polymerase (Promega) and the following PCR program design: 30 cycles of 30 s at 94 °C, 45 s at 55 °C and 45 s at 72 °C. RT-PCR amplification of the 25S rRNA gene was performed using the forward primer P7 and the reverse primer P8 using GoTaq^®^ G2 DNA Polymerase (Promega) and the following PCR program design: 16 cycles of 30 s at 94 °C, 45 s at 55 °C and 45 s at 72 °C. The 25S rRNA gene amplification was used as an internal control within 45 rRNAs for equal cDNA loading. PCR products were diluted 1:10 and resolved by electrophoresis on 1.8% agarose gels in 0.5× TBE buffer running the gel at a maximum of 70 V.

### 4.6. Primer List

P1, 5′-CTTTTCCGGGCACTTTTCCGG-3′
P2, 5′-CTTGCGCACGAAATACCGAG-3′
P3, 5′-GTTCGGCGTATGAGTGGTGATCGG-3′
P4, 5′-GACAGACTTGTCCAAAACGCCCACC-3′
P5, 5′-CTGGTCGAGGAATCCTGGACGATT-3′
P6, 5′-CCTCGGACCCGGTAAAC-3′
P7, 5′-TGTTCACCCACCAATAGGGAA-3′
P8, 5′-GGCGGTCCGAACGACCGTTCCGCC-3′

## Data Availability

Raw reads are available at the European Nucleotide Archive (ENA) with the BioProject accession number PRJEB48282 and the SRA study accession number ERP132629.

## Supplementary Materials

The following supporting information can be downloaded at https://www.mdpi.com/article/10.3390/plants12142722/s1, Figure S1: 25S copy number variation in At66; Figure S2: FASTA sequences and multiple alignment of 3′ETS variants; Figure S3: 5′ETS variants identified in Brassicaceae Lineage I, Clade A and Camelineae and Brassicaceae Lineage II, Clade B; Figure S4: 45S rRNA gene polymorphisms in At66; Figure S5: GWAS analysis using 45S rDNA copy number variation as a phenotype; Figure S6: 5′ETS variation in additional species of Brassicaceae Lineage I, Clade A; Figure S7: Model-averaging approach testing the effects of altitude and bioclimatic variables on 18S CNV; Figure S8: FASTA sequence of the 45S rDNA of 10,524 bp used as a reference to estimate 18S and 25S copy number variation and rDNA variants; Table S1: 45S rDNA copy number variation in At66; Table S2: Variation at the 5′ and 3′ETS in At66.
