# Supplementary material for: 45S rDNA Diversity In Natura as One Step towards Ribosomal Heterogeneity in Arabidopsis thaliana
Source: Plants (Basel). 2023 Jul 21;12(14):2722. doi: 10.3390/plants12142722 (PMC10386311; doi:10.3390/plants12142722)
Supplement: Supplementary file 1 [file plants-12-02722-s001.zip › Figure_S1.pdf]

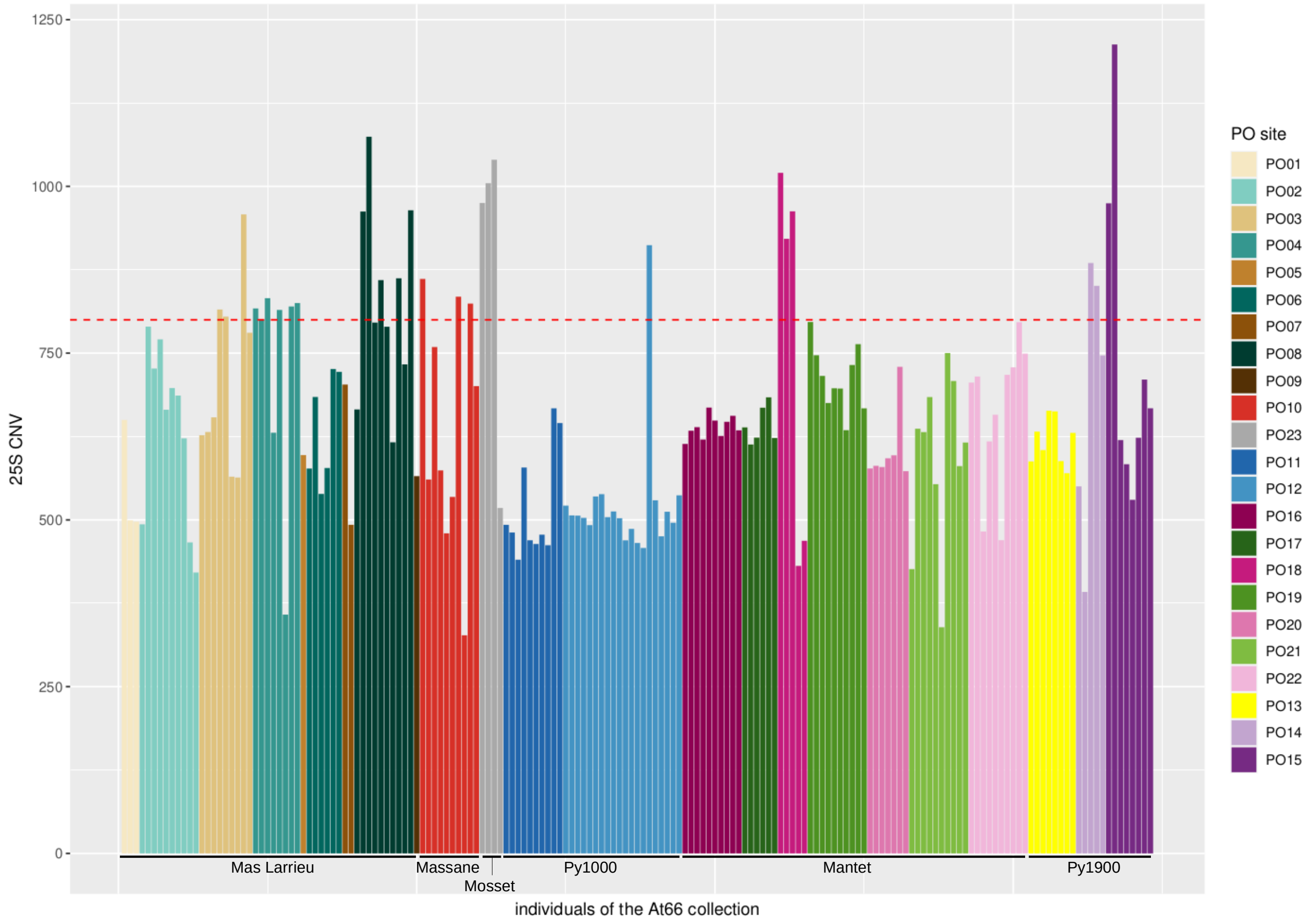

Figure S1: 25S copy number variation in At66. Each bar of the barplot represents one individual of At66 and the color code indicates the PO site of origin. Individuals are ordered along the altitudinal gradient from sea level to altitude and the 6 localities are labelled below the x axis. The horizontal red dashed line indicates the number of copies already known for Col-0 (~800).
