# Supplementary material for: 45S rDNA Diversity In Natura as One Step towards Ribosomal Heterogeneity in Arabidopsis thaliana
Source: Plants (Basel). 2023 Jul 21;12(14):2722. doi: 10.3390/plants12142722 (PMC10386311; doi:10.3390/plants12142722)
Supplement: Supplementary file 1 [file plants-12-02722-s001.zip › Figure_S2.pdf]

Figure S2: Fasta sequences and multiple alignment of 3'ETS variants. **(A)** Fasta sequences of 3'ETS variants. *VAR1* is a consensus sequence of five independent clones from this study and corresponds to *VAR1* type 1 published by Chandrasekhara *et al.* [17]. *VAR2-4* are sequences published by Pontvianne *et al.* [20] while *VAR5* was cloned twice independently in this study. *VAR6* and *VAR7* are new variants identified *in natura* from this study. **(B)** MUltiple Sequence Comparison by Log- Expectation (MUSCLE) alignment of *VAR6* and *VAR7* new variants compared to known *VAR1-5* described in Col-0 [17,20,26]. The color codes for R repeats described in Abou-Ellail *et al.* [26] is the following : R1a, b or c in yellow, R2a in a lighter blue than R2b, R3a in a lighter green than R3b, R4a in a lighter red than R4b.

(A) Fasta sequences of 3'ETS variants.

>*VAR1*
GACAGACTTGTCCAAAACGCCCACCACGAAGGTGCATAGTGAGAAGAGTAAGTCAAGA
GATAGACTTGTCCAAAAAGAAACGGAAGAGAAAGCGTGGGGAGACGCTCACGAAGGT
GCATAGTGAGAAGAGTAAGTCAAGAGACAGACTTGTTCGAAAAGAAACAGAAGAGAA
TGCTTGGGGTTACACTCACGAAGGTGCATAGTGAGAAGAGTAAGTCAAGAGACAGACT
TGTTCGAAAAGAAACAAAAGAGAATGCTTGGGGAGATAGAAGTGTGAGATAGTTCTCA
AGCTAAGAAAGTTGTAAAAGCTAAGAACTAGCATCAAATGATGGATGAAACACAAGGTA
GTTGTTGAAAAGTCAAACACTTGGTGATATGAACACAAACGTTCAATATGACAAACCCA
TGCCAAGTAAAGAGAAAATGAAAACTGGTGATTGTTGCGGAAATCGTCCAGGATTCCTC
GACCAG
>*VAR2*
GACAGACTTGTCCAAAACGCCCACCGCGAAGGTGCATAGTGAGAAGAGTAAGTCAAGA
GACAGACTTGTTCGAAAAGAAACAGAAGAGAATGCTTGGGGTTACACTCACGAAGGTG
CATAGTGAGAAGAGTAAGTCAAGAGACAGACTTGTTCGAAAAGAAACAAAAGAGAAT
GCTTGGGGAGATAGAAGTGTGAGATAGTTCTCAAGCTAAGAAAGTTGTAAAAGCTAAGA
ACTAGCATCAAATGATGGATGAAACACAAGGTAGTTGTTGAGAAGTCAAACACTTGGTG
ATATGAACACAAACGTTCAATATGACAAACCCATGCCAAGTAAAGAGAAAATGAAAACT
GGTGATTGTTGCGGAAATCGTCCAGGATTCCTCGACCAG
>*VAR3*
GACAGACTTGTCCAAAACGCCCACCACGAAGGTGCATAGTGAGAAGAGTAGGTCAAGA
GATAGACTTGTCCAAAAAGAAACGGAAGAGAAAGCTTGGGGAGACGCTCACGAAGGT
GCATAGTGAGAAGAGTAGGTCAAGAGATAGACTTGTTCAAAAAGAAACAGAAGAGAAT
GCTTGGGGTTACACTCACGAAGGTGCATAGTGAGAAGAGTAAGTCAAGAGACAGACTT
GTTCAAAAAGAAACGAAAGAGAATGCTTGGGGAGATAGAAGTGTGAGATAGTTCTCAA
GCTAAGAAAGTTGTAAAAGCTAAGAACTAGCAAGTAATCGTCCAGGATTCCTCCACCAG
>*VAR4*
GACAGACTTGTCCAGAACGCCCACCACGAAGGTGCATAGTGAGAAGAGTAAGTCAAGA
GATAGACTTGTCCAAAAAGAAACGGAAGAGAAAGCTTGGGGAGACGCTCACGAAGGT
GCATAGTGAGAAGAGTAAGTCAAGAGATAGACTTGTTCAAAAAGAAACAGAAGAGAAT
GCTTGGGGTTACACTCACGAAGGTGCATAGTGAGAAGAGTAAGTCAAGAGATAGACTT
GTTCAAAAAGAAACAAAAGAGAATGCTTGGGGTTACACTCACGAAGGTGCATAGTGAG
AAGAGTAAGTCAAGAGACAGACTTGTTCAAAAAGAAACAAAAGAGAATGCTTGGGGA
GATAGAAGTGTGAGATAGTTCTCAAGCTAAGAAAGTTGTAAAAGCTAAGAACTAGCAAG
TAATCGTCCAGGATTCCTTCACCAG
>*VAR5*
GACAGACTTGTCCAAAACGCCCACCGCGAAGGTGCATAGTGAGAAGAGTAAGTCAAGA
GATAGACTTGTTCAAAAAGAAACAGAAGAGAATGCTTGGGGTTACACTCACGAAGGTG
CATAGTGAGAAGAGTAAGTCAAGAGACAGACTTGTTCAAAAAGAAACAAAAGAGAAT
GCTTGGGGAGATAGAAGTGTGAGATAGTTCTCAAGCTAAGAAAGTTGTAAAAGCTAAGA
ACTAGCAAGTAATCGTCCAGGATTCCTCGACCAG

>VAR6
GACAGACTTGTCCAAAACGCCCACCACGAAGGTGCATAGTGAGAAGAGTAAGTCAAGA
GATAGACTTGTCCAAAAAGAAACGGAAGAGAAAGCTTGGGGAGACGCTCACGAAGGT
GCATAGTGAGAAGAGTAAGTCAAGAGATAGACTTGTTCAAAAAGAAACAGAAGAGAAT
GCTTGGGGTTACACTCACGAAGGTGCATAGTGAGAAGAGTAAGTCAAGAGACAGACTT
GTTCGAAAAGAAACAAAAGAGAATGCTTGGGGTTACACTCACGAAGGTGCATAGTGAG
AAGAGTAAGTCAAGAGACAGACTTGTTCGAAAAGAAACAAAAGAGAATGCTTGGGGA
GATAGAAGTGTGAGATAGTTCTCAAGCTAAGAAAGTTGTAAAAGCTAAGAACTAGCATC
AAATGATGGATGAAACACAAGGTAGTTGTTGAAAAGTCAAACACTTGGTGATATGAACA
CAAACGTTCAATATGACAAACCCATGCCAAGTAAAGAGAAAATGAAAACTGGTGATTGT
TGCGGAAATCGTCCAGGATTCCTCGACCAG
>VAR7
GACAGACTTGTCCAAAACGCCCACCACGAAGGTGCATAGTGAGAAGAGTAAGTCAAGA
GATAGACTTGTCCAAAAAGAAACGGAAGAGAAAGCTTGGGGAGACGCTCACGAAGGT
GCATAGTGAGAAGAGTAAGTCAAGAGATAGACTTGTAGAGAATGCTTGGGGTTACACTC
ACGAAGGTGCATAGTGAGAAGAGTAAGTCAAGAGACAGACTTGTTCAAAAAGAAACA
AAAGAGAATGCTTGGGGAGATAGAAGTGTGAGATAGTTCTCAAGCTAAGAAAGTTGTA
AAAGCTAAGAACTAGCAAGTAATCGTCCAGGATTCCTCGACCAG

(B) MUltiple Sequence Comparison by Log- Expectation (MUSCLE) alignment of *VAR6* and *VAR7* new variants compared to known *VAR1-5* described in Col-0

```
VAR1            GACAGACTTGTCCAAAACGCCCACCACGAAGGTGCATAGTGAGAAGAGTAAGTCAAGAGA
VAR2            GACAGACTTGTCCAAAACGCCCAC------------------------------------
VAR3            GACAGACTTGTCCAAAACGCCCACCACGAAGGTGCATAGTGAGAAGAGTAGGTCAAGAGA
VAR4            GACAGACTTGTCCAGAACGCCCACCACGAAGGTGCATAGTGAGAAGAGTAAGTCAAGAGA
VAR5            GACAGACTTGTCCAAAACGCCCAC------------------------------------
VAR6            GACAGACTTGTCCAAAACGCCCACCACGAAGGTGCATAGTGAGAAGAGTAAGTCAAGAGA
VAR7            GACAGACTTGTCCAAAACGCCCACCACGAAGGTGCATAGTGAGAAGAGTAAGTCAAGAGA
                ************** *********

VAR1            TAGACTTGTCCAAAAAGAAACGGAAGAGAAAGCGTGGGGAGACGCTCACGAAGGTGCATA
VAR2            ----------------------------------------------CGCGAAGGTGCATA
VAR3            TAGACTTGTCCAAAAAGAAACGGAAGAGAAAGCTTGGGGAGACGCTCACGAAGGTGCATA
VAR4            TAGACTTGTCCAAAAAGAAACGGAAGAGAAAGCTTGGGGAGACGCTCACGAAGGTGCATA
VAR5            ----------------------------------------------CGCGAAGGTGCATA
VAR6            TAGACTTGTCCAAAAAGAAACGGAAGAGAAAGCTTGGGGAGACGCTCACGAAGGTGCATA
VAR7            TAGACTTGTCCAAAAAGAAACGGAAGAGAAAGCTTGGGGAGACGCTCACGAAGGTGCATA
                                                              * ************

VAR1            GTGAGAAGAGTAAGTCAAGAGACAGACTTGTTCGAAAAGAAACAGA--------------
VAR2            GTGAGAAGAGTAAGTCAAGAGACAGACTTGTTCGAAAAGAAACAGA--------------
VAR3            GTGAGAAGAGTAGGTCAAGAGATAGACTTGTTCAAAAAGAAACAGA--------------
VAR4            GTGAGAAGAGTAAGTCAAGAGATAGACTTGTTCAAAAAGAAACAGAAGAGAATGCTTGGG
VAR5            GTGAGAAGAGTAAGTCAAGAGATAGACTTGTTCAAAAAGAAACAGA--------------
VAR6            GTGAGAAGAGTAAGTCAAGAGATAGACTTGTTCAAAAAGAAACAGAAGAGAATGCTTGGG
VAR7            GTGAGAAGAGTAAGTCAAGAGATAGACTTGT-----------------------------
                **  ***  **   ******** ********

VAR1            ------------------------------------------------------------
VAR2            ------------------------------------------------------------
VAR3            ------------------------------------------------------------
VAR4            GTTACACTCACGAAGGTGCATAGTGAGAAGAGTAAGTCAAGAGATAGACTTGTTCAAAAA
VAR5            ------------------------------------------------------------
VAR6            GTTACACTCACGAAGGTGCATAGTGAGAAGAGTAAGTCAAGAGACAGACTTGTTCGAAAA
VAR7            ------------------------------------------------------------
```

```
VAR1            --------AGAGAATGCTTGGGGTTACACTCACGAAGGTGCATAGTGAGAAGAGTAAGTC
VAR2            --------AGAGAATGCTTGGGGTTACACTCACGAAGGTGCATAGTGAGAAGAGTAAGTC
VAR3            --------AGAGAATGCTTGGGGTTACACTCACGAAGGTGCATAGTGAGAAGAGTAAGTC
VAR4            GAAACAAAAGAGAATGCTTGGGGTTACACTCACGAAGGTGCATAGTGAGAAGAGTAAGTC
VAR5            --------AGAGAATGCTTGGGGTTACACTCACGAAGGTGCATAGTGAGAAGAGTAAGTC
VAR6            GAAACAAAAGAGAATGCTTGGGGTTACACTCACGAAGGTGCATAGTGAGAAGAGTAAGTC
VAR7            --------AGAGAATGCTTGGGGTTACACTCACGAAGGTGCATAGTGAGAAGAGTAAGTC
                        ****************************************************

VAR1            AAGAGACAGACTTGTTCGAAAAGAAACAAAAGAGAATGCTTGGGGAGATAGAAGTGTGAG
VAR2            AAGAGACAGACTTGTTCGAAAAGAAACAAAAGAGAATGCTTGGGGAGATAGAAGTGTGAG
VAR3            AAGAGACAGACTTGTTCAAAAAGAAACGAAAGAGAATGCTTGGGGAGATAGAAGTGTGAG
VAR4            AAGAGACAGACTTGTTCAAAAAGAAACAAAAGAGAATGCTTGGGGAGATAGAAGTGTGAG
VAR5            AAGAGACAGACTTGTTCAAAAAGAAACAAAAGAGAATGCTTGGGGAGATAGAAGTGTGAG
VAR6            AAGAGACAGACTTGTTCGAAAAGAAACAAAAGAGAATGCTTGGGGAGATAGAAGTGTGAG
VAR7            AAGAGACAGACTTGTTCAAAAAGAAACAAAAGAGAATGCTTGGGGAGATAGAAGTGTGAG
                ***************** ********* ********************************

VAR1            ATAGTTCTCAAGCTAAGAAAGTTGTAAAAGCTAAGAACTAGCATCAAATGATGGATGAAA
VAR2            ATAGTTCTCAAGCTAAGAAAGTTGTAAAAGCTAAGAACTAGCATCAAATGATGGATGAAA
VAR3            ATAGTTCTCAAGCTAAGAAAGTTGTAAAAGCTAAGAACTAGCA-----------------
VAR4            ATAGTTCTCAAGCTAAGAAAGTTGTAAAAGCTAAGAACTAGCA-----------------
VAR5            ATAGTTCTCAAGCTAAGAAAGTTGTAAAAGCTAAGAACTAGCA-----------------
VAR6            ATAGTTCTCAAGCTAAGAAAGTTGTAAAAGCTAAGAACTAGCATCAAATGATGGATGAAA
VAR7            ATAGTTCTCAAGCTAAGAAAGTTGTAAAAGCTAAGAACTAGCA-----------------
                *******************************************

VAR1            CACAAGGTAGTTGTTGAAAAGTCAAACACTTGGTGATATGAACACAAACGTTCAATATGA
VAR2            CACAAGGTAGTTGTTGAGAAGTCAAACACTTGGTGATATGAACACAAACGTTCAATATGA
VAR3            ------------------------------------------------------------
VAR4            ------------------------------------------------------------
VAR5            ------------------------------------------------------------
VAR6            CACAAGGTAGTTGTTGAAAAGTCAAACACTTGGTGATATGAACACAAACGTTCAATATGA
VAR7            ------------------------------------------------------------


VAR1            CAAACCCATGCCAAGTAAAGAGAAAATGAAAACTGGTGATTGTTGCGGAAATCGTCCAGG
VAR2            CAAACCCATGCCAAGTAAAGAGAAAATGAAAACTGGTGATTGTTGCGGAAATCGTCCAGG
VAR3            ----------------------------------------------AGTAATCGTCCAGG
VAR4            ----------------------------------------------AGTAATCGTCCAGG
VAR5            ----------------------------------------------AGTAATCGTCCAGG
VAR6            CAAACCCATGCCAAGTAAAGAGAAAATGAAAACTGGTGATTGTTGCGGAAATCGTCCAGG
VAR7            ----------------------------------------------AGTAATCGTCCAGG
                                                               * ***********

VAR1            ATTCCTCGACCAG
VAR2            ATTCCTCGACCAG
VAR3            ATTCCTCCACCAG
VAR4            ATTCCTTCACCAG
VAR5            ATTCCTCGACCAG
VAR6            ATTCCTCGACCAG
VAR7            ATTCCTCGACCAG
                ******  *****
```
