# Supplementary material for: 45S rDNA Diversity In Natura as One Step towards Ribosomal Heterogeneity in Arabidopsis thaliana
Source: Plants (Basel). 2023 Jul 21;12(14):2722. doi: 10.3390/plants12142722 (PMC10386311; doi:10.3390/plants12142722)
Supplement: Supplementary file 1 [file plants-12-02722-s001.zip › Figure_S3.pdf]

Figure S3: 5'ETS variants identified in Brassicaceae Lineage I, Clade A, Camelineae and Brassicaceae Lineage II, Clade B.

(A) Fasta sequences of 5'ETS variants

<u>Brassicaceae Lineage I, Clade A, Camelineae</u>

AtVARA and AtVARB stands for the two variants known in *Arabidopsis thaliana.*

Ah1 and Ah2 are two independent cloned sequences for *Arabidopsis halleri*.

Al1-3 are two independent cloned sequences for *Arabidopsis lyrata.*

Ap1-4 are four independent cloned sequences for *Arabidopsis pedemontana.*

Ac1 is supported by two independent clones whereas Ac2 sequence was only cloned once for *Arabidopsis cebennensis.*

<u>Brassicaceae Lineage II, Clade B</u>

Bo was only cloned once and shared 100% sequence identity with the published sequence for *Brassica oleracea* [25].

(B) MUltiple Sequence Comparison by Log- Expectation (MUSCLE) alignments of 5'ETS variants grouping them in an alignment by length and whether they contain or not an insertion splitting away the conserved U3 snoRNP binding site A^{123}B from the primary processing site P. There is no insertion for *Brassica oleracea (*Bo*)* and *Arabidopsis cebennensis* (Ac2).

(A) Fasta sequences of 5'ETS variants

<u>Brassicaceae Lineage I, Clade A, Camelineae</u>

```
>At_VARA
CTTTTCCGGGCACTTTTCCGGCGATCGGTTTTGTTGCCTTTTTCCGAGTTTTCTCAGCAGTTCTCGGACAAAAACTGCTG
AATCGTCGAGGAGAATGGGCTTGCTTGCGTGGGCTGCCATTAGTTCTTCGAGGCGTTAGGGTGGCGGCGGTATAAAAGTG
TCGGAGTTTTTTCAGCAGTTCTCGGACAAAAATTGCTGAGTGGCCGAGAAGAATGGGCGTGTCATGCGTGGGCTGACATG
GATTCTTCGAGGCCTAGGGGTGGCGGTATATAACTTGTTCGCATGATATTACCGAGATGTCCCCACGGGCATCTTTTCAC
CTCGTCGCCGAAGAGAATGGGCGTGTCATGGCATGGGCTGACATGGATTCTCCTAGGCCGTTTGGGTGGCGGTATAGTCG
TCTTGCGCACGAAATACCGAGATGTCCCCATGGGCATCGATTCCACCCGCCTAGGTTGGATGGGCGTGCTTCGTCGGAAA
GCATGGATCCGCCTAGGCTGTCCCGAGTGTGAGCGAGGTGTGAGTGTCGCCCATGGGCATCGACACCTTGCGGCTAGGAA
CTGGAACGAGACGGGTGGCAAAGATTTCGAGTAGCACTTCATACTACCGTGGGTTTTTTAAACCTTCCGAGTTTTGTTGA
TGTTATTCCGAGAATTAGCAAACCGTAACGAAGATGTTCTTGGCAACCATCTTTTGATGGGAGTCCGGCTGTTCGATAGC
CGGCCAAGGGTGATGAACGAAATGTGAACCCTTGTCTCGCCTAGGTTGGATGGGCGTGCTTCGTTGGAAAGCATGGATCC
GCCTAGGCTGTCCCGAGTGTGAGCGAGGTGTGAGTGTCGCCCATGGGCATCGACACCTTGCGGCTAGGAACTGGAACGAG
ACGGGTAGCAAAGATTTCGAGTAGCACTTCATACTACCGTGGGTTTTTTAAACCTTCCTAGTTTTGTTGATGTTATTCCG
AGAATTAGCAAACCGTAACGAAGATGTTCTTGGCAACCATCTTTTGATGGGAGTCCGGCTGTTCGAAAGCCGGCCAAGGG
TGATGAACGAAATGTGAACCCTTGTCTCGCCTAGGTTGGATGGGCGTGCTTCGTTGGAAAGCATGGATCCGCCTAGGCTG
TCCCGAAGTATCTCGCGCTTGTACGGCTTTGGCTCGGATTCGTCCGTCTTCTTTCTTCTTAGCCGAGTACTTCGGTAGAT
TAGTTGGAACGATTGATGATTTTGAGTTAATTGAACGTTCGGCGTATGAGTGGTGATCGG
>At_VARB
CTTTTCCGGGCACTTTTCCGGCGATCGGTTTTGTTGCCTTTTTCCGAGTTTTCTCAGCAGTTCTCGGACAAAAACTGCTG
AATCGTCGAGGAGAATGGGCTTGCCTTGCGTGGGCTGCCATTAGTTCTTCGAGGCGTTAGGGTGGCGGCGGTATAAAAGT
GTCGGAGTTTTTTCAGCAGTTCTCGGACAAAAATTGCTGAGTGGCCGAGAAGAATGGGCGTGTCATGCGTGGGCTGACAT
GGATTCTTCGAGGCCTAGGGGTGGCGGTATATAACTTGTTCGCATGATATTACCGAGATGTCCCCACGGGCATCTTTTCA
CCTCGTCGCCGAAGAGAATGGGCGTGTCATGGCATGGGCTGACATGGATTCTCCTAGGCCGTTTGGGTGGCGGTATAGTC
GTCTTGCGCACGAAATACCGAGATGTCCCCATGGGCATCGATTCCACCCGCCTAGGTTGGATGGGCGTGCTTCGTTGGAA
AGCATGGATCCGCCTAGGCTGTCCCGAGTGTGAGCGAGGTGTGAGTGTCGCCCATGGGCATCGACACCTTGCGGCTAGGA
ACTGGAACGAGACGGGTRGCAAAGATTTCGAGTAGCACTTCATACTACCGTGGGTTTTTTAAACCTTCCCCTAGTTTTGT
TGATGTTATTCCGAGAATTAGCAAACCGTAACGAAGATGTTCTTGGCAACCATCTTTTGATGGGAGTCCGGCTGTTCGAA
AGCCGGCCAAGGGTGATGAACGAAATGTGAACCCTTGTCTCGCCTAGGTTGGATGGGCGTGCTTCGTTGGAAAGCATGGA
TCCGCCTAGGCTGACCCGAAGGTATCTCGCGCTTGTACGGCTTTGGCTCGGATTCGTCCGTCTTCTTTCTTCTTAGCCGA
GTACTTCGGTAGATTAGTTGGAACGATTGATGATTTTGAGTTAATTGAACGTTCGGCGTATGAGTGGTGATCGG
>Ah1
CTTTTCCGGGCACTTTTCCGGCGATCGGTTTTGTCGCAGTTTTCGGGCATTTCTCGGACAAAAATCGCTGAGTGGTCGCG
GAGAATGGGCGTGTCATGCGTGGGATGACATGGATTCTTCGAGGCCTTGGGGTGGCGGTATATAAGGAGATCGCACAATA
TACCGAGATGTCCCCACGGGCATCGTTTCACTCCGTCGCCGAAGAGAATGGGCGTGTCATGACATGGGCTGACATGGATT
CTCCAAGGCCGTGGGTGGCGGTACATTCGGAATGCACACGAAATACCGAGATGTCCCCATGGGCATCGATTCCACCCGCC
TAGGTTGGATGGGCGTGCTTCGTTGGAAAGCATGGATCCGCCTAGGCTGTCCCGGGGATGAGCAAGGTGTGAGTGTCGCC
```

CATGGGCATCGACACCTTGCGGCTTCGAACGGAAACGGAGGTAAGCACAGGATTGTACGCCGAGCGGGTGTCTTGGAGTC
ATCCAGTTTACCTTTTGCGGATGTTCATGGCAACCATCTTTTGATGGGAAGTGGATATCGGAAGTCAGTCTTTGACCGTG
AAGTGCCATGAAGTTCCCAGGCTTTGTGTATAACTTCCGGACTGCGGCTTCGATATTCGCAGCCTAGGTTGGATGGGCGT
GCGAAAGCATAGATCCGCCTAGGCTGTCCCGACGGTTTTTCGCGCTTGTGCGGCTTTGGCCTGGATTCGTCCGTCTTCTC
TCTTCTCAGCCGAATATTTCGGAGGAGTCGGGGAACGATTGATGTTTTTGGGTCAATTGAACGTTCGGCGTATGAGTGGT
GATCGG
>Ah2
CTTTTCCGGGCACTTTTCCGGCGATCGGTTTTGTCGCAGTTTTCGGGCATTTCTCGGACAAAAATCGCTGAGTGGTCGCG
GAGAATGGGCGTGTCATGCGTGGGATGACATGGATTCTTCGAGGCCTTGGGGTGGCGGTATATAAGGAGATCGCACAATA
TACCGAGATGTCCCCACGGGCATCGTTTCACTCCGTCGCCGAAGAGAATGGTCGTGTCATGACATGGGCTGACATGGATT
CTCCAAGGCCGTGGGTGGTGGTATATTCGGAATGCACACGAAATACCGAGATGTCCCCATGGGCATCGATTCCACCCGCC
TAGGTTGGATGGGCGTGCTTCGTTGGAAAGCATGGATCCGCCTAGGCTGTCCCGGGGATGAGCAAGGTGTGAGTGTCGCC
CATGGGCATCGACACCTTGCGGCTTCGAACGGAAACGGAGGTAAGCACAGGATTGTACGCCGAGCGGGTGTCTTGGAGTC
ATCCAGTTTACCTTTTGCGGATGTTCATGGCAACCATCTCTTGATGGGAAGTGGATATCGGAAGTCAGTCTTTGACCGTG
AAGTGCCATGAAGTTCCCAGGCTTTGTGTATAACTTCCGGACTGCGGCTTCGATATTCGCAGCCTAGGTTGGATGGGCGT
GCGAAAGCATAGATCCGCCTAGGCTGTCCCGACGGTTTTTCGCGCTTGTGCGGCTTTGGCCTGGATTCGTCCGTCTTCTC
TCTTCTCAGCCGAATATTTCGGAGGAGTCGGGGAACGATTGATGTTTTTGGGTCAATTGAACGTTCGGCGTATGAGTGGT
GATCGG
>Al1
CTTTTCCGGGCACTTTTCCGGCGATCGGTTTTGTTGCAGTTTTCGGAGTTTTTTCAGCAGTTCTCGGACAAAAATTGCTG
TGTGGTCGCGGAGAATGGGCGTGTCATGCGTGGGATGACATGGATTCTTCGAGGCCTTGGGGTGGCGGTATATTGGGAAA
GCGCATTGTATACCGAGATGTCCCCACGGGCATCGTTTCACCCCGTCGCCGAAGAGAATGGGCGTGTCATGACATGGGCT
GACATGGATTCTCCTAGGCCGTGGGTGGCGGTATATTCGGAATGCGCCCGAAATACCGAGATGTCCCCATGGGCATCGAT
TCCACCCGCCTAGGTTGGATGGGCGTGCTTCGTTGGAAAGCATGGATCCGCCTAGGCTGTCCCGAGTTGAGCAAGGTGTG
AGTGTCGCCCATGGGCATCGACACCTTGCGGCTTTGAACTGAAACGGAGGTACGCGCAGCTGTTACGTCGTGCAGGGTTT
CTCGGCGTCACTTCTAGCGGCTTTTGCGGATGTTCTTGGCAACCATCTTTTGATGGGGAAGTGGATATCGGCTGGTAGTG
ACACGAGGCTCCCGGTCTTGTGTGTATCCTTCGCACTGCGGCTCCGATATTCGCAGCCTAGGTTGGATGGGCGTGCGAAA
GCATGGATCTGCCTAGGCTGTCTCGACGGTTTTTCGCGCTTGTGCGGCTTTGGCTTGGATTCGTCCGTCTTCTCTCTTCT
CAGCCGAATATTTCGGAGGAGTCGGGGAACGATTGATGTTTTTGGGTCAATTGAACGTTCGGCGTATGAGTGGTGATCGG
>Al2
CTTTTCCGGGCACTTTTCCGGCGATCGGTTTTGTTGCAGTTTTTGAGTTTTTTCAGCAGTTCTCGGACAAAAATTGCTGT
GTGGTCGCGGAGAATGGGCGTGTCATGCGTGGGATGACATGGATTCTTCGAGGCCTTGGGGTGGCGGTATATTGGGAAAG
CGCATTGTATACCGAGATGTCCCCACGGGCATCGTTTCACCCCGTCGCCGAAGAGAATGGGCGTGTCATGACATGGGCTG
ACATGGATTCTCCTAGGCCGTGGGTGGCGGTATATTCGGAATGCGCCCGAAATACCGAGATGTCCCCATGGGCATCGATT
CCACCCGCCTAGGTTGGATGGGCGTGCTTCGTTGGAAAGCATGGATCCGCCTAGGCTGTCCCGAGTTGAGCAAGGTGTGA
GTGTCGCCCATGGGCATCGACACCTTGCGGCTTTGAACTGAAACGGAGGTACGCGCAGCTGTTACGTCGTGCAGGGTTTC
TCGGCGTCACTTCTAGCGGCTTTTGCGGATGTTCTTGGCAACCATCTTTTGATGGGGAAGTGGATATCGGCTGGTAGTGA
CACGAGGCTCCCGGTCTTGTGTGTATCCTTCGCACTGCGGCTCCGATATTCGCAGCCTAGGTTGGATGGGCGTGCGAAAG
CATGGATCTGCCTAGGCTGTCTCGACGGTTTTTCGCGCTTGTGCGGCTTTGGCTTGGATTCGTCCGTCTTCTCTCTTCTC
AGCCGAATATTTCGGAGGAGTCGGGGAACGATTGATGTTTTTGGGTCAATTGAACGTTCGGCGTATGAGTGGTGATCGG
>Al3
CTTTTCCGGGCACTTTTCCGGCGATCGGTTTTGTTGCAGTTTTCGGAGTTTTTTCAGCAGTTCTCGGACAAAAATTGCTG
AGTGGTCGCGGAGAATGGGCGTGTCAAGCGTGGGATGACATGGATTCTTCGAGACCTTGGGGTGGCGGTATATAACTCTC
CACGCATGGTATACCGAGATGTCCCCACGGGCATCGTTTCACCCCGTCGCCGAAGAGAATGGGCGTGTCATGACATGGGC
TGACATGGATTCTCCTAGGCCGTGGGTGGCGGTATATTCGGAATGCGCCCGAAATACCGAGATGTCCCCATGGGCATCGA
TTCCACCCGCCTAGGTTGGATGGGCGTGCTTCGTTGGAAAGCATGGATCCGCCTAGGCTGTCCCGAGTTGAGCAAGGTGT
GAGTGTCGCCCATGGGCATCGACACCTTGCGGCTTTGAACTGAAACGGAGGTACGCGCAGCTGTTACGTCGTGCAGGGTT
TCTCGGCGTCACTTCTAGCGGCTTTTGCGGATGTTCTTGGCAACCATCTTTTGATGGGGAAGTGGATATCGGCTGGTAGT
GACACGAGGCTCCCGGTCTTGTGTGTATCCTTCGCACTGCGGCTCCGATATTCGCAGCCTAGGTTGGATGGGCGTGCGAA
AGCATGGATCTGCCTAGGCTGTCTCGACGGTTTTTCGCGCTTGTGCGGCTTTGGCTTGGATTCGTCCGTCTTCTCTCTTC
TCAGCCGAATATTTCGGAGGAGTCGGGGAACGATTGATGTTTTTGGGTCAATTGAACGTTCGGCGTATGAGTGGTGATCG
G
>Ap1
CTTTTCCGGGCACTTTTCCGGCGATCGGTTTTGTCGCAGTTTTCCGAGTTTTTTCAGCAGTTCTCGGACAAAAATTGCTG
TGTGGTCGCGGAGAATGGGCGTGTCATGCGTGGGATGACATGGATTCTTCGAGGCCTTGGGGTGGCGGTATATAAGGAGA
GCGCATGGTATACCGAGATGTCTCCACGGGCATCGTTTCACTCCGTTGCCGAAGAGAATGGGCGTGTCATGACATGGGCT
GACATGGATTCTCCTAGGCCGTGGGTGGCGGTATATTCGGAATGCGCACGATATACCGAGATGTCCCCATGGGCATCGAT
TCCACCCGCCTAGGTTGGATGGGCGTGCTTCGTTGGAAAGCATGGATCTGCCTAGGCTGTCCCGGGGTGAGCAAGGTTTG
AGTGTCGCTCATGGGCATCGACACCTTGCGTCTTTGAACGGAAGCGAAGGTAAGCGGATGATTGACGACGAGCGGGTCTC
TCGGAGTTGTCTAGTTTACCTTTTGCGGATGTTTTTGGCAACCATCTTTCGATGGGAAGTGGATATCGGAAGTCAGTCTT
TGGACCGAGAAGTTACACGAAGTACCCAGGCTTTGTGCGTAACTTCCGCACTGCGGCTACGATATTCGCAGCCTAGGTTG
GATGGGCGTGCTAAAGCATGGATCCGCCTAGGCTGTCTTGACGGTTTTTCGCGCTTGTGCGGCTTTGGCTTGGATTCGTC

CGTCTTCTCTCTTCTCAGCCGAATATTTCGGAGGAGTCGGGGAACGATTGATGTTTTTGGGTCAATTGAACGTTCGGCGT
ATGAGTGGTGATCGG
>Ap2
CTTTTCCGGGCACTTTTCCGGCGATCGGTTTTGTGTCAGTTTTCCGAGTTTTTTCAGCAGTTCTCGGACAAAAATTGCTG
TGTGGTCGCGGAGAATGGGCGTGTCATGCGTGGGATGACATGGATTCTTCGAGGCCTTGGGGTGGCGGTATATAAGGAGA
GCGCATGGTATACCGAGATGTCTCCACGGGCATCGTTTCACTCCGTTGCCGAAGAGAATGGGCGTGTCATGACATGGGCT
GACATGGATTCTCCTAGGCCGTGGGTGGCGGTATATTCGGAATGCGCACGATATACCGAGATGTCCCCATGGGCATCGAT
TCCACCCGCCTAGGTTGGATGGGCGTGCTTCGTTGGAAAGCATGGATCTGCCTAGGCTGTCCCGGGGTGAGCAAGGTTTG
AGTATCGCTCATGGGCATCGACACCTTGCGTCTTTGAACGGAAGCGAAGGTAAGCGGATGATTGACGACGAGCGGGTCTC
TCGGAGTTGTCTAGTTTACCTTTTGCGGATGTTTTTGGGAACCATCTTTCGATGGGAAGTGGATATCGGAAGTCAGTCTT
TGGACCGAGAAGTTACACGAAGTACCCAGGCTTTGTGCGTAACTTCCGCACTGCGGCTACGATATTCGCAGCCTAGGTTG
GATGGGCGTGCTAAAGCATGGATCCGCCTAGGCTGTCTCGACGGTTTTTCGCGCTTGTGCGGCTTTGGCTTGGATTCGTC
CGTCTTCTCTCTTCTCAGCCGAATATTTCGGAGGAGTCGGGGAACGATTGATGTTTTTGGGTCAATTGAACGTTCGGCGT
ATGAGTGGTGATCGG
>Ap3
CTTTTCCGGGCACTTTTCCGGCGATCGGTTTTGTCGCAGTTTTCCGAGTTTTTTCAGCAGTTCTCGGACAAAAATTGCTG
TGTGGTCGCGGAGAATGGGCGTGTCATGCGTGGGATGACATGGATTCTTCGAGGCCTTGGGGTGGCGGTATATAAGGAGA
GCGCATGGTATACCGAGATGTCTCCACGGGCATCGTTTCACTCCGTTGCCGAAGAGAATGGGCGTGTCATGACATGGGCT
GACATGGATTCTCCTAGGCCGTGGGTGGCGGTATATTCGGAATGCGCACGATATACCGAGTTGTCCCCATGGGCATCGAT
TCCACCCGCCTAGGTTGGATGGGCGTGCTTCGTTGGAAAGCATGGATCTGCCTAGGCTGTCCCGGGGTGAGCAAGGTGTG
AGTGTCGCTCATGGGCATCGACACCTTGCGTCTTTGAACGGAAGCGAAGGTAAGCGGATGATTGACGACGAGCGGGTCTC
TCGGAGTTGTCTAGTTTACCTTTTGCGGATGTTTTTGGCAACCATCTTTCGATGGGAAGTGGATATCGGAAGTCAGTCTT
TGGACCGAGAAGTTACACGAAGTACCCAGGCTTTGTGCGTAACTTCCGCACTGCGGCTACGATATTCGCAGCCTAGGTTG
GATGGGCGTGCTAAAGCATGGATCCGCCTTGGCTGTCTTGACGGTTTTTCGCGCTTGTGCGGCTTTGGCTTGGATTCGTC
CGTCTTCTCTCTTCTCAGCCGAATATTTCGGAGGAGTCGGGGAACGATTGATGTTTTTGGGTCAATTGAACGTTCGGCGT
ATGAGTGGTGATCGG
>Ap4
CTTTTCCGGGCACTTTTCCGGCGATCGGTTTTGTGTCAGTTTTCCGAGTTTTTTCAGCAGTTCTCGGACAAAAATTGCTG
TGTGGTCGCGGAGAATGGGCGTGTCATGCGTGGGATGACATGGATTCTTCGAGGCCTTGGGGTGGCGGTATATAAGGAGA
GCGCATGGTATACCGAGATGTCTCCACGGGCATCGTTTCACTCCGTTGCCGAAGATAATGGGCGTGTCATGACATGGGCT
GACATGGATTCTCCTAGGCCGTGGGTGGCGGTATATTCGGAATGCGCACGATATACCGAGATGTCCCCATGGGCATCGAT
TCCACCCGCCTAGGTTGGATGGGCGTGCTTCGTTGGAAAGCATGGATCTGCCTAGGCTGTCCCGGGGTGAGCAAGGTTTG
AGTGTCGCTCATGGGCATCGACACCTTGCGTCTTTGAACGGAAGCGAAGGTAAGCGGATGATTGACGACGAGCGGGTCTC
TCGGAGTTGTCTAGTTTACCTTTTGCGGATGTTTTTGGCAACCATCTTTCGATGGGAAGTGGATATCGGAAGTCAGTCTT
TGGACCGAGAAGTTACACGAAGTACCCAGGCTTTGTGCGTAACTTCCGCACTGCGGCTACGATATTCGCAGCCTAGGTTG
GATGGGCGTGCTAAAGCATGGATCCGCCTAGGCTGTCTTGACGGTTTTTCGCGCTTGTGCGGCTTTGGCTTGGATTCGTC
CGTCTTCTCTCTTCTCAGCCGAATATTTCGGAGGAGTCGGGGAACGATTGATGTTTTTGGGTCAATTGAACGTTCGGCGT
ATGAGTGGTGATCGG
>Ac1
CTTTTCCGGGCACTTTTCCGGCGATCGGTTTGGTCGCAGTTTTCCGAGTTTTTTCAGCAGTTCTCGGACAAAAATTGCTG
TGTGGTCGCGGAGAATGGGCGTGTCATGCGTGGGATGACATGGATTCTTCGAGGCCTTGGGGTGGCGGTATATAAGGAGA
GCGCATGGTATACCGAGATGTCCCCACGGGCATCGTTTCACTCCCTTGCCGAAGAGAATGGGCGTGTCATGACATGGGCT
GACATGGATTCTCCTAGGCCGTGGGTGGCTGTATATTCGGGGAATGCGCACGAAATACCGAGATGTCCCCATGGGCATCG
ATTCCACCCGCCTAGGTTGGATGGGCGTGCTTCGTTGGAAAGCATGGATCTGCCTAGGCTGTCCCGGGGTGAGCAAGGTG
TGAGTGTCGCCCATGGGCATCGACACCTTGCGTCTTTGAACGGAAACGAAGGTAAGCAGAGGATTGACGACGAGCGGGTC
TCTCGGTGTTTTCTAGTTTACCTTTAGCGGATGTTCTTTGGCAACCATCTTTCGATGGGAAGTGGATATCGGAAGTCAGT
CTTTGGACCGAGAAGTGACACGAAGTACCCAGGCTTTGTGTGTAACTTCCGCACTGCGGCTACGATATTCGCAGCCTAGG
TTGGATGGGCGTGCGAAAGCATGGATCCGCCTAGGCTGTCTTGACGGTTTTTCGCGCTTGTGCGGCTTTGGCTTGGATTC
GTCCGTCTTCTCTCTTCTCAGCCGAATATTTCGGAGGAGTCGGGGAACGATTGATGTTTTTGGGTCAATTGAACGTTCGG
CGTATGAGTGGTGATCGG
>Ac2
CTTTTCCGGGCACTTTTCCGGTGGACCATTTTGCCCCTAAAATCAAATTTTCGCGCTTGTGTGGTCTTGGCCTGGTTTCA
TCCGTCTTCCAGCTGCTCTTTTGATTACATCTCGAGAGTGGTTGGAAAGATTGATGTTAGCGGGGCAATGAACGTTCGGC
GTATGAGTGGTGATCGA

Brassicaceae Lineage II, Clade B

>Bo
CTTTTCCGGGCACTTTTCCGGCGACTTTTCCGGTGGACCATTTTGCCCCTAAAATCAAATTTTCGCGCTTGTGTGGTCTT
GCCCTGGTTTCATCCGTCTTCCAGCTGCTCTTTTGATTACATCTCGAGAGTGGTTGGAAAGATTGATGTTAGCGGGGCAA
TTGAACGTGCGGCGTATGAGTGGTGATCGG

(B) MUSCLE alignment of 5'ETS variants

Arabidopsis genus variants with the exception of the *A. cebennensis* insertion free Ac2 variant

```
                        A2          A3              B
At_VARA         CTTTTCCGGGCACTTTTCCGGCGATCGGTTTTGTTGCCTTTTTCCGAGTTTTCTCAGCAG
At_VARB         CTTTTCCGGGCACTTTTCCGGCGATCGGTTTTGTTGCCTTTTTCCGAGTTTTCTCAGCAG
Ac1             CTTTTCCGGGCACTTTTCCGGCGATCGGTTTGGTCGC---------AGTTT---------
Ah1             CTTTTCCGGGCACTTTTCCGGCGATCGGTTTTGTCGC---------AGTTT---------
Ah2             CTTTTCCGGGCACTTTTCCGGCGATCGGTTTTGTCGC---------AGTTT---------
Al1             CTTTTCCGGGCACTTTTCCGGCGATCGGTTTTGTTGC---------AGTTT---------
Al2             CTTTTCCGGGCACTTTTCCGGCGATCGGTTTTGTTGC---------AGTTT---------
Al3             CTTTTCCGGGCACTTTTCCGGCGATCGGTTTTGTTGC---------AGTTT---------
Ap1             CTTTTCCGGGCACTTTTCCGGCGATCGGTTTTGTCGC---------AGTTT---------
Ap2             CTTTTCCGGGCACTTTTCCGGCGATCGGTTTTGTGTC---------AGTTT---------
Ap3             CTTTTCCGGGCACTTTTCCGGCGATCGGTTTTGTCGC---------AGTTT---------
Ap4             CTTTTCCGGGCACTTTTCCGGCGATCGGTTTTGTGTC---------AGTTT---------
                ******************************* **  *         *****

                                       D1a Gruendler
At_VARA         TTCTCGGACAAAAACTGCTGAATCGTCGAGGAGAATGGGCTTG-CTTGCGTGGGCTGCCA
At_VARB         TTCTCGGACAAAAACTGCTGAATCGTCGAGGAGAATGGGCTTGCCTTGCGTGGGCTGCCA
Ac1             ------------------------------------------------------------
Ah1             ------------------------------------------------------------
Ah2             ------------------------------------------------------------
Al1             ------------------------------------------------------------
Al2             ------------------------------------------------------------
Al3             ------------------------------------------------------------
Ap1             ------------------------------------------------------------
Ap2             ------------------------------------------------------------
Ap3             ------------------------------------------------------------
Ap4             ------------------------------------------------------------

At_VARA         TTAGTTCTTCGAGGCGTTAGGGTGGCGGCGGTATAAAAGTGTCGGAGTTTTTTCAGCAGT
At_VARB         TTAGTTCTTCGAGGCGTTAGGGTGGCGGCGGTATAAAAGTGTCGGAGTTTTTTCAGCAGT
Ac1             -----------------------------------------TCCGAGTTTTTTCAGCAGT
Ah1             -----------------------------------------TCGG----------GCATT
Ah2             -----------------------------------------TCGG----------GCATT
Al1             -----------------------------------------TCGGAGTTTTTTCAGCAGT
Al2             -----------------------------------------TT-GAGTTTTTTCAGCAGT
Al3             -----------------------------------------TCGGAGTTTTTTCAGCAGT
Ap1             -----------------------------------------TCCGAGTTTTTTCAGCAGT
Ap2             -----------------------------------------TCCGAGTTTTTTCAGCAGT
Ap3             -----------------------------------------TCCGAGTTTTTTCAGCAGT
Ap4             -----------------------------------------TCCGAGTTTTTTCAGCAGT
                                                         *  *          *** *

                                        D1b Gruendler
At_VARA         TCTCGGACAAAAATTGCTGAGTGGCCGAGAAGAATGGGCGTGTCATGCGTGGGCTGACAT
At_VARB         TCTCGGACAAAAATTGCTGAGTGGCCGAGAAGAATGGGCGTGTCATGCGTGGGCTGACAT
Ac1             TCTCGGACAAAAATTGCTGTGTGGTCGCGGAGAATGGGCGTGTCATGCGTGGGATGACAT
Ah1             TCTCGGACAAAAATCGCTGAGTGGTCGCGGAGAATGGGCGTGTCATGCGTGGGATGACAT
Ah2             TCTCGGACAAAAATCGCTGAGTGGTCGCGGAGAATGGGCGTGTCATGCGTGGGATGACAT
Al1             TCTCGGACAAAAATTGCTGTGTGGTCGCGGAGAATGGGCGTGTCATGCGTGGGATGACAT
Al2             TCTCGGACAAAAATTGCTGTGTGGTCGCGGAGAATGGGCGTGTCATGCGTGGGATGACAT
Al3             TCTCGGACAAAAATTGCTGAGTGGTCGCGGAGAATGGGCGTGTCAAGCGTGGGATGACAT
Ap1             TCTCGGACAAAAATTGCTGTGTGGTCGCGGAGAATGGGCGTGTCATGCGTGGGATGACAT
Ap2             TCTCGGACAAAAATTGCTGTGTGGTCGCGGAGAATGGGCGTGTCATGCGTGGGATGACAT
Ap3             TCTCGGACAAAAATTGCTGTGTGGTCGCGGAGAATGGGCGTGTCATGCGTGGGATGACAT
Ap4             TCTCGGACAAAAATTGCTGTGTGGTCGCGGAGAATGGGCGTGTCATGCGTGGGATGACAT
                ************** **** **** ** * *************** ******* ******
```

```
At_VARA     GGATTCTTCGAGGCCTAGGGGTGGCGGTATATAACT-TGTTCGCATGATATTACCGAGAT
At_VARB     GGATTCTTCGAGGCCTAGGGGTGGCGGTATATAACT-TGTTCGCATGATATTACCGAGAT
Ac1         GGATTCTTCGAGGCCTTGGGGTGGCGGTATATAAGG-AGAGCGCATGGTA-TACCGAGAT
Ah1         GGATTCTTCGAGGCCTTGGGGTGGCGGTATATAAGG-AGATCGCACAATA-TACCGAGAT
Ah2         GGATTCTTCGAGGCCTTGGGGTGGCGGTATATAAGG-AGATCGCACAATA-TACCGAGAT
Al1         GGATTCTTCGAGGCCTTGGGGTGGCGGTATATTGGG-AAAGCGCATTGTA-TACCGAGAT
Al2         GGATTCTTCGAGGCCTTGGGGTGGCGGTATATTGGG-AAAGCGCATTGTA-TACCGAGAT
Al3         GGATTCTTCGAGACCTTGGGGTGGCGGTATATAACTCTCCACGCATGGTA-TACCGAGAT
Ap1         GGATTCTTCGAGGCCTTGGGGTGGCGGTATATAAGG-AGAGCGCATGGTA-TACCGAGAT
Ap2         GGATTCTTCGAGGCCTTGGGGTGGCGGTATATAAGG-AGAGCGCATGGTA-TACCGAGAT
Ap3         GGATTCTTCGAGGCCTTGGGGTGGCGGTATATAAGG-AGAGCGCATGGTA-TACCGAGAT
Ap4         GGATTCTTCGAGGCCTTGGGGTGGCGGTATATAAGG-AGAGCGCATGGTA-TACCGAGAT
            ************ *** ***************         ****   ** *********

                                       D2a Gruendler
At_VARA     GTCCCCACGGGCATCTTTTCACCTCGTCGCCGAAGAGAATGGGCGTGTCATGGCATGGGC
At_VARB     GTCCCCACGGGCATCTTTTCACCTCGTCGCCGAAGAGAATGGGCGTGTCATGGCATGGGC
Ac1         GTCCCCACGGGCATCGTTTCACTCCCTTGCCGAAGAGAATGGGCGTGTCATGACATGGGC
Ah1         GTCCCCACGGGCATCGTTTCACTCCGTCGCCGAAGAGAATGGGCGTGTCATGACATGGGC
Ah2         GTCCCCACGGGCATCGTTTCACTCCGTCGCCGAAGAGAATGGTCGTGTCATGACATGGGC
Al1         GTCCCCACGGGCATCGTTTCACCCCGTCGCCGAAGAGAATGGGCGTGTCATGACATGGGC
Al2         GTCCCCACGGGCATCGTTTCACCCCGTCGCCGAAGAGAATGGGCGTGTCATGACATGGGC
Al3         GTCCCCACGGGCATCGTTTCACCCCGTCGCCGAAGAGAATGGGCGTGTCATGACATGGGC
Ap1         GTCTCCACGGGCATCGTTTCACTCCGTTGCCGAAGAGAATGGGCGTGTCATGACATGGGC
Ap2         GTCTCCACGGGCATCGTTTCACTCCGTTGCCGAAGAGAATGGGCGTGTCATGACATGGGC
Ap3         GTCTCCACGGGCATCGTTTCACTCCGTTGCCGAAGAGAATGGGCGTGTCATGACATGGGC
Ap4         GTCTCCACGGGCATCGTTTCACTCCGTTGCCGAAGATAATGGGCGTGTCATGACATGGGC
            *** *********** ******  * * ******** ***** ********* *******

                                                  D12 Gruendler
At_VARA     TGACATGGATTCTCCTAGGCCGTTTGGGTGGCGGTATAGTC--GTCTTGCGCACGAAATA
At_VARB     TGACATGGATTCTCCTAGGCCGTTTGGGTGGCGGTATAGTC--GTCTTGCGCACGAAATA
Ac1         TGACATGGATTCTCCTAGGCCG--TGGGTGGCTGTATATTCGGGGAATGCGCACGAAATA
Ah1         TGACATGGATTCTCCAAGGCCG--TGGGTGGCGGTACATTC--GGAATGCACACGAAATA
Ah2         TGACATGGATTCTCCAAGGCCG--TGGGTGGTGGTATATTC--GGAATGCACACGAAATA
Al1         TGACATGGATTCTCCTAGGCCG--TGGGTGGCGGTATATTC--GGAATGCGCCCGAAATA
Al2         TGACATGGATTCTCCTAGGCCG--TGGGTGGCGGTATATTC--GGAATGCGCCCGAAATA
Al3         TGACATGGATTCTCCTAGGCCG--TGGGTGGCGGTATATTC--GGAATGCGCCCGAAATA
Ap1         TGACATGGATTCTCCTAGGCCG--TGGGTGGCGGTATATTC--GGAATGCGCACGATATA
Ap2         TGACATGGATTCTCCTAGGCCG--TGGGTGGCGGTATATTC--GGAATGCGCACGATATA
Ap3         TGACATGGATTCTCCTAGGCCG--TGGGTGGCGGTATATTC--GGAATGCGCACGATATA
Ap4         TGACATGGATTCTCCTAGGCCG--TGGGTGGCGGTATATTC--GGAATGCGCACGATATA
            *************** ******  *******  *** * **  *   *** * *** ***

                   D2b Gruendler                        C3 repeat     28
At_VARA     CCGAGATGTCCCCATGGGCATCGATTCCACCCGCCTAGGTTGGATGGGCGTGCTTCGTCG
At_VARB     CCGAGATGTCCCCATGGGCATCGATTCCACCCGCCTAGGTTGGATGGGCGTGCTTCGTTG
Ac1         CCGAGATGTCCCCATGGGCATCGATTCCACCCGCCTAGGTTGGATGGGCGTGCTTCGTTG
Ah1         CCGAGATGTCCCCATGGGCATCGATTCCACCCGCCTAGGTTGGATGGGCGTGCTTCGTTG
Ah2         CCGAGATGTCCCCATGGGCATCGATTCCACCCGCCTAGGTTGGATGGGCGTGCTTCGTTG
Al1         CCGAGATGTCCCCATGGGCATCGATTCCACCCGCCTAGGTTGGATGGGCGTGCTTCGTTG
Al2         CCGAGATGTCCCCATGGGCATCGATTCCACCCGCCTAGGTTGGATGGGCGTGCTTCGTTG
Al3         CCGAGATGTCCCCATGGGCATCGATTCCACCCGCCTAGGTTGGATGGGCGTGCTTCGTTG
Ap1         CCGAGATGTCCCCATGGGCATCGATTCCACCCGCCTAGGTTGGATGGGCGTGCTTCGTTG
Ap2         CCGAGATGTCCCCATGGGCATCGATTCCACCCGCCTAGGTTGGATGGGCGTGCTTCGTTG
Ap3         CCGAGTTGTCCCCATGGGCATCGATTCCACCCGCCTAGGTTGGATGGGCGTGCTTCGTTG
Ap4         CCGAGATGTCCCCATGGGCATCGATTCCACCCGCCTAGGTTGGATGGGCGTGCTTCGTTG
            ***** **************************************************** *
```

```
                         C3 repeat                       C1 repeat
At_VARA         GAAAGCATGGATCCGCCTAGGCTGTCCCGAGTGTGAGCGAGGTGTGAGTGTCGCCCATGG
At_VARB         GAAAGCATGGATCCGCCTAGGCTGTCCCGAGTGTGAGCGAGGTGTGAGTGTCGCCCATGG
Ac1             GAAAGCATGGATCTGCCTAGGCTGTCCCGGGG-TGAGCAAGGTGTGAGTGTCGCCCATGG
Ah1             GAAAGCATGGATCCGCCTAGGCTGTCCCGGGGATGAGCAAGGTGTGAGTGTCGCCCATGG
Ah2             GAAAGCATGGATCCGCCTAGGCTGTCCCGGGGATGAGCAAGGTGTGAGTGTCGCCCATGG
Al1             GAAAGCATGGATCCGCCTAGGCTGTCCCGAGT-TGAGCAAGGTGTGAGTGTCGCCCATGG
Al2             GAAAGCATGGATCCGCCTAGGCTGTCCCGAGT-TGAGCAAGGTGTGAGTGTCGCCCATGG
Al3             GAAAGCATGGATCCGCCTAGGCTGTCCCGAGT-TGAGCAAGGTGTGAGTGTCGCCCATGG
Ap1             GAAAGCATGGATCTGCCTAGGCTGTCCCGGGG-TGAGCAAGGTTTGAGTGTCGCTCATGG
Ap2             GAAAGCATGGATCTGCCTAGGCTGTCCCGGGG-TGAGCAAGGTTTGAGTATCGCTCATGG
Ap3             GAAAGCATGGATCTGCCTAGGCTGTCCCGGGG-TGAGCAAGGTGTGAGTGTCGCTCATGG
Ap4             GAAAGCATGGATCTGCCTAGGCTGTCCCGGGG-TGAGCAAGGTTTGAGTGTCGCTCATGG
                ************* **************  *  ***** **** ***** **** *****

                                                            130
At_VARA         GCATCGACACCTTGCGGCTAGGAACTGGAACGAGACGGGTGGCAAAGATTTCGAGTAGCA
At_VARB         GCATCGACACCTTGCGGCTAGGAACTGGAACGAGACGGGTAGCAAAGATTTCGAGTAGCA
Ac1             GCATCGACACCTTGCGTCTTTGAACGGAAACGAA--GGTAAGCAGAGGATT------G-A
Ah1             GCATCGACACCTTGCGGCTTCGAACGGAAACGGA--GGTAAGCACAGGATT------GTA
Ah2             GCATCGACACCTTGCGGCTTCGAACGGAAACGGA--GGTAAGCACAGGATT------GTA
Al1             GCATCGACACCTTGCGGCTTTGAACTGAAACGGA--GGTACGCGCAGCTGT-------TA
Al2             GCATCGACACCTTGCGGCTTTGAACTGAAACGGA--GGTACGCGCAGCTGT-------TA
Al3             GCATCGACACCTTGCGGCTTTGAACTGAAACGGA--GGTACGCGCAGCTGT-------TA
Ap1             GCATCGACACCTTGCGTCTTTGAACGGAAGCGAA--GGTAAGCGGATGATT------G-A
Ap2             GCATCGACACCTTGCGTCTTTGAACGGAAGCGAA--GGTAAGCGGATGATT------G-A
Ap3             GCATCGACACCTTGCGTCTTTGAACGGAAGCGAA--GGTAAGCGGATGATT------G-A
Ap4             GCATCGACACCTTGCGTCTTTGAACGGAAGCGAA--GGTAAGCGGATGATT------G-A
                **************** **  **** * * **    **   **  *    *        *

At_VARA         CTTCATACTACCGTGGGTTTTTTAAACCTTCC--GAGTTTTGTTGATGTTATTCCGAGAA
At_VARB         CTTCATACTACCGTGGGTTTTTTAAACCTTCCCCTAGTTTTGTTGATGTTATTCCGAGAA
Ac1             CGACGAGC------GGGTCTCTCGGTGTTT--TCTAGTTTACCTTTAGC-----------
Ah1             CGCCGAGC------GGGTGTCTTGGAGTCA--TCCAGTTTACCTTTTGC-----------
Ah2             CGCCGAGC------GGGTGTCTTGGAGTCA--TCCAGTTTACCTTTTGC-----------
Al1             CGTCGTGCA-----GGGTTTCTCGGCGTCACTTCTAG--CGGCTTTTGC-----------
Al2             CGTCGTGCA-----GGGTTTCTCGGCGTCACTTCTAG--CGGCTTTTGC-----------
Al3             CGTCGTGCA-----GGGTTTCTCGGCGTCACTTCTAG--CGGCTTTTGC-----------
Ap1             CGACGAGC------GGGTCTCTCGGAGTTG--TCTAGTTTACCTTTTGC-----------
Ap2             CGACGAGC------GGGTCTCTCGGAGTTG--TCTAGTTTACCTTTTGC-----------
Ap3             CGACGAGC------GGGTCTCTCGGAGTTG--TCTAGTTTACCTTTTGC-----------
Ap4             CGACGAGC------GGGTCTCTCGGAGTTG--TCTAGTTTACCTTTTGC-----------
                *  *   *      **** * *           * **      *   *

At_VARA         TTAGCAAACCGTAACGAAGATGTTC-TTGGCAACCATCTTTTGATGGGAGTCCGGCTGTT
At_VARB         TTAGCAAACCGTAACGAAGATGTTC-TTGGCAACCATCTTTTGATGGGAGTCCGGCTGTT
Ac1             -----------------GGATGTTCTTTGGCAACCATCTTTCGATGGGAA-GTGGATA-T
Ah1             -----------------GGATGTTC-ATGGCAACCATCTTTTGATGGGAA-GTGGATA-T
Ah2             -----------------GGATGTTC-ATGGCAACCATCTCTTGATGGGAA-GTGGATA-T
Al1             -----------------GGATGTTC-TTGGCAACCATCTTTTGATGGGGAAGTGGATA-T
Al2             -----------------GGATGTTC-TTGGCAACCATCTTTTGATGGGGAAGTGGATA-T
Al3             -----------------GGATGTTC-TTGGCAACCATCTTTTGATGGGGAAGTGGATA-T
Ap1             -----------------GGATGTTT-TTGGCAACCATCTTTCGATGGGAA-GTGGATA-T
Ap2             -----------------GGATGTTT-TTGGGAACCATCTTTCGATGGGAA-GTGGATA-T
Ap3             -----------------GGATGTTT-TTGGCAACCATCTTTCGATGGGAA-GTGGATA-T
Ap4             -----------------GGATGTTT-TTGGCAACCATCTTTCGATGGGAA-GTGGATA-T
                                  ******   *** ******** * ******     ** *
```

```
At_VARA         CGATAGCCGGCC---------AAGGGTGATGAACGAAATGTGAACCCTTGTCTCGCCTAG
At_VARB         CGAAAGCCGGCC---------AAGGGTGATGAACGAAATGTGAACCCTTGTCTC------
Ac1             CGGAAGTCAGTCTTTGGACCGAGAAGTGAC--ACGAAGT---------------ACCCAG
Ah1             CGGAAGTCAGTCTTT-GACCGTGAAGTGCC--ATGAAGT---------------TCCCAG
Ah2             CGGAAGTCAGTCTTT-GACCGTGAAGTGCC--ATGAAGT---------------TCCCAG
Al1             CGG--------CT-GGT-------AGTGAC--ACGAGGC---------------TCCC-G
Al2             CGG--------CT-GGT-------AGTGAC--ACGAGGC---------------TCCC-G
Al3             CGG--------CT-GGT-------AGTGAC--ACGAGGC---------------TCCC-G
Ap1             CGGAAGTCAGTCTTTGGACCGAGAAGTTAC--ACGAAGT---------------ACCCAG
Ap2             CGGAAGTCAGTCTTTGGACCGAGAAGTTAC--ACGAAGT---------------ACCCAG
Ap3             CGGAAGTCAGTCTTTGGACCGAGAAGTTAC--ACGAAGT---------------ACCCAG
Ap4             CGGAAGTCAGTCTTTGGACCGAGAAGTTAC--ACGAAGT---------------ACCCAG
                *       * *               **     * **

                                      C3 repeat
At_VARA         GTTGGATGGGCGTGCTTCGTTGGAAAGCATGGATCCGCCTAGGCTGTCCCGAGTGTGAGC
At_VARB         ------------------------------------------------------------
Ac1             GCTTTGTGTGTAACTTCCG-----------------------------------------
Ah1             GCTTTGTGTATAACTTCCG-----------------------------------------
Ah2             GCTTTGTGTATAACTTCCG-----------------------------------------
Al1             GTCTTGTGTGTATCCTTCG-----------------------------------------
Al2             GTCTTGTGTGTATCCTTCG-----------------------------------------
Al3             GTCTTGTGTGTATCCTTCG-----------------------------------------
Ap1             GCTTTGTGCGTAACTTCCG-----------------------------------------
Ap2             GCTTTGTGCGTAACTTCCG-----------------------------------------
Ap3             GCTTTGTGCGTAACTTCCG-----------------------------------------
Ap4             GCTTTGTGCGTAACTTCCG-----------------------------------------
                      **       *

                                         C2 repeat
At_VARA         GAGGTGTGAGTGTCGCCCATGGGCATCGACACCTTGCGGCTAGGAACTGGAACGAGACGG
At_VARB         ------------------------------------------------------------
Ac1             -----------------------------CAC--TGCGGCTACGA---------------
Ah1             -----------------------------GAC--TGCGGCTTCGA---------------
Ah2             -----------------------------GAC--TGCGGCTTCGA---------------
Al1             -----------------------------CAC--TGCGGCTCCGA---------------
Al2             -----------------------------CAC--TGCGGCTCCGA---------------
Al3             -----------------------------CAC--TGCGGCTCCGA---------------
Ap1             -----------------------------CAC--TGCGGCTACGA---------------
Ap2             -----------------------------CAC--TGCGGCTACGA---------------
Ap3             -----------------------------CAC--TGCGGCTACGA---------------
Ap4             -----------------------------CAC--TGCGGCTACGA---------------

At_VARA         GTAGCAAAGATTTCGAGTAGCACTTCATACTACCGTGGGTTTTTTAAACCTTCCTAGTTT
At_VARB         ------------------------------------------------------------
Ac1             ------------------------------------------------------------
Ah1             ------------------------------------------------------------
Ah2             ------------------------------------------------------------
Al1             ------------------------------------------------------------
Al2             ------------------------------------------------------------
Al3             ------------------------------------------------------------
Ap1             ------------------------------------------------------------
Ap2             ------------------------------------------------------------
Ap3             ------------------------------------------------------------
Ap4             ------------------------------------------------------------
```

```
At_VARA        TGTTGATGTTATTCCGAGAATTAGCAAACCGTAACGAAGATGTTCTTGGCAACCATCTTT
At_VARB        ------------------------------------------------------------
Ac1            ------------------------------------------------------------
Ah1            ------------------------------------------------------------
Ah2            ------------------------------------------------------------
Al1            ------------------------------------------------------------
Al2            ------------------------------------------------------------
Al3            ------------------------------------------------------------
Ap1            ------------------------------------------------------------
Ap2            ------------------------------------------------------------
Ap3            ------------------------------------------------------------
Ap4            ------------------------------------------------------------

At_VARA        TGATGGGAGTCCGGCTGTTCGAAAGCCGGCCAAGGGTGATGAACGAAATGTGAACCCTTG
At_VARB        ------------------------------------------------------------
Ac1            ---------------TATTCGCA-------------------------------------
Ah1            ---------------TATTCGCA-------------------------------------
Ah2            ---------------TATTCGCA-------------------------------------
Al1            ---------------TATTCGCA-------------------------------------
Al2            ---------------TATTCGCA-------------------------------------
Al3            ---------------TATTCGCA-------------------------------------
Ap1            ---------------TATTCGCA-------------------------------------
Ap2            ---------------TATTCGCA-------------------------------------
Ap3            ---------------TATTCGCA-------------------------------------
Ap4            ---------------TATTCGCA-------------------------------------

                                          C3 repeat
At_VARA        TCTCGCCTAGGTTGGATGGGCGTGCTTCGTTGGAAAGCATGGATCCGCCTAGGCTGTCCC
At_VARB        ----GCCTAGGTTGGATGGGCGTGCTTCGTTGGAAAGCATGGATCCGCCTAGGCTGACCC
Ac1            ----GCCTAGGTTGGATGGGCGTGC-------GAAAGCATGGATCCGCCTAGGCTGTCTT
Ah1            ----GCCTAGGTTGGATGGGCGTGC-------GAAAGCATAGATCCGCCTAGGCTGTCCC
Ah2            ----GCCTAGGTTGGATGGGCGTGC-------GAAAGCATAGATCCGCCTAGGCTGTCCC
Al1            ----GCCTAGGTTGGATGGGCGTGC-------GAAAGCATGGATCTGCCTAGGCTGTCTC
Al2            ----GCCTAGGTTGGATGGGCGTGC-------GAAAGCATGGATCTGCCTAGGCTGTCTC
Al3            ----GCCTAGGTTGGATGGGCGTGC-------GAAAGCATGGATCTGCCTAGGCTGTCTC
Ap1            ----GCCTAGGTTGGATGGGCGTGC-------TAAAGCATGGATCCGCCTAGGCTGTCTT
Ap2            ----GCCTAGGTTGGATGGGCGTGC-------TAAAGCATGGATCCGCCTAGGCTGTCTC
Ap3            ----GCCTAGGTTGGATGGGCGTGC-------TAAAGCATGGATCCGCCTTGGCTGTCTT
Ap4            ----GCCTAGGTTGGATGGGCGTGC-------TAAAGCATGGATCCGCCTAGGCTGTCTT
                   *********************        ******* **** **** ***** *

                    P-site
At_VARA        GA-AGTATCTCGCGCTTGTACGGCTTTGGCTCGGATTCGTCCGTCTTCTTTCTTCTTAGC
At_VARB        GAAGGTATCTCGCGCTTGTACGGCTTTGGCTCGGATTCGTCCGTCTTCTTTCTTCTTAGC
Ac1            GACGGTTTTTCGCGCTTGTGCGGCTTTGGCTTGGATTCGTCCGTCTTCTCTCTTCTCAGC
Ah1            GACGGTTTTTCGCGCTTGTGCGGCTTTGGCCTGGATTCGTCCGTCTTCTCTCTTCTCAGC
Ah2            GACGGTTTTTCGCGCTTGTGCGGCTTTGGCCTGGATTCGTCCGTCTTCTCTCTTCTCAGC
Al1            GACGGTTTTTCGCGCTTGTGCGGCTTTGGCTTGGATTCGTCCGTCTTCTCTCTTCTCAGC
Al2            GACGGTTTTTCGCGCTTGTGCGGCTTTGGCTTGGATTCGTCCGTCTTCTCTCTTCTCAGC
Al3            GACGGTTTTTCGCGCTTGTGCGGCTTTGGCTTGGATTCGTCCGTCTTCTCTCTTCTCAGC
Ap1            GACGGTTTTTCGCGCTTGTGCGGCTTTGGCTTGGATTCGTCCGTCTTCTCTCTTCTCAGC
Ap2            GACGGTTTTTCGCGCTTGTGCGGCTTTGGCTTGGATTCGTCCGTCTTCTCTCTTCTCAGC
Ap3            GACGGTTTTTCGCGCTTGTGCGGCTTTGGCTTGGATTCGTCCGTCTTCTCTCTTCTCAGC
Ap4            GACGGTTTTTCGCGCTTGTGCGGCTTTGGCTTGGATTCGTCCGTCTTCTCTCTTCTCAGC
               **   ** * ********** **********  **************** ****** ***
```

```
At_VARA         CGAGTACTTCGGTAGATTAGTTGGAACGATTGATGATTTTGAGTTAATTGAACGTTCGGC
At_VARB         CGAGTACTTCGGTAGATTAGTTGGAACGATTGATGATTTTGAGTTAATTGAACGTTCGGC
Ac1             CGAATATTTCGGAGGAGTCG-GGGAACGATTGATGTTTTTGGGTCAATTGAACGTTCGGC
Ah1             CGAATATTTCGGAGGAGTCG-GGGAACGATTGATGTTTTTGGGTCAATTGAACGTTCGGC
Ah2             CGAATATTTCGGAGGAGTCG-GGGAACGATTGATGTTTTTGGGTCAATTGAACGTTCGGC
Al1             CGAATATTTCGGAGGAGTCG-GGGAACGATTGATGTTTTTGGGTCAATTGAACGTTCGGC
Al2             CGAATATTTCGGAGGAGTCG-GGGAACGATTGATGTTTTTGGGTCAATTGAACGTTCGGC
Al3             CGAATATTTCGGAGGAGTCG-GGGAACGATTGATGTTTTTGGGTCAATTGAACGTTCGGC
Ap1             CGAATATTTCGGAGGAGTCG-GGGAACGATTGATGTTTTTGGGTCAATTGAACGTTCGGC
Ap2             CGAATATTTCGGAGGAGTCG-GGGAACGATTGATGTTTTTGGGTCAATTGAACGTTCGGC
Ap3             CGAATATTTCGGAGGAGTCG-GGGAACGATTGATGTTTTTGGGTCAATTGAACGTTCGGC
Ap4             CGAATATTTCGGAGGAGTCG-GGGAACGATTGATGTTTTTGGGTCAATTGAACGTTCGGC
                *** ** *****  ** * *  ************* ***** ** ***************

At_VARA         GTATGAGTGGTGATCGG
At_VARB         GTATGAGTGGTGATCGG
Ac1             GTATGAGTGGTGATCGG
Ah1             GTATGAGTGGTGATCGG
Ah2             GTATGAGTGGTGATCGG
Al1             GTATGAGTGGTGATCGG
Al2             GTATGAGTGGTGATCGG
Al3             GTATGAGTGGTGATCGG
Ap1             GTATGAGTGGTGATCGG
Ap2             GTATGAGTGGTGATCGG
Ap3             GTATGAGTGGTGATCGG
Ap4             GTATGAGTGGTGATCGG
                *****************
```

Insertion free variants Ac2 and Bo

```
                    A1           A2                      A3              B
Ac2             CTTTTCCGGGCAC-----------------------TTTTCCGGTGGACCATTTTGCCCC
Bo              CTTTTCCGGGCACTTTTCCGGCGAC-----------TTTTCCGGTGGACCATTTTGCCCC
                *************                       ************************

                        P-site & P motif
Ac2             TAAAATCAAATTTTCGCGCTTGTGTGGTCTTGGCCTGGTTTCATCCGTCTTCCAGCTGCT
Bo              TAAAATCAAATTTTCGCGCTTGTGTGGTCTTGCCCTGGTTTCATCCGTCTTCCAGCTGCT
                ******************************** ***************************

                                          Shared
Ac2             CTTTTGATTACATCTCGAGAGTGGTTGGAAAGATTGATGTTAGCGGGGCAA-TGAACGTT
Bo              CTTTTGATTACATCTCGAGAGTGGTTGGAAAGATTGATGTTAGCGGGGCAATTGAACGTG
                *************************************************** ******* 

Ac2             CGGCGTATGAGTGGTGATCGG
Bo              CGGCGTATGAGTGGTGATCGG
                *********************
```
