# Supplementary material for: 45S rDNA Diversity In Natura as One Step towards Ribosomal Heterogeneity in Arabidopsis thaliana
Source: Plants (Basel). 2023 Jul 21;12(14):2722. doi: 10.3390/plants12142722 (PMC10386311; doi:10.3390/plants12142722)
Supplement: Supplementary file 1 [file plants-12-02722-s001.zip › Figure_S4.pdf]

Figure S4: 45S rRNA gene polymorphisms in At66. **(A)** 45S rRNA gene polymorphisms while selecting either the 115 individuals out of 173 sharing the same 5R1 genotype or the 92 individuals out of 173 sharing the same 3R1 genotype. **(B)** 45S rRNA gene polymorphisms for the 6 localities in the order of the altitudinal gradient from 0 to 1900 m (Mas Larrieu, Massane, Mosset, Py1000, Mantet and Py1900). Proportion of individuals of At66 with an alternative allele (defined as >5% within an individual) along the 45S rRNA gene. Vertical lines represent single nucleotide polymorphisms or deletions in the ETS (gray), 18S (red), ITS (green), 5.8S (orange), and 25S (blue) regions along the 45S rRNA gene. Black vertical lines represent insertions.

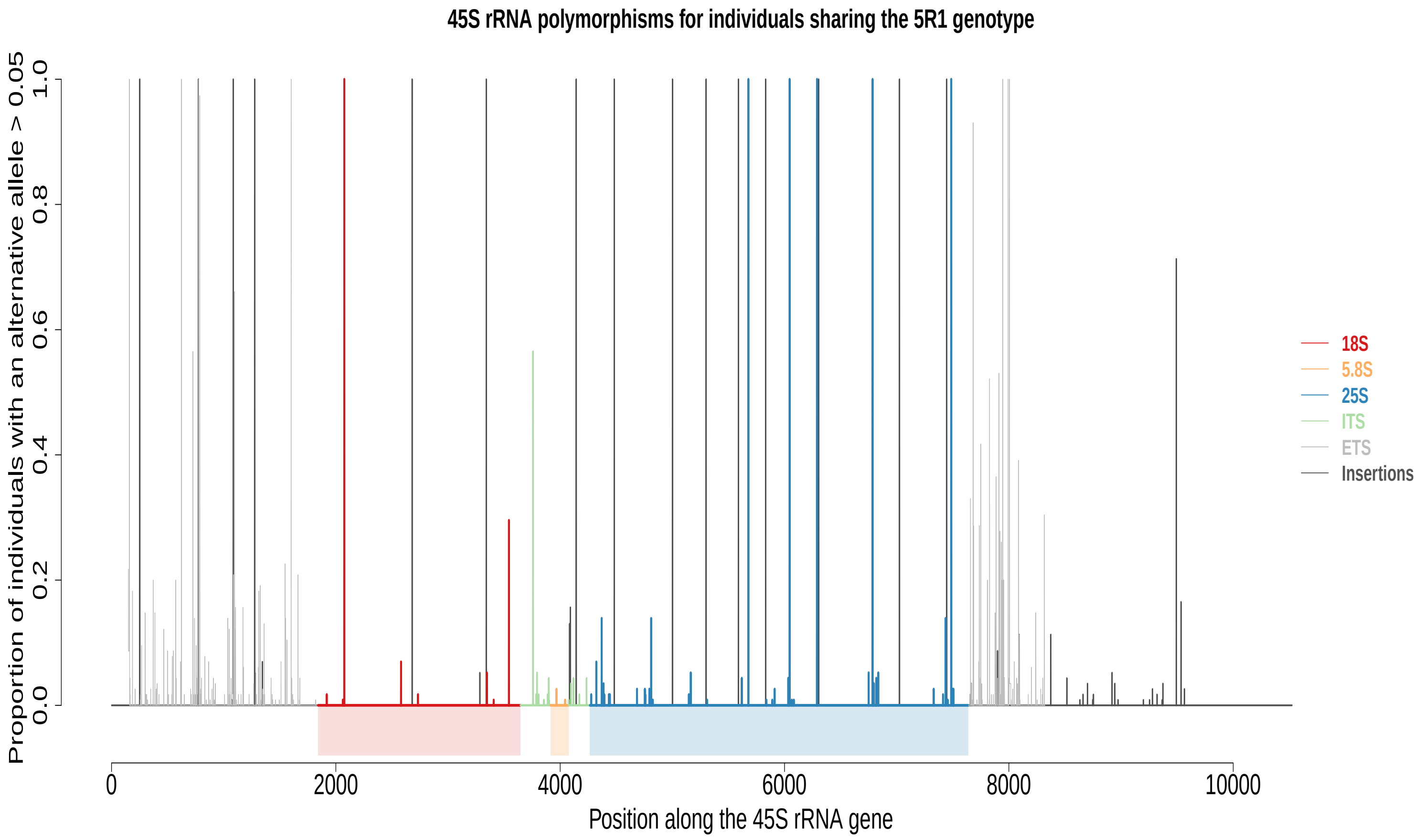
45S rRNA polymorphisms for individuals sharing the 5R1 genotype
Proportion of individuals with an alternative allele > 0.05
0.0
0.2
0.4
0.6
0.8
1.0
18S
5.8S
25S
ITS
ETS
Insertions
0
2000
4000
6000
8000
10000
Position along the 45S rRNA gene

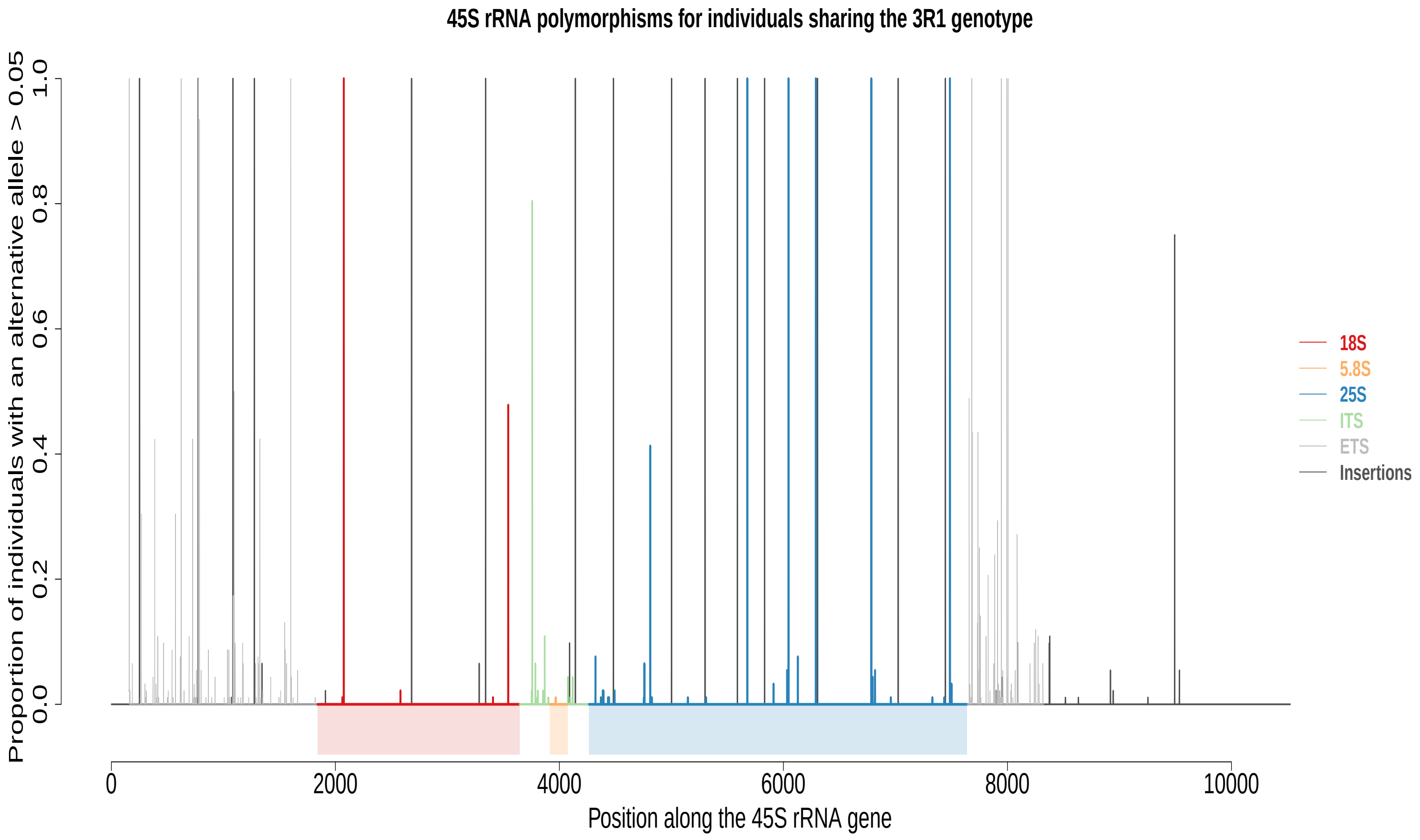
45S rRNA polymorphisms for individuals sharing the 3R1 genotype
Proportion of individuals with an alternative allele > 0.05
0.0
0.2
0.4
0.6
0.8
1.0
18S
5.8S
25S
ITS
ETS
Insertions
0
2000
4000
6000
8000
10000
Position along the 45S rRNA gene

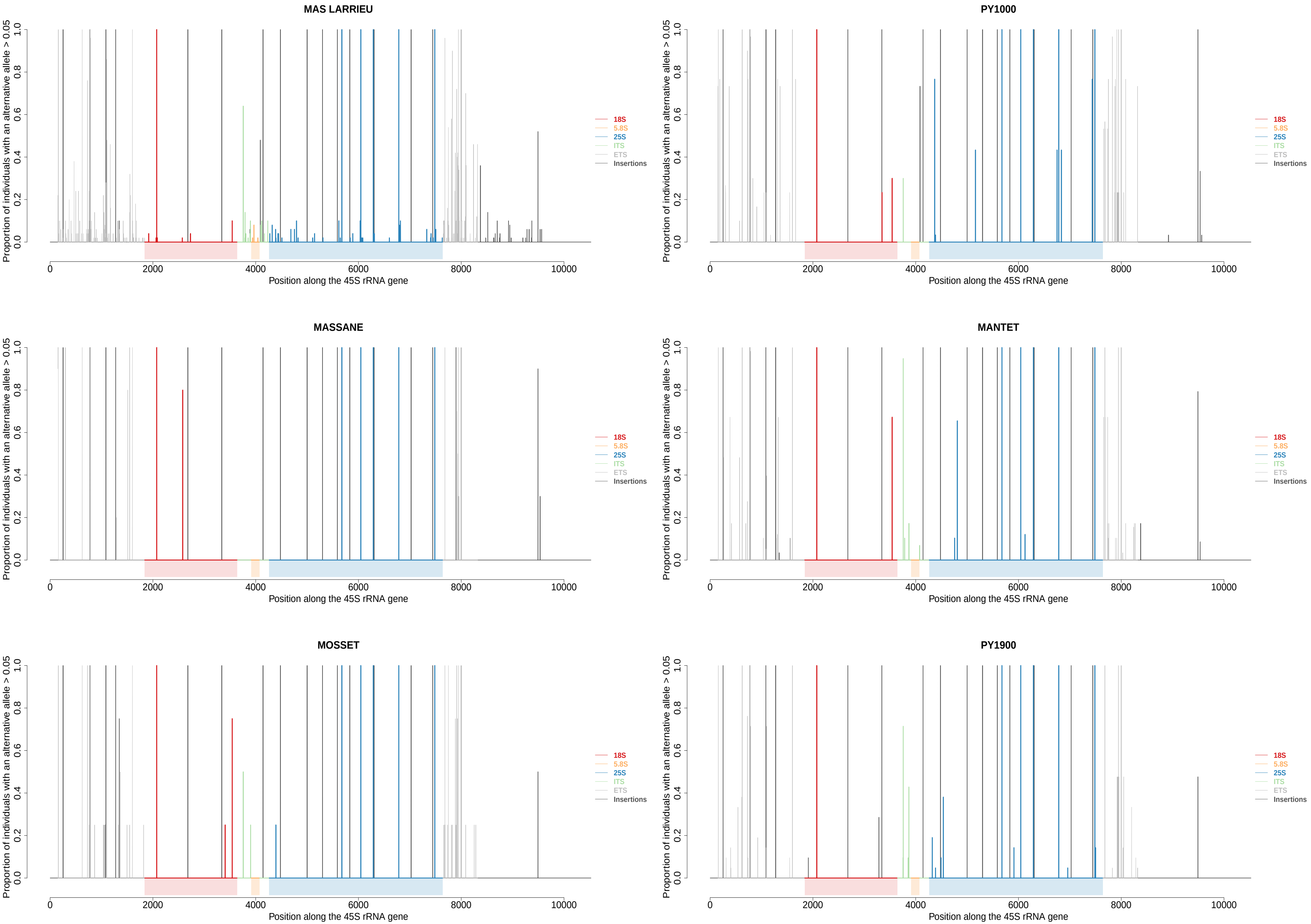
MAS LARRIEU
PY1000
MASSANE
MANTET
MOSSET
PY1900
Proportion of individuals with an alternative allele > 0.05
0.0
0.2
0.4
0.6
0.8
1.0
Position along the 45S rRNA gene
0
2000
4000
6000
8000
10000
18S
5.8S
25S
ITS
ETS
Insertions
