# Supplementary material for: 45S rDNA Diversity In Natura as One Step towards Ribosomal Heterogeneity in Arabidopsis thaliana
Source: Plants (Basel). 2023 Jul 21;12(14):2722. doi: 10.3390/plants12142722 (PMC10386311; doi:10.3390/plants12142722)
Supplement: Supplementary file 1 [file plants-12-02722-s001.zip › Figure_S5.pdf]

Figure S5: GWAS analysis using 45S rDNA copy number variation as a phenotype. Genome-wide association study (GWAS) conducted by GAPIT version 3 using BLINK and FarmCPU multi-loci models, providing both 18S and 25S CNVs as a phenotype, SNP information as a hapmap format and a kinship matrix. For both FarmCPU and BLINK, the first three principal components were fitted as covariate variable to reduce the false positive due to population structure. **(A)** The QQ plots represent the deviation of the observed *p*-values from the null hypothesis: the observed *p*-values for each SNP are sorted from largest to smallest and plotted against expected values from a theoretical χ^2-distribution. When there is no association with the trait, observed values correspond to the expected values and all points are on or near the middle line between the x-axis and the y-axis (red and gray shadowed line highlights the null hypothesis). When observed *p*-values are clearly more significant than expected under the null hypothesis, points will move towards the y-axis. Accounting for population structure and kinship relatedness, the BLINK model performs slightly better than the FarmCPU model. **(B)** Manhattan plots summarizing genome-wide association results along the five chromosomes of *Arabidopsis thaliana*. Each SNP outliers above the genome-wide significance threshold is colored depending on its chromosome location. The genome-wide significance threshold set at -log10(0,01/total number of SNPs) is indicated as a gray line. **(C)** Table recording significant SNP outliers. We only looked for candidate genes using TAIR10 when SNP outliers were either shared by both models and CNVs or one model only but both CNVs.

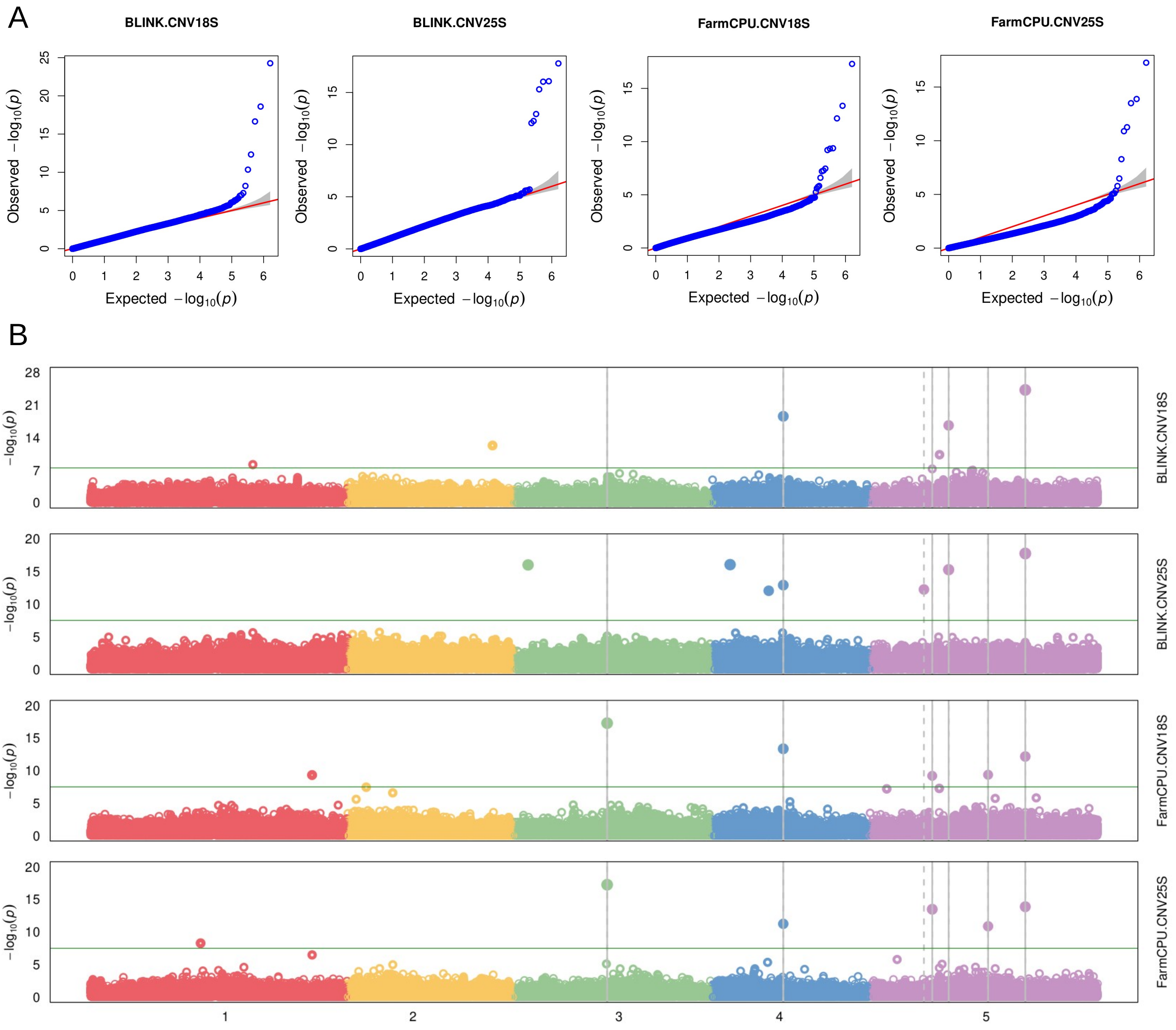

C

Outliers	SNP	Chr	Pos	P.value	MAF	traits	REF allele	alternate allele	TAIR10	Function
151631	S1_13031063	1	13031063	5.235169e-09	0.2456647	FarmCPU.CNV25S				
257106	S1_19212393	1	19212393	6.090339e-09	0.4421965	BLINK.CNV18S				
378779	S1_26223191	1	26223191	4.655183e-10	0.1849711	FarmCPU.CNV18S				
652966	S2_17143203	2	17143203	4.725909e-13	0.2456647	BLINK.CNV18S				
687414	S3_1649985	3	1649985	9.303996e-17	0.06647399	BLINK.CNV25S				
788071	S3_11005534	3	11005534	4.991114e-18	0.2196532	FarmCPU.CNV18S	C	A	AT3G29000 promoter	calcium binding protein 2 EF hands group IV
788071	S3_11005534	3	11005534	5.432796e-18	0.2196532	FarmCPU.CNV25S				
1025237	S4_2088089	4	2088089	8.402371e-17	0.46820809	BLINK.CNV25S				
1102853	S4_6658773	4	6658773	8.615701e-13	0.44797688	BLINK.CNV25S				
1136450	S4_8378198	4	8378198	2.512182e-19	0.2456647	BLINK.CNV18S	T	C	AT4G14600	SFT11, secretory system, Qc-SNARES, plant SFT clade, golgi
1136450	S4_8378198	4	8378198	1.174424e-13	0.24566474	BLINK.CNV25S				
1136450	S4_8378198	4	8378198	4.319601e-14	0.2456647	FarmCPU.CNV18S				
1136450	S4_8378198	4	8378198	5.531750e-12	0.2456647	FarmCPU.CNV25S				
1294251	S5_6433284	5	6433284	5.477141e-13	0.09826590	BLINK.CNV25S				
1308501	S5_7415940	5	7415940	6.234884e-10	0.4710983	FarmCPU.CNV18S				
1308501	S5_7415940	5	7415940	3.202730e-14	0.4710983	FarmCPU.CNV25S				
1321683	S5_8278561	5	8278561	4.521547e-11	0.3786127	BLINK.CNV18S				
1338491	S5_9357427	5	9357427	2.241704e-17	0.1473988	BLINK.CNV18S				
1338491	S5_9357427	5	9357427	5.039445e-16	0.14739884	BLINK.CNV25S				
1432465	S5_14024546	5	14024546	4.206914e-10	0.1618497	FarmCPU.CNV18S	T	C	AT5G26622	Beta-galactosidase related protein, glycosyl hydrolase family
1432465	S5_14024546	5	14024546	1.296281e-11	0.1618497	FarmCPU.CNV25S				
1515507	S5_18423239	5	18423239	5.433961e-25	0.0867052	BLINK.CNV18S	G	C	AT5G45470	unknown function, family of uncharacterized protein DUF594
1515507	S5_18423239	5	18423239	1.640678e-18	0.08670520	BLINK.CNV25S				
1515507	S5_18423239	5	18423239	6.583878e-13	0.0867052	FarmCPU.CNV18S				
1515507	S5_18423239	5	18423239	1.306315e-14	0.0867052	FarmCPU.CNV25S				
