# Supplementary material for: 45S rDNA Diversity In Natura as One Step towards Ribosomal Heterogeneity in Arabidopsis thaliana
Source: Plants (Basel). 2023 Jul 21;12(14):2722. doi: 10.3390/plants12142722 (PMC10386311; doi:10.3390/plants12142722)
Supplement: Supplementary file 1 [file plants-12-02722-s001.zip › Figure_S6.pdf]

Figure S6: 5'ETS variation in additional species of Brassicaceae Lineage I, Clade A.

(A) 5'ETS genotyping in Cv, *Chrysochamela velutina*, Dm, *Descurainia millefolia*, Cp, *Cardamine parviflora*, Bs, *Boechera stricta*, Lc, *Lepidum campestre* compared to At, *Arabidopsis thaliana, Rs, Raphanus sativus* and Br-r, *Brassica rapa ssp rapa.* * uncloned amplified PCR products. Sizes in base pairs are labeled alongside the lane of the $1kb^+$ DNA ladder. **(B)** Schematic representation of variants containing an insertion splitting away the conserved U3 snoRNP binding site $A^{123}B$ from the primary processing site P. **(C)** Schematic representation of insertion free variants. Dotted lines indicate deletions and a number of base pairs between brackets indicates the size of an inserted sequence without any homology with known D or C repeats. **(D)** MUltiple Sequence Comparison by Log- Expectation (MUSCLE) alignments of 5'ETS insertion free variants. *Brassica oleracea (*Bo*)*, *Arabidopsis cebennensis* (Ac2) and *Lepidum campestre* (Lc2). **(E)** Fasta sequences of 5'ETS variants.

Brassicaceae Lineage I, Clade A, Camelineae

Cv1 and Cv2 are supported by two independent clones whereas Cv3 sequence was only cloned once for *Chrysochamela velutina*.

Brassicaceae Lineage I, Clade A, Boechereae

Bs is the 100% identity sequence supported by two independent clones for *Boechera stricta.*

Brassicaceae Lineage I, Clade A, Cardamineae

Cp is the common sequence shared by two independent clones for *Cardamine parviflora.*

Brassicaceae Lineage I, Clade A, Descurainieae

Dm is the 100% identity sequence shared by three independent clones for *Descurainia millefolia*.

Brassicaceae Lineage I, Clade A, Lepidieae

Lc1 and Lc2 are both only supported by one clone for *Lepidium campestre*.

Supplementary result description

Longest variants were found in *Chrysochamela velutina* and *Lepidum campestre*, intermediate variants were identified in *Chrysochamela velutina, Descurainia millefolia, Cardamine parviflora, Boechera stricta*, insertion free variant was abundant in *Lepidum campestre* and only faintly detectable in *Boechera stricta* and *Chrysochamela velutina*. In addition to the deletion of the D1a repeat identified in the *Arabidopsis* genus, the major variant identified in *Boechera stricta* has a truncated version of D1b, complete loss of D2a, partial loss of D12 but still D2b and a truncated C-like repeat. When present in Brassicaceae species, the insertion either retains partially some homologies with some D and C repeats (*Boechera stricta*, *Lepidum campestre*, *Chrysochamela velutina*) and/or interrupt them by insertions (*Chrysochamela velutina*) or share no homology with any of them (*Cardamine parviflora, Descurainia millefolia).* Illustrating some of these statements for C*hrysochamela velutina*, we identified two major variants of 1912 bp (Cv1) and 552 bp (Cv3) while genotyping and an additional rare variant of 946 bp (Cv2) after cloning and sequencing. Suprisingly, all Cv variants have an insertion separating D repeats from the C like repeat, the length of this insertion vary from 72 bp in Cv1-2 to 525 bp in Cv3. Cv1-2 variants have a truncated D1b repeat while Cv3 retained both D1b and D2a. Contrary to Cv1, Cv2-3 retained the additional third occurrence of the C3 repeat but an insertion of 51 bp separate it from the P site. There is an additional insertion separating the C-like repeat from either the primary processing site (Cv1, 110 bp) or the last C3 repeat occurrence (Cv2, 459 bp ; Cv3, 815 bp).

(A) 5'ETS genotyping in additional species of Brassicaceae Lineage I, Clade A

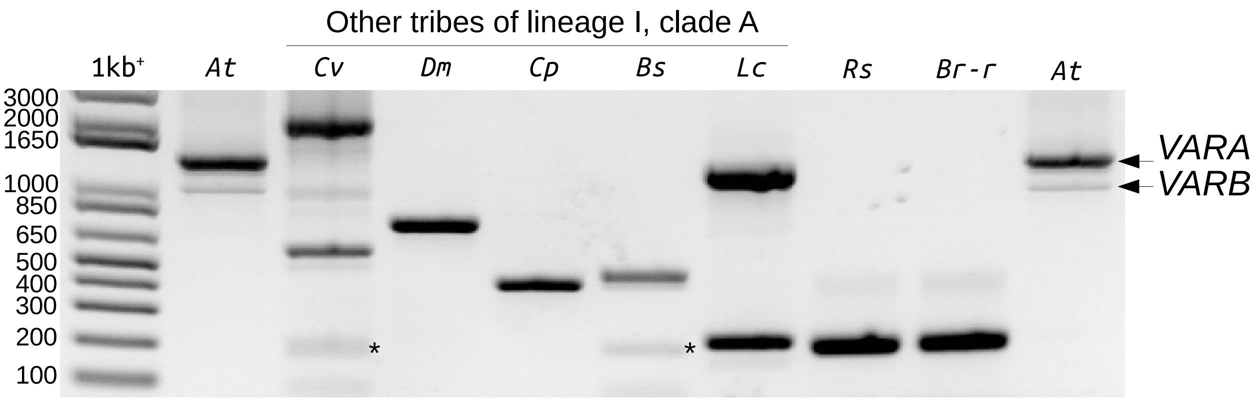

(B) Schedules of variants containing an insertion splitting away the conserved U3 snoRNP binding site A123B from the primary processing site P

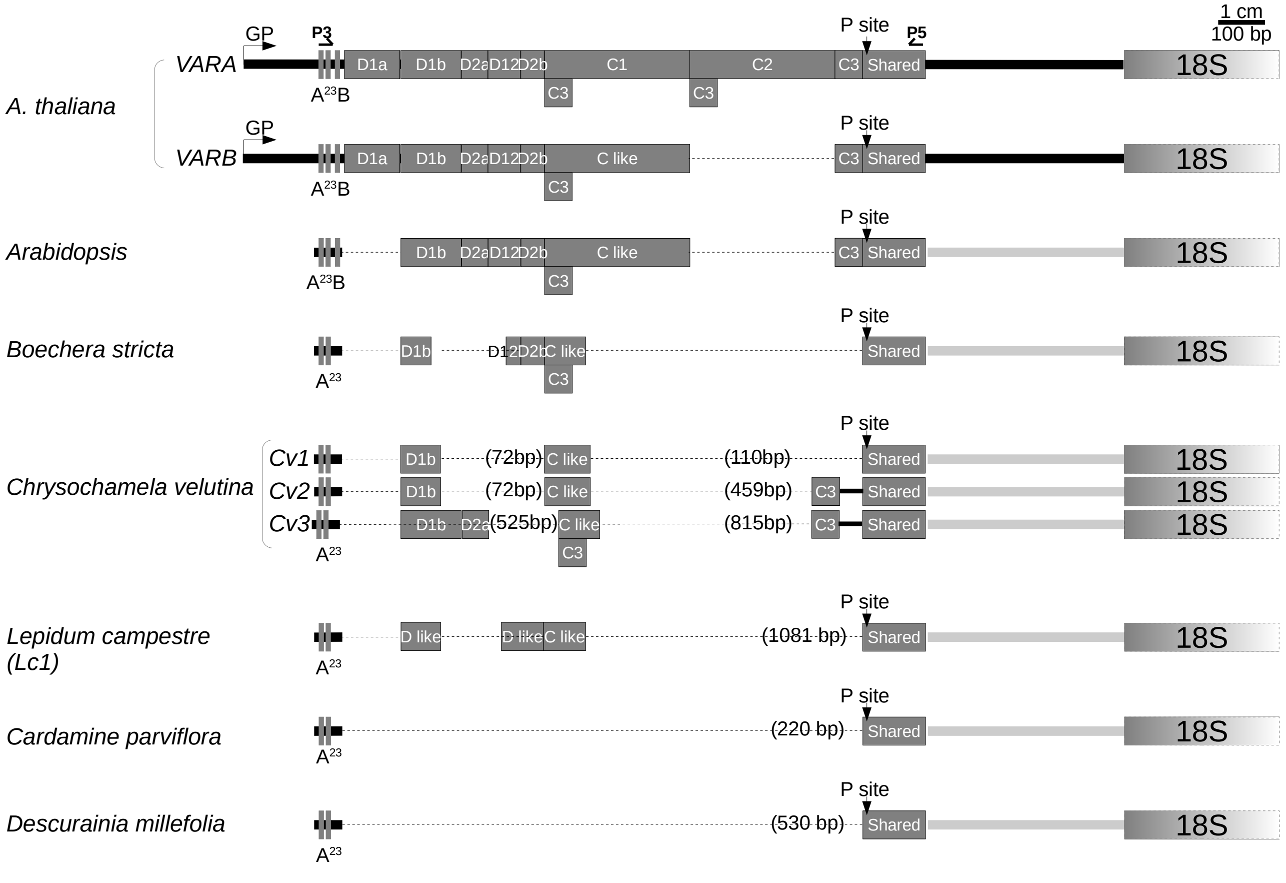

(C) Schedules of insertion free variants

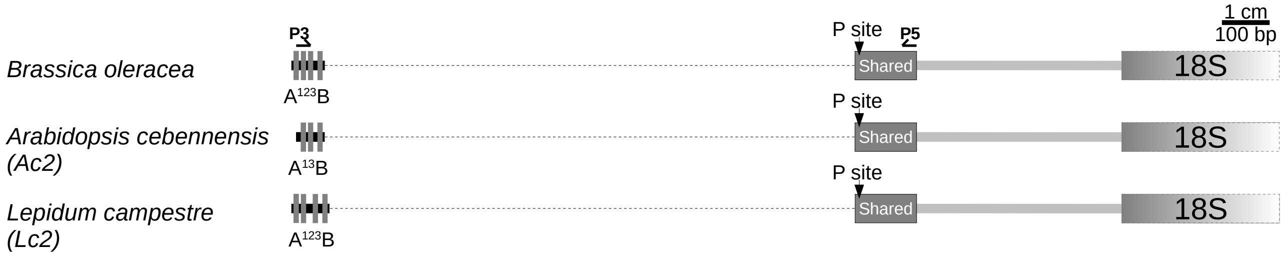

(D) MUSCLE alignment of 5'ETS insertion free variants

```
                    A1          A2                     A3             B
Ac2             CTTTTCCGGGCAC-----------------------TTTTCCGGTGGACCATTTTGCCCC
Lc2             CTTTTCCGGGCACTTTTCCGGCGACTTTCCGGCGAGTTTTCCGGTGGTTCATTTTCTTCC
Bo              CTTTTCCGGGCACTTTTCCGGCGAC-----------TTTTCCGGTGGACCATTTTGCCCC
                *************                       ***********  ******   **

                       P-site & P motif
Ac2             TAAAATCAAATTTTCGCGCTTGTGTGGTCTTGGCCTGGTTTCATCCGTCTTCCAGCTGCT
Lc2             C---TTTATTTTTTCGCGCTTGTGCGGCTTTGGACTGGTTTCATTTGTCTTCCTATTGCA
Bo              TAAAATCAAATTTTCGCGCTTGTGTGGTCTTGCCCTGGTTTCATCCGTCTTCCAGCTGCT
                     * *  ************** **  ***  **********  *******   ***

Ac2             CTTTTGATTACATCTCGAGAGTGGTTGGAAAGATTGATGTTAGCGGGGCAA-TGAACGTT
Lc2             TTTTCGATTATATCGAAAGAGTGGTTGGAAAGATTGATGTTAGCGGGCCAACTGAACGTT
Bo              CTTTTGATTACATCTCGAGAGTGGTTGGAAAGATTGATGTTAGCGGGGCAATTGAACGTG
                 *** ***** ***   ****************************** *** *******

Ac2             CGGCGTATGAGTGGTGATCGG
Lc2             CGGCGTATGAGTGGTGATCGG
Bo              CGGCGTATGAGTGGTGATCGG
                *********************
```

(E) Fasta sequences of 5'ETS variants

```
>Cv1
CTTTTCCGGGCACTTTTCCGGCGGTCGGTTTTTTTGCCTTTTCGGATTTTTTCCGGATTAATCGACGAAAGA
TGGTGAATGGCCGAGGAGAATGGGCGTGTCATGCATGGGCTGACATGGATTCTCCGAGGCCGAGGGTG
GAGGTATGTTCGGATGGGCCCCACGACATTACCTGATGTGCCCATGCGCATCGTTACACCCCAAGCCGAA
CTGGATGGGCGTGCTCTGTTGGGGAGCATGGATCCAGTTTTTGGCCGTCCCGGGGGGATCAAGGTGGCG
ATGTCGCCCATGGGCAACGATCACCTTGTGGAAGGGTTCGGTAACGGAAATGGTTGCGGTAATGAAAAA
AATGGAAGCTGGCTCCCGTGTTCGACAATCATCGATGTTTCGTCAATGGACATCGAACATTCTTAGGATTTG
TTTCTCGCGCTTGTGCGGCTTTGGCTTGGTTCCGTCCGTCTTCACTTTGCTCGTCCGAATATTTCGGAAGG
GTGGGGGGAATGATTGGTGGTTTTGGGTCAATTGAACGTTCGGCGTATGAGTGGTGATCGG
>Cv2
CTTTTCCGGGCACTTTTCCGGCGGTCGGTTTTTTTGCCTTTTCGGATTTTTTCCGGATTAATCGACGAAAGA
TGGTGAATGGCCGAGGAGAATGGGCGTGTCATGCATGGGCTCACATGGATTCTCCGAGGCCGAGGGTGG
AGGTATGTTCGGATGGGCCCCACGACATTACCTGATGTGCCCATGCGCATCGTTACACCCCAAGCCGAAC
TGGATGGGCGTGCTCTGTTGGGGAGCATGGATCCAGTTTTTGGCCGTCCCGTGGGGATCAAGGTGGCGA
TGTCGCCCATGGGCAACGATCACCTTGTGGAAGGGTTCGGTAACGGGAATGGTTGCGGTAATGAAAAAA
ATGGAAGCTGGCTCCCGTGTTCGACAATCATCGATGTTTCGTCAATGTAATCGATCCGGTCGTTAGCCGATG
TTGGAGTAATTGCCGTAGCCTTCGACGTTTCTGTTCCTGGGAACCATCGTTTGATGGGAGTCCGGGTTTTG
CAAAACCGGCCAAGGGTAATGAACGTATTGTGAACCCTTGTCGCCTAGTCGGATGGGCGTGCTTAGGTAG
CATGGATCCGGTTAAGCTTGCTGGCCCGTGAGGGGTCTCTCTTCAATCGGACTTCAGGCGTGCCGTACTG
CGCACACAAGCATGTTGGACGTTGATTCGGAGATACCTACCGCAGCTCTCATAGATATTGCAGTCTATCGCC
TAGGTCGGATGGGCGTGCTTCGATGGAAAAGCATGGATCCGCCTAGGCTGTCCCGACGGTTACACAAGAT
GACGGTGTCTCCCATGGACATCGAACATTCTTAGGATTTGTTTCTCGCGCTTGTGCGGCTTTGGCTTGGTT
CCGTCCGTCTTCACTTTGCTCGTCCGAATATTTCGGAAGGGTGGGGGGAATGATTGGTGGTTTTGGGTCAA
TTGAACGTTCGGCGTATGAGTGGTGATCGG
>Cv3
CTTTTCCGGGCACTTTTCCGGCGGTCGGTTTTTTTGCCTTTTCGGATTTTTTCCGGATTAATCGACGAAAGA
TGGTGAATGGCCGAGGAGAATGGGCGTGTCATGCATGGGCTGACATGGATTCTCCGAGGCCGAGGGTG
GAGGTATGTTCGGATGGGCCCCACGACATTACCTGATGTGCCCATGCGCATCGTTACACCCCAAGCCTAAC
TGGATGGGCGTGCTCTGTTGGGGAGCATGGATCCAGTTTTTGGCCGTCCCGGGGGGATCAAGGTGGCGA
TGTCGCCCATGGGCAACGATCACCTTGTGGAAGGGTTCGGTAACGAAATGGTTGCGGTAATGAAAGAAAT
GGAAGCTGGCTCCCGTGTTCGACAATCATCGATGTTTCGTCAATGTAATCGATCCGGTCGTTAGCCGATGTT
GGAGTAATTGCCGTAGCCTTCGTCGTTGCTGAACCTGGGAACCATCGTTTGATGGGAGTCCGGACTTTCC
AAAGCCGGCCAAGGGTAATGAACGAATTGTGAACCCTTGTTTATCGCCAAGTCGGATGGGCGTGCTTTGT
TGGAGAGCATGGATCCGGTTAGGCTGTCCGATGATTACTTAGTTGCGCGTGGTATTGCATGTCGAAGTGGC
TGGCCCGCGAGGTGTCTTCCTTCAATCTCCCTTCAGACGTTCTGTTTCACGTACGTTTTGTTGGGAGTTGAT
CCGAGGATAACTACCGCAGCTCTCCTAGATATTGCACTCTATCGCCTAGGTTGGATGGGCGTGCTTTGTTG
GGAAGCATGGATCCGCCTAGGCCGTCCCGGGGGAAGCAAGGTGGCGATGTCGCCCATGGGCAAAGATC
```

ACCTTGTGGCTAGGTTCAGTAACGGGAAAGGTTGCGGTAATGAAGAAAATGGAAGCTGGCTACCGTGTTC
GACAATCATCGATGTTTCGTCAATGTAATCGATCCGGTCGTTAGCCGATGTTGGAGAAATTGCCGTAGCCTT
CGTCGTTTCTGTTCCTGGGAACCATCGTTTGATGGGAGTCCGGACTTTCCAAAGCCGGCCAAGGGTAATG
AACGAATTGTGAACCCTTGTTTATCGCCAAGTCGGATGGGCGTGCTTTATTGGAGAGCATGGATCCGGTCA
GGCTGTCCGATGGTTACTTAGTTGCGCGTGGGGATTTTCATATCGCCTAGGTTGGATGGGCGTGCTTTGTT
GGGAAGCATGGATCCGCCTAGGCCGTCCCGGGGGAAGCAAGGTGGCGATGTCGCCCATGGGCAACGAT
CACCTTGTGGCTAGGTTCAGTAACGGAAAGGGTTGCGGTAATGAAGAAAATTGAAGCTTGCTCCCGTGTT
CGACAATCATCGATGTTTCGTCAATGTAATCGATCCGGTCGTTAGCCGATGTTGGAGTAATTGCCGTAGCCT
TCGACGTTTCTGTTCCTGGGAACCATCGTTTGATGGGAGTCCGGGTTTTGCAAAACCGGCCAAGGGTAAT
GAACGTATTGTGAACCCTTGTCGCCTAGTCGGATGGGCGTGCTTAGGTAGCATGGATCCGGTTAAGCTTGC
TGGCCCGTGAGGGGTCTCTCTTCAATCGGACTTCAGGCGTGCCGTACTGCGCACACAAGCATGTTGGACG
TTGATTCGGAGATACCTACCGCAGCTCTCATAGATATTGCAGTCTATCGCCTAGGTCGGATGGGCGTGCTTC
GATGGAAAAGCATGGATCCGCCTAGGCTGTCCCGACGGTTACACAGGATGACGGTGTCTCCCATGGACAT
CGGACATTCTTAGGATTTGTTTCTCGCGCTTGTGCGGCTTTGGCTTGGTTCCGTCCGTCTTCACTTTACTCG
TCCGAATATTTCGGAAGGGTGGGGGGAATGATTGGTGGTTTTGGGTCAATTGAACGTTCGGCGTATGAGT
GGTGATCGG
>Bs
CTTTTCCGGGCACTTTTCCGGCGGTCGGTTTTGTTGCCTTTTCTGGATTTTTTCGGCATTTCTCGACGAAAG
ATGGGGAGTGGCCGAGGATGCATGTGCGGACATGGATTCTTCGAGGCTTTGGGTGGCGGTACGTTCGGA
GGGCAAACTAAATACCGAGATGTCCCCATGGTCACCGTTTCCACCCGTGCATAGGTCGGATGGGCGTGCT
TCGATGGAAAGCACGGATCCGCCTAGGTTGCCCCGACGGTTAAACAAGATGACGGTGTCTCCCATGGGCA
TCGGTCAAATCTTTTGGCTAGTTTTTCGCGCTTGTGCGGCTTTGGCTTGGATTCGTCCGTCTTCTCTCAGCT
CAACCAAATACTTTGGAAGAGCTGTGGGAACGATTGAAGTTTTTGGGTCAACTGAACGTTCGGCGTAGAG
TGGTGATCGG
>Cp
CTTTTCCGGGCACTTTTCCGGCGAGCGGTTTTGATGACTTTCTCTGATTTTTTCAGCATGTCTCCACGAAAG
ATGGTGATATAGCGGCCTAGTCTGATAGCGAGCTTGTAGATTCCAAAGGTGCATGTCCGAGTGAAATGATG
GAGCTGGCCCGGGAGTGGGGTAGAGGAGCCTTAACGGGTAAACCACTACTTTCGTCCCCCGCGGCGAA
GTTGTTTCCGTGACATGCATTGGAGGAAATTGTTTGCAATCGATTTGGGCTAGTGTTTCGCGCTTGTGCGG
CTTTGGCTTGGATTCGGCTGTCTTCTCTCGCTCTTTTGATTTCCTCGGAAGGGTTGTCGGAAAGATTGTCGT
CTTCGGGTCAACTGAACGTTCGGCGTATGAGTGGTGATCGG
>Dm
CTTTTCCGGGCACTTTTCCGGCGGACGGTTTTAGAGGATTTTTTGGGTTTTCGACCGTGCTGATGGGTTTG
AAAATGAAAGCTACGGAATTCCACGACTGTAAGTCGACATAGTAGCCGTTTGGGTCCGTCTAAGCTTGGAG
GCAAATAATTGGTCGTTCGATTAGCCGTAAAGATTTGGTGCGGATCGTTTCTGGGTGTCGTAGTGACAGCC
TCTGGCGTAGGGCCAATGTGAACTCTAACTGACATTATGTTAATATTGTTCACAGCCCAGCTACGCGATCAC
TAATACCCCCAGAGTTTCGAGCCGTGGCGCCTGCTTTCGGTGAAAACCGATGTGCGTTTGCTTTCAGGTTT
CCATGTCCTAGCAGTAGGTATTATGATATCGGATAAAGGATGGCCCTGTTCCGCAGGCGCAATTGTTCCGAT
TCGATATCATAACCGAAAATGGGAACTGGGTACGATGGTCCTATATTGGTGAACCGTTTGCTTACTTTTGCG
GGCGTCAATAGTTGAGTTTTCTTTCCCGAAGGCTTATATTCCCTTCCCACGGCCCCTATTTGTGTGTCCCTT
GGTGTTTTCGCGCTTGTGCGGCTTTGGCTTGGTTATGTCCGTCTTCTCTCTGCTCATCCAAATCCTTGGACG
AGTGGTGGGAATGATTGATGTTTTCGGGTCAATTGAACGTTCGGCGTATGAGTGGTGATCGG
>Lc1
CTTTTCCGGGCACTTTTCCGGCGGCCGGTTTAAGCGATTTTTGGCTCTTTCTGGGGGGGATTATGACGAAA
GATGGTGTTTCCGTTGAGCTTGTGGTTGGCCTAGAGGAATGGGCGTGCTAGCGTGGGAAGCATGGATTCT
TCTAGGCTCCAACGGGTGGCGTTTTTTCAGATCGAGGTGTCCCCATGGCATCGAACCACCCTTTCGCCCAG
GCGGCTGGGCGTGCTTCGTTGGAAAGTACGGATCCGTCCTGGGCTGTCCCGATCGGAAAAGCAGGGGC
GTTTCGCCCATGGAAATCGTTCACCCTGCGTCGGGAACTGATGCGGAACTGGGCTCTAGGCGGCGATTTA
AAGTGGCATATGAAAGCGGGTTCGAGAAAAATCTGATCTCTTCGGTAGTTAGCTCACACTGTCCGGGTGAA
CCAACAAAACGAGATTCGATTCGATTGTCCCCGAACGATTTTGCTCAACTTAAAATCGTCGTTGATATCCCT
CTCCGCAGATGTTCCTGGCAACCATCCCGCGATGGAAGTCCGGCCATTTGAAGGGTCGGCCGTAGGTGCT
GATGGGCGACGGTGCCGAAGGAAATTTGCCAACGGGTTCGGAAGCGATCACTCTGTCCGAAGTCTCTTT
GGTTTGGCGAAGAATATCCAATTATTGCGGTCGAGCCATTTGCATAATCCTGACCACAGTGGACGCTAGTA
ACCTTTTGATGTCGAAAATGGATGGCACTGGCATGCGTAACGCGATAAGCTTTCTTAGAGATTACGTCCCC
AGGTGCAGTTGTTCCCTCTTAATGATGTCATCGGGCGAAGGCCGAAATGTGCGACGGTTTGTGGGTTTGG
TGAGATCTCAGTTTTGGTGTTTCCATAGGCAGATCATCGAGAACAGTAAGATCTTTGTGACCGTAGACGTTC
TCCAAAGGCGATGACCGAAGTATCAGCCTGATGATGTAGCTACGTTACTGCCCGGGCGGATGGGCGTGCT
TTTATTAATTTATGTGGTGGGAAAGCACGGATCCGCCTAGGCTGTGTCCCGACGGGTTAAAGAAGGTGGT
GGTGTCATCCCATCGGCATCGTTCCATCTTTTTCGTGCTTGTGCGGCTTTCGGCTTGGTTACGTCCGTCTTC
CCGCTGCTCAACCGAGTTCAACCTCGGAAGGGTTGCGAGAAAGATTGATGTTTTCGGGTCAACTGAACGT
TCGGCGTATGAGTGGTGATCGG

>Lc2
CTTTTCCGGGCACTTTTCCGGCGACTTTCCGGCGAGTTTTCCGGTGGTTCATTTTCTTCCCTTTATTTTTTCG
CGCTTGTGCGGCTTTGGACTGGTTTCATTTGTCTTCCTATTGCATTTTCGATTATATCGAAAGAGTGGTTGG
AAAGATTGATGTTAGCGGGCCAACTGAACGTTCGGCGTATGAGTGGTGATCGG
