# Supplementary material for: 45S rDNA Diversity In Natura as One Step towards Ribosomal Heterogeneity in Arabidopsis thaliana
Source: Plants (Basel). 2023 Jul 21;12(14):2722. doi: 10.3390/plants12142722 (PMC10386311; doi:10.3390/plants12142722)
Supplement: Supplementary file 1 [file plants-12-02722-s001.zip › Figure_S7.pdf]

Figure S7: Model-averaging approach testing the effects of altitude and bioclimatic variables on 18S CNV. **(A)** cluster dendrogram illustrating the cutoff and the selection of 6 among 19 bioclimatic variables (bio3, bio4, bio8, bio9, bio13 and bio15) downloaded from WorlClim layers. **(B)** plot illustrating that the best model provided with an R2 = 0.22 for 18S CNV didn't support any effect as confidence interval (CI) was overlapping zero with p-value > 0.05 for all variables tested. (C) Q-Q plot. (D) estimates (CI 95%) for each variable tested.

Supplementary materials and methods: We downloaded 19 bioclimatic layers from WorldClim [50,51] and selected the least correlated ones (Person correlation coefficient <0.75) thanks to the R package "ENMWizard" [52] and kept six variables: bio3, isothermality (bio2/bio7) (×100), bio4, temperature seasonality (standard deviation ×100), bio8, mean temperature of wettest quarter, bio9, mean temperature of driest quarter, bio13, precipitation of wettest month and bio15, precipitation seasonality (coefficient of variation) for further analyses. All statistical analyses were performed using the R4.2.2 software [53] in the Rstudio environment [54]. All linear mixed-effects models were implemented in R using the lme4 package [55]. Model averaging was performed using the R package 'MuMIn' [56].

A

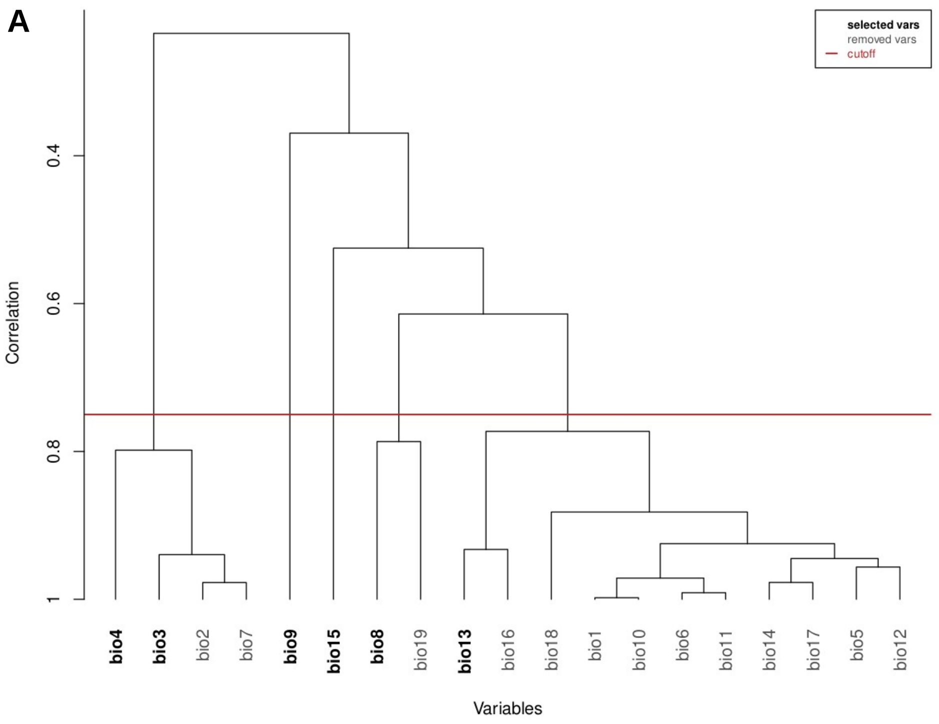

B

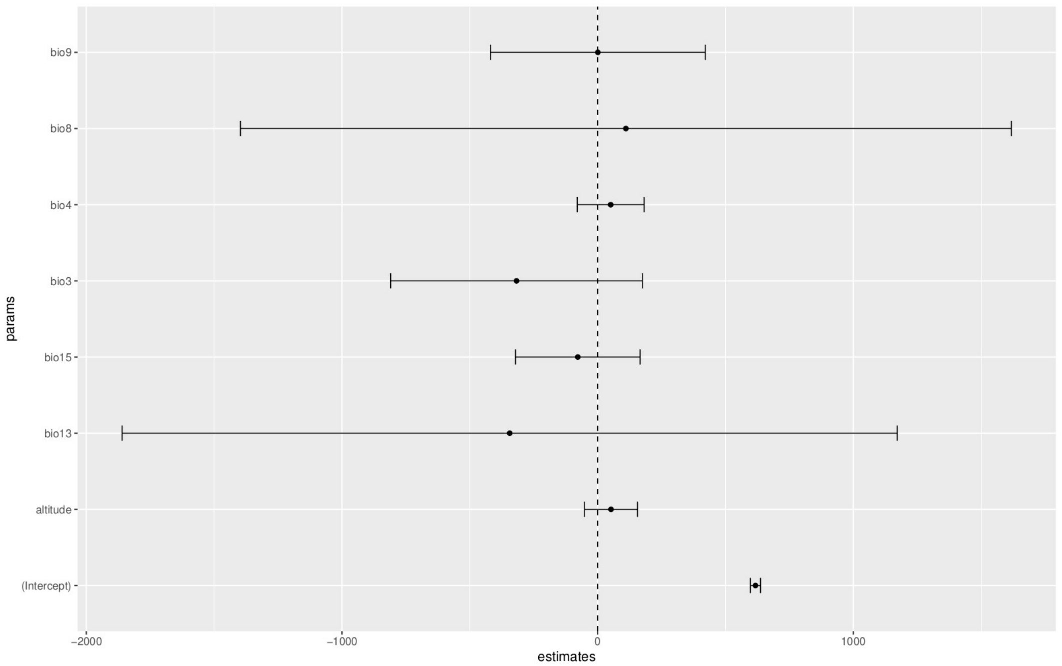

C

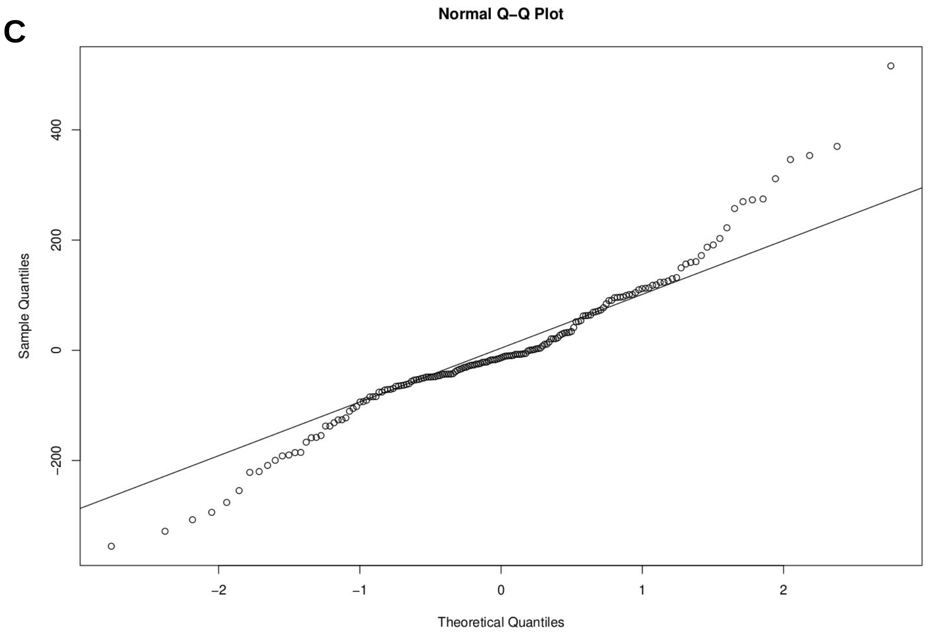

D

Estimates of model-averaging approach testing the effects of altitude and bioclimatic variables on 18S CNV.
R^2 is part of the variance explained by the best model.
There is no significant interactions as CI is overlapping zero and p-value >0,05.

	Estimates (CI 95%)
coefficient of determination	R^2=0,22
intercept	617,36 (597,52 ; 637,20)
altitude	52,29 (-51,52 ; 156,09)
bio3	-317,21 (-809,67 ; 175,25)
bio4	51,13 (-79,66 ; 181,92)
bio8	110,53 (-1397,19 ; 1618,26)
bio9	0,89 (-419,20 ; 420,99)
bio13	-344,00 (-1859,99 ; 1171,98)
bio15	-77,62 (-321,44 ; 166,20)
