# Supplementary material for: 45S rDNA Diversity In Natura as One Step towards Ribosomal Heterogeneity in Arabidopsis thaliana
Source: Plants (Basel). 2023 Jul 21;12(14):2722. doi: 10.3390/plants12142722 (PMC10386311; doi:10.3390/plants12142722)
Supplement: Supplementary file 1 [file plants-12-02722-s001.zip › Figure_S8.pdf]

Figure S8: Fasta sequence of the 45S rDNA of 10524 bp used as a reference to estimate 18S and 25S copy number variation and rDNA variants. This reference includes the most abundant 5' and 3'ETS variants identified in the reference Col-0 and At66, *VARA* (150-1409) and *VAR1* (7852-8322). Annotation of 18S, ITS1, 5.8S, ITS2 and 25S coordinates respectively 1841-3644, 3645-3912, 3913-4073, 4074-4263, 4264-7639.

>45S rDNA unit
TATATAGGGGGGTGGGTGTTGAGGGAGTCTGGGCAGTCCGTGGGGAACCCCCTTTTT
CGGTTCGGACTTGGGTAGCGATCGAGGGATGGTATCGGATATCGGCACGAGGAATGA
CCGACCGTCCGGCCGCCGGGATTTTCGCCGGAAAACTTTTCCGGGCACTTTTCCGGC
GATCGGTTTTGTTGCCTTTTTCCGAGTTTTCTCAGCAGTTCTCGGACAAAAACTGCTG
AATCGTCGAGGAGAATGGGCTTGCTTGCGTGGGCTGCCATTAGTTCTTCGAGGCGTT
AGGGTGGCGGCGGTATAAAAGTGTCGGAGTTTTTTCAGCAGTTCTCGGACAAAAATTG
CTGAGTGGCCGAGAAGAATGGGCGTGTCATGCGTGGGCTGACATGGATTCTTCGAGG
CCTAGGGGTGGCGGTATATAACTTGTTCGCATGATATTACCGAGATGTCCCCACGGGC
ATCTTTTCACCTCGTCGCCGAAGAGAATGGGCGTGTCATGGCATGGGCTGACATGGAT
TCTCCTAGGCCGTTTGGGTGGCGGTATAGTCGTCTTGCGCACGAAATACCGAGATGTC
CCCATGGGCATCGATTCCACCCGCCTAGGTTGGATGGGCGTGCTTCGTCGGAAAGCA
TGGATCCGCCTAGGCTGTCCCGAGTGTGAGCGAGGTGTGAGTGTCGCCCATGGGCA
TCGACACCTTGCGGCTAGGAACTGGAACGAGACGGGTGGCAAAGATTTCGAGTAGCA
CTTCATACTACCGTGGGTTTTTTAAACCTTCCGAGTTTTGTTGATGTTATTCCGAGAATT
AGCAAACCGTAACGAAGATGTTCTTGGCAACCATCTTTTGATGGGAGTCCGGCTGTTC
GATAGCCGGCCAAGGGTGATGAACGAAATGTGAACCCTTGTCTCGCCTAGGTTGGAT
GGGCGTGCTTCGTTGGAAAGCATGGATCCGCCTAGGCTGTCCCGAGTGTGAGCGAG
GTGTGAGTGTCGCCCATGGGCATCGACACCTTGCGGCTAGGAACTGGAACGAGACG
GGTAGCAAAGATTTCGAGTAGCACTTCATACTACCGTGGGTTTTTTAAACCTTCCTAGT
TTTGTTGATGTTATTCCGAGAATTAGCAAACCGTAACGAAGATGTTCTTGGCAACCATC
TTTTGATGGGAGTCCGGCTGTTCGAAAGCCGGCCAAGGGTGATGAACGAAATGTGAA
CCCTTGTCTCGCCTAGGTTGGATGGGCGTGCTTCGTTGGAAAGCATGGATCCGCCTA
GGCTGTCCCGAAGTATCTCGCGCTTGTACGGCTTTGGCTCGGATTCGTCCGTCTTCTT
TCTTCTTAGCCGAGTACTTCGGTAGATTAGTTGGAACGATTGATGATTTTGAGTTAATTG
AACGTTCGGCGTATGAGTGGTGATCGGATAGCTAGTGTTCGTAGGCTCCATGCTCGCG
CATCGAACTACCTACCACCTATCCTTCTCAGTTAATTCACGGGCGATGTTACGCTCGAT
GATGAGTTCCGGGGCCTGTGTTTCGTACCTAATTTGAAGGAATTGTTGAGTTTGGTTTA
CACCTTTGCCCGCGGCTTCTCCTTCGTGGGGAAGTCGTGGGCTCAAACATCGGCGCT
TGTTCACCTCTCGTCATCGCATTTGTTGCCTTGCTCGCATTGGTGAATGAGTTGCGGG
TTGAAATCTCGGATGCGGAAAAGTTGTCGACGGTGACTCGAAGTGATTCAGTCCCGC
CAAAGCTCATCCGTCCTTCGGGCAAAAGATGACGGTCAAGACCTCGTCCTTTCTCTCT
TTCCATTGCGTTTGAGAGGATGTGGCGGGGAATTGCCGTGATCGATGAATGCTACCTG
GTTGATCCTGCCAGTAGTCATATGCTTGTCTCAAAGATTAAGCCATGCATGTGTAAGTAT
GAACGAATTCAGACTGTGAAACTGCGAATGGCTCATTAAATCAGTTATAGTTTGTTTGAT
GGTAACTACTACTCGGATAACCGTAGTAATTCTAGAGCTAATACGTGCAACAAACCCCG
ACTTATGGAAGGGACGCATTTATTAGATAAAAGGTCGACGCGGGCTCTGGCTTGCTCT
GATGATTCATGATAACTCGACGGATCGCATGGCCTCTGTGCTGGCGACGCATCATTCA
AATTTCTGCCCTATCAACTTTCGATGGTAGGATAGTGGCCTACCATGGTGGTAACGGGT
GACGGAGAATTAGGGTTCGATTCCGGAGAGGGAGCCTGAGAAACGGCTACCACATCC
AAGGAAGGCAGCAGGCGCGCAAATTACCCAATCCTGACACGGGGAGGTAGTGACAAT
AAATAACAATACCGGGCTCTTTCGAGTCTGGTAATTGGAATGAGTACAATCTAAATCCCT
TAACGAGGATCCATTGGAGGGCAAGTCTGGTGCCAGCAGCCGCGGTAATTCCAGCTC
CAATAGCGTATATTTAAGTTGTTGCAGTTAAAAAGCTCGTAGTTGAACCTTGGGATGGG
TCGGCCGGTCCGCCTTTGGTGTGCATTGGTCGGCTTGTCCCTTCGGTCGGCGATACG
CTCCTGGTCTTAATTGGCCGGGTCGTGCCTCCGGCGCTGTTACTTTGAAGAAATTAGA

GTGCTCAAAGCAAGCCTACGCTCTGGATACATTAGCATGGGATAACATCATAGGATTTC
GATCCTATTGTGTTGGCTTCGGGATCGGAGTAATGATTAACAGGGACAGTCGGGGGCA
TTCGTATTTCATAGTCAGAGGTGAAATTCTTGGATTTATGAAAGACGAACAACTGCGAA
AGCATTTGCCAAGGATGTTTTCATTAATCAAGAACGAAAGTTGGGGGCTCGAAGACGA
TCAGATACCGTCCTAGTCTCAACCATAAACGATGCCGACCAGGGATCAGCGGATGTTG
CTTATAGGACTCCGCTGGCACCTTATGAGAAATCAAAGTTTTTGGGTTCCGGGGGGAG
TATGGTCGCAAGGCTGAAACTTAAAGGAATTGACGGAAGGGCACCACCAGGAGTGGA
GCCTGCGGCTTAATTTGACTCAACACGGGGAAACTTACCAGGTCCAGACATAGTAAGG
ATTGACAGACTGAGAGCTCTTTCTTGATTCTATGGGTGGTGGTGCATGGCCGTTCTTA
GTTGGTGGAGCGATTTGTCTGGTTAATTCCGTTAACGAACGAGACCTCAGCCTGCTAA
CTAGCTACGTGGAGGCATCCCTTCACGGCCGGCTTCTTAGAGGGACTATGGCCGTTTA
GGCCAAGGAAGTTTGAGGCAATAACAGGTCTGTGATGCCCTTAGATGTTCTGGGCCG
CACGCGCGCTACACTGATGTATTCAACGAGTTCACACCTTGCCGACAGGCCCGGGTA
ATCTTTGAAATTTCATCGTGATGGGGATAGATCATTGCAATTGTTGGTCTTCAACGAGG
AATTCCTAGTAAGCGCGAGTCATCAGCTCGCGTTGACTACGTCCCTGCCCTTTGTACA
CACCGCCCGTCGCTCCTACCGATTGAATGATCCGGTGAAGTGTTCGGATCGCGGCGA
CGTGGGTGGTTCGCCGCCCGCGACGTCGCGAGAAGTCCACTAAACCTTATCATTTAG
AGGAAGGAGAAGTCGTAACAAGGTTTCCGTAGGTGAACCTGCGGAAGGATCATTGTC
GATACCTGTCCAAAACAGAACGACCCGCGAACCAAAGATCACCACTCTCGGTGGGCC
GGTTTCTTAGCCGATTCCTTGCCCGCCGGATCCGTGGTTTCGCGTATCGGCATGATCG
GGAGCTTTTATCTCGGTCTTGTCGTGCGCGTTGCTTCCGGATATCACAAAACCCCGGC
ACGAAAAGTGTCAAGGAACATGCAAACGAACGGCTGGCATTCGCCTCCCCGGAGACG
GAGTGTGGGCGGATGCTGTGCTGCGAACTGAAGTCTAAAACGACTCTCGGCAACGGA
TATCTCGGCTCTCGCATCGATGAAGAACGTAGCGAAATGCGATACTTGGTGTGAATTG
CAGAATCCCGTGAACCATCGAGTCTTTGAACGCAAGTTGCGCCCCAAGCCTTCTGGC
CGAGGGCACGTCTGCCTGGGTGTCACAAATCGTCGTCCCTCACCATCCTTTGCTGAT
GCGGGACGGAAGCTGGTCTCCCGTGTGTTACCGCACGCGTTGGCCTAAATCCGAGC
CAAGGACGCCTGGAGCGTACCGACATGCGGTGGTGAACTTGATCCATTACATTTTATC
GGTCGCTCTTGTCCGGAAGCTGTAGATGACCCAAAGTCCATATAGCGACCCCAGGTC
AGGCGGGATTACCCGCTGAGTTTAAGCATATCAATAAGCGGAGGAAAAGAAACTAACA
AGGATTCCCTTAGTAACGGCGAGCGAACCGGGAAGAGCCCAGCTTGAAAATCGGACG
TCTTCGGCGTTCGAATTGTAGTCTGGAGAAGCGTCCTCAGCGACGGACCGGGCCTAA
GTTCCCTGGAAAGGGGCGCCAGAGAGGGTGAGAGCCCGTCGTGCCCGGACCCTGT
CGCACCACGAGGCGCTGTCTACGAGTCGGGTTGTTTGGGAATGCAGCCCCAATCGG
GCGGTAAATTCCGTCCAAGGCTAAATACGGGCGAGAGACCGATAGCGAACAAGTACC
GCGAGGTAAAGATGAAAAGGACTTTGAAAAGAGAGTCAAAGAGTGCTTGAAATTGTCG
GGAGGGAAGCGGATGGGGGCCGGCGATGCGTCCTGGTCGGATGCGGAACGGAGCA
ATCCGGTCCGCCGATCGATTCGGGGCGTGGACCGACGCGGATTACGGTGGCGGCCT
AAGCCCGGGCTTTTGATACGCTTGTGGAGACGTCGCTGCCGTGATCGTGGTCTGCAG
CACGCGCCTAACGGCGTGCCTCGGCATCAGCGTGCTCCGGGCGTCGGCCTGTGGG
CTCCCCATTCGACCCGTCTTGAAACACGGACCAAGGAGTCTGACATGTGTGCGAGTC
AACGGGTGAGTAAACCCGTAAGGCGCAAGGAAGCTGATTGGCGGGATCCTCGCGGG
TGCACCGCCGACCGACCTTGATCTTCTGAGAAGGGTTCGAGTGTGAGCATGCCTGTC
GGGACCCGAAAGATGGTGAACTATGCCTGAGCGGGGTAAAGCCAGAGGAAACTCTG
GTGGAAGCCCGCAGCGATACTGACGTGCAAATCGTTCGTCTGACTTGGGTATAGGGG
CGAAAGACTAATCGAACCATCTAGTAGCTGGTTCCCTCCGAAGTTTCCCTCAGGATAG
CTGGAGCTCGGACGCGAGTTCTATCGGGTAAAGCCAATGATTAGAGGCATTGGGGGC
GCAACGCCTCGACCTATTCTCAAACTTTAAATAGGTAGGACGTGTCGGCTGCTTTGTT
GAGCCGTCACACGGAATCGAGAGCTCCAAGTGGGCCATTTTTGGTAAGCAGAACTGG
CGATGCGGGATGAACCGGAAGCCGGGTTACGGTGCCCAACTGCGCGCTAACCTAGA
ACCCACAAAGGGTGTTGGTCGATTAAGACAGCAGGACGGTGGTCATGGAAGTCGAAA
TCCGCTAAGGAGTGTGTAACAACTCACCTGCCGAATCAACTAGCCCCGAAAATGGATG

GCGCTTAAGCGCGACCTATACCCGGCCGTCGGGGCAAGAGCCAGGCCTCGATGAGT
AGGAGGGCGCGGCGGTCGCTGCAAAACCTAGGGCGCGAGGCGCGGAGCGGCCGTC
GGTGCAGATCTTGGTGGTAGTAGCAAATATTCAAATGAGAACTTTGAAGGCCGAAGAG
GGGAAAGGTTCCATGTGAACGGCACTTGCACATGGGTTAGTCGATCCTAAGAGTCGG
GGGAAACCCGTCTGATAGCGCTTAAGCGAACTTCGAAAGGGGATCCGGTTAAAATTCC
GGAACCGGGACGTGGCGGTTGACGGCAACGTTAGGGAGTCCGGAGACGTCGGCGG
GGGCCTCGGGAAGAGTTATCTTTTCTGTTTAACAGCCTGCCCACCCTGGAAACGGCT
CAGCCGGAGGTAGGGTCCAGCGGCTGGAAGAGCACCGCACGTCGCGTGGTGTCCG
GTGCGCCCCCGGGCGCCCTTGAAAATCCGGAGGACCGAGTGCCGCTCACGCCCGG
TCGTACTCATAACCGCATCAGGTCTCCAAGGTGAACAGCCTCTGGTCGATGGAACAAT
GTAGGCAAGGGAAGTCGGCAAAATGGATCCGTAACTTCGGGAAAAGGATTGGCTCTG
AGGGCTGGGCTCGGGGGTCCCAGTTCCGAACCCGTCGGCTGTCAGCGGACTGCTC
GAGCTGCTTCCGCGGCGAGAGCGGGTCGCCGGCTGCCGGCCGGGGGACGACTGG
GAACGGCTCTCTCGGGAGCTTTCCCCGGGCGTCGAACAGTCAGCTCAGAACTGGTA
CGGACAAGGGGAATCCGACTGTTTAATTAAAACAAAGCATTGCGATGGTCCCTGCGGA
TGCTAACGCAATGTGATTTCTGCCCAGTGCTCTGAATGTCAAAGTGAAGAAATTCAACC
AAGCGCGGGTAAACGGCGGGAGTAACTATGACTCTCTTAAGGTAGCCAAATGCCTCGT
CATCTAATTAGTGACGCGCATGAATGGATTAACGAGATTCCCACTGTCCCTGTCTACTA
TCCAGCGAAACCACAGCCAAGGGAACGGGCTTGGCAGAATCAGCGGGGAAAGAAGA
CCCTGTTGAGCTTGACTCTAGTCCGACTTTGTGAAATGACTTGAGAGGTGTAGGATAA
GTGGGAGCTTCGGCGCAAGTGAAATACCACTACTTTTAACGTTATTTTACTTACTCCGT
GAATCGGAGGCCGGGGTACAACCCCTGTTTTTGGTCCCAAGGCTCGCTTCGGCGGG
TCGATCCGGGCGGAGGACATTGTCAGGTGGGGAGTTTGGCTGGGGCGGCACATCTG
TTAAAAGATAACGCAGGTGTCCTAAGATGAGCTCAACGAGAACAGAAATCTCGTGTGG
AACAAAAGGGTAAAAGCTCGTTTGATTCTGATTTTCAGTACGAATACGAACCGTGAAAG
CGTGGCCTATCGATCCTTTAGACTTCGGAATTTGAAGCTAGAGGTGTCAGAAAAGTTAC
CACAGGGATAACTGGCTTGTGGCAGCCAAGCGTTCATAGCGACGTTGCTTTTTGATCC
TTCGATGTCGGCTCTTCCTATCATTGTGAAGCAGAATTCACCAAGTGTTGGATTGTTCA
CCCACCAATAGGGAACGTGAGCTGGGTTTAGACCGTCGTGAGACAGGTTAGTTTTACC
CTACTGATGCCCGCGTCGCGATAGTAATTCAACCTAGTACGAGAGGAACCGTTGATTC
GCACAATTGGTCATCGCGCTTGGTTGAAAAGCCAGTGGCGCGAAGCTACCGTGCGCT
GGATTATGACTGAACGCCTCTAAGTCAGAATCCGGGCTAGAAGCGACGCATGCGCCC
GCCGCCCGATTGCCGACCCTCAGTAGGAGCTTAGGCTCCAAAGGCACGTGTCGTTG
GCTAAGTCCGTTCGGCGGAACGGTCGTTCGGACCGCCTTGAATTATAATTACCACCGA
GCGGCGGGTAGAATCCTTTGCAGACGACTTAAATACGCGACGGGGTATTGTAAGTGG
CAGAGTGGCCTTGCTGCCACGATCCACTGAGATTCAGCCCTTTGTCGCTAAGATTCGA
CCCTCCCCTAAATCACTCCAAAAAAAACAATCCCCAATTCTACACAAGTGTTTCTACAC
TAACAAAGCAACAGCTCCTTAACGAATTCCCAACTTTACACGAGCTCGTCTCTCGAGG
TTAAATGTTATTACTTGGTAAGATTCCGGACCTCGCCAAGTGTTTTGAAAACCCGCAAC
GCTCGCAAAGGTGGATAGTGAGAATAATAAGTGAAGAGACAGACTTGTCCAAAACGCC
CACCACGAAGGTGCATAGTGAGAAGAGTAAGTCAAGAGATAGACTTGTCCAAAAAGAA
ACGGAAGAGAAAGCGTGGGGAGACGCTCACGAAGGTGCATAGTGAGAAGAGTAAGT
CAAGAGACAGACTTGTTCGAAAAGAAACAGAAGAGAATGCTTGGGGTTACACTCACG
AAGGTGCATAGTGAGAAGAGTAAGTCAAGAGACAGACTTGTTCGAAAAGAAACAAAAG
AGAATGCTTGGGGAGATAGAAGTGTGAGATAGTTCTCAAGCTAAGAAAGTTGTAAAAG
CTAAGAACTAGCATCAAATGATGGATGAAACACAAGGTAGTTGTTGAAAAGTCAAACAC
TTGGTGATATGAACACAAACGTTCAATATGACAAACCCATGCCAAGTAAAGAGAAAATG
AAAACTGGTGATTGTTGCGGAAATCGTCCAGGATTCCTCGACCAGGACTTGAAATCGT
CGAGGGGAAAAAATCGGTTCCGAGGAATCGTCGATCCGGACTTGGAATCGTCGAGAA
AAGTTTACCGGGTCCGAGGATTTGTCGACCAGGAGTGGAAATCGTCGAGAAAAATCTA
TCGGGTCCGAGGAATCGTCGACCAGGACGAGGAATCGTCGACCGGGTCCGAGGATT
TGTCGACCAGGGGTTGAAATCGTCGACCAGGTCCGAGACTTCATCGACCGGGTCCGA

GGATTCGTCGACCAGGAGTGGAAATCGTCGAGAAAAATCTATCGGGTCCGAGGAATC
GTCGACCAGGACGAGGAATCGTCGACCGGGTCCGAGGATTTGTCGACCAGGTCCGA
GACTTCATCGACCGGGTCCGAGGATTCGTCGACCAGGTCCGAGACTTCATCGACCGG
GTCCAAGTATTTGATTTTATTTTGGAACCGGTGTCTCCTCAGACATTTCAATGTATGTTG
GTGCCAAGAGGGAAAAGGGCTATAAAGCTATATAGGGGTGGGTGTGTGTAGTGGGAAT
TTTGCCCGCACGCGCGCGCGCGAATAAGAATAAGAATAAGTAATAAGAATAAGAATAAG
AAAAAGAAAAAAAAAAAAAAAAAAAAAAAAAACAAAAACTCCATAGAGTTGTTTTCTCAATC
GGGTCCGAGGAATCGTCGATCTGGACTTGGAATCGTCGAGAAAAGTTTACCGGGTCC
GAAAATTTGTCGACCAGGAGTGGAAATCGTCGAGAAAAATCTATCGGGTCCGAGGAAT
CGTCGACCAGGACGAGGAATCGTCGACCGGGTCCGAGGATTTGTCGACCAGGGGTT
GAAATAGTCGACCAGGTCCGAGACTTCATCGACCGGGTCCGAGGAATCGTCGACCAG
GAGTGGAAATCGTCGAGAAAAATCTATCGGGTCCGAGGAATCGTCGACCAGGACGAG
GAATCGTCGACCGGGTCCGAGGATTTGTCGACCAGGTCCGAGACTTCATCGACCGG
GTCCGAGGATTCGTCGACCAGGTCCGAGACTTCATCGACCGGGTCCAAGTATTTGATT
TTATTTTGGAACCGGTGTCTCCTCAGACATTTCAATGTATGTTGGTGCCAAGAGGGAAA
AGGGCTATAAAGCTATATAGGGGTGGGTGTGTGTAGTGGGAATTTTGCCCGCACGCGC
GCGCGCGATAAGAATAAGAATAAGTAATAAGAATAAGAATAAGAAAAAGAAAAAAAAAAA
AAAAAAAACAAAAACAAAAACTCCATAGAGTTGTTTTCTCAATCGGGTCCGAGGAATCG
TCGATCTGGACTTGGAATCGTCGAGAAAAGTTTACCGGGTCCGAAAATTTGTCGACCA
GGAGTGGAAATCGTCGAGAAAAATCTATCGGGTCCGAGGAATCGTCGACCAGGACGA
GGAATCGTCGACCGGGTCCGAGGATTTGTCGACCAGGGGTTGAAATCGTCGACCAG
GTCCGAGACTTCATCGACCGGGTCCGAGGAATCGTCGACCAGGACGATGAATGGTCG
ATGAAAATCTATCGGGTTCGAGGAATGGTCGACCAGGGGTTGAAATCGTCGACCAGG
TCCGAGACTTCATCGACCGTGTCCGAGGAGTGGTCGAGGGTTTGTCGACCAGGACG
AGGAATCGTCGACCGGGTCCGAGGATTTGTCGACCAGGGGTTGAAATCGTCGACCAG
GACCGAGAATTCGTCGACCAGGACGGCGGAACCCTCGACCAGGACGATGAATGGGC
GATGAAAATCTATCGGGTTCGAGGAATGGTCGACCAGGGGTTGAAATCGTCGACCAG
GTCCGAGACTTCATCGACCGGGTCCGAGGATTCGTCGACCAGGACGGCCGGATGTC
CGAGAAAAAAAAAATGTTGCCGAATAACTTTCGAAAATCATTGGATATGATGCAATGTTTT
GTGATCGAATCTCTTAAAATACATCAATAAAGAGTTTAGGATGTCAAGTTTGCATCAAAT
ATGCCCACGGAGCCCCAACTAGACCATGAAAATCCGATGTTGTATCAGGTCAAATGAC
CTAGCTAGAGGTGTCAAAAAATTATGAAAATTTACCAGAAAATAGGATTTAGTATCCTTAT
GATGCATGCCAAAAAGAATTTTCAAATTCCAAGTATTTCTTTTTTCTTGGCACCGGTGTC
TCCTCAGACATTTCAATGTCTGTTGGTGCCAAGAGGGAAAAGGGCTATTAAGC
