# Supplementary material for: 45S rDNA Diversity In Natura as One Step towards Ribosomal Heterogeneity in Arabidopsis thaliana
Source: Plants (Basel). 2023 Jul 21;12(14):2722. doi: 10.3390/plants12142722 (PMC10386311; doi:10.3390/plants12142722)
Supplement: Supplementary file 1 [file plants-12-02722-s001.zip › Table_S1_and_S2_legend.pdf]

Table S1: 45S rDNA copy number variation in At66. 18S and 25S copy number variation for 173 individuals of At66 (table as a csv file). Copy number variation was estimated by dividing the average coverage along both 18S and 25S rRNA genes by the average coverage along the first 10Mb of chromosome 3 [11].

Table S2: Variation at the 5' and 3'ETS in At66. The first two sheets of the table indicate the proportion of each 5R genotype in each site of At66 either expressed as a percentage [Table 5R genotypes (%)] or assigned attribute required for the QGIS software 3.28 (https://www.qgis.org/ accessed on 21 october 2022) [Table 5R genotypes (QGIS attributes)]. N indicates the number of genotyped individuals per site.

Sheets 3-4 indicate the proportion of each 3R genotype in each site of At66 either expressed as a percentage [Table 3R genotypes (%)] or assigned attribute required for the QGIS software 3.28 (https://www.qgis.org/ accessed on 21 october 2022) [Table 3R genotypes (QGIS attributes)]. N indicates the number of genotyped individuals per site. Sheets 5-6 represent a matrix connecting both 5R and 3R genotypes for the 320 individuals of At66 and subsampling environmental conditions along the altitudinal gradient either sea level compared to altitude (760-1900 m) or sea level compared to mid (760-1000 m) and high altitude (1500-1900 m).
